# RAD51B-EZH2 axis as a potential therapeutic target for TNBC through cell fate conversion

**DOI:** 10.1038/s41419-025-08259-8

**Published:** 2025-11-30

**Authors:** Shiqi Lin, Dongyang Tang, Josh Haipeng Lei, Xiangpeng Chu, Lijian Wang, Kai Miao, Ping Chen, Jingbo Zhou, Aiping Zhang, Ling Li, Heng Sun, Xiaoling Xu, Chuxia Deng

**Affiliations:** 1 MOE Frontier Science Center for Precision Oncology, University of Macau, Macau SAR, China; 2 Cancer Center, Faculty of Health Sciences, University of Macau, Macau SAR, China; 3 Thrust of Bioscience and Biomedical Engineering, Brain and Intelligence Research Institute, The Hong Kong University of Science and Technology (Guangzhou), Guangzhou, PR China; 4 Guangdong Key Laboratory of New Technology in Rice Breeding, Rice Research Institute, Guangdong Academy of Agricultural Sciences, Guangzhou, China; 5 Zhuhai UM Science &Technology Research Institute, Hengqin, Zhuhai, Guangdong China

**Keywords:** Breast cancer, Cancer therapy

## Abstract

Triple-negative breast cancer (TNBC) is the most aggressive subtype of breast cancer with higher histologic grade, poorer prognosis, and fewer treatment options due to the lack of reliable and effective molecular targets. Using a functional approach with the Sleeping Beauty (SB) transposon system, we have identified 64 overlapped candidate driver genes for inducing TNBC formation in *Brca1*-deficient mice and *Fgfr2*-mutant mice. Further analysis reveals that *Rad51b* deficiency leads to the development of tumors with a TNBC phenotype by repressing ERα expression through the recruitment of polycomb repressive complex 2 (PRC2) and subsequent trimethylation of histone H3 lysine 27 in *Esr1* promoter region. Mechanistically, the loss of RAD51B upregulated cellular ATP levels, followed by the suppression of the AMP-activated protein kinase (AMPK) pathway and dephosphorylation of the Enhancer of zeste homolog 2 (EZH2) at the Thr311 region, which enhances the assembly of PRC2 to repress expression of *Esr1*. Inhibition of the RAD51B-EZH2 axis allows the re-expression of functional ERα, making TNBC targetable by endocrine therapy. Consistently, the combination of EZH2 inhibitor with tamoxifen effectively reduces TNBC progression, suggesting that the RAD51B-EZH2 axis is a potential therapeutic target for TNBC.

## Introduction

Breast cancer is the most commonly diagnosed cancer among women worldwide, having surpassed lung cancer for the first time in 2020 [1]. The choice of therapeutic approaches for breast cancer primarily depends on the clinical and molecular characteristics of the tumors [2]. Triple-negative breast cancer (TNBC), accounting for ~15–20% of breast cancer cases, is classified as triple-negative for estrogen receptor (ER), progesterone receptor (PR) and human epidermal growth factor receptor 2 (HER2). Consequently, endocrine therapy and HER2-targeted therapy are ineffective for TNBC, coupled with the inherently aggressive clinical behavior, resulting in a higher rate of distant recurrence and a poorer prognosis compared to other subtypes [3, 4]. TNBC itself can also be classified into at least six subtypes with distinct expression patterns, including basal-like 1 (BL1), basal-like 2 (BL2), immunomodulatory (IM), mesenchymal (M), mesenchymal stem-like (MSL), and luminal androgen receptor (LAR) based on their association with some signaling pathways and gene expression signatures [5–8]. Despite the discovery of many signature genes for TNBC and its subtypes, limited genetic oncogenic drivers of TNBC have been identified [5, 6, 9]. Therefore, new technologies that can identify drivers and explore the mechanisms underlying the tumorigenesis and progression of TNBC are crucial for discovering new therapeutic targets for this deadly cancer.

The breast cancer-associated gene 1 (*BRCA1*) is a well-known tumor suppressor gene, whose germline mutation is responsible for 20–30% of hereditary breast cancers and ~2–4% of total breast cancer cases. Inherited mutation in *BRCA1* gene predisposes female carriers to early onset tumorigenesis and up to 87% cumulative lifetime risk of developing breast cancer [7, 8]. BRCA1-associated breast cancers also have higher risk to form TNBCs as an ~48% of *BRCA1* mutation carriers develop TNBCs, compared with 12% of non-carriers [9]. To study functions of BRCA1 in breast cancer formation, we had previously generated mouse models carrying mammary-specific disruption of *Brca1* using Cre-loxP system driven by *Wap-Cre* or *MMTV-Cre* [10]. Approximately 25% of *Brca1* mutant mice with either *Brca1*^*co/co*^*;Wap-Cre* or *Brca1*^*co/co*^*;MMTV-Cre* developed mammary tumors at about 18 months, and the tumorigenesis could be accelerated by impaired function of p53, which suppresses lethality caused by *Brca1* deficiency [10, 11]. We have also demonstrated earlier that *Brca1* mutant tumor are largely ERα positive at the initiation stages and gradually become ERα negative during their progression [12], however the molecular changes responsible for the conversation from ERα positive to negative remains elusive.

Fibroblast growth factor receptor 2 (*FGFR2*) belongs to a family of four membrane-bound receptor tyrosine kinases that mediates signaling for FGFs [13]. Multiple genetic aberrations in FGFR2, which activate upstream and/or downstream signaling pathways, have been identified in breast cancer. For instance, in an analysis of 4398 familial breast cancer cases and 4316 controls, five single-nucleotide polymorphisms (SNPs) (rs7895676, rs2912781, rs10736303, rs2912778 and rs2981582) in FGFR2 were found to be significantly associated with breast cancer [14]. Meanwhile, a study also identified four additional SNPs in intron 2 of FGFR2 (rs11200014, rs2420946, rs1219648 and rs2981579) that linked to increased breast cancer susceptibility [15]. Furthermore, FGFR2 amplification (10q26.3) was detected in 6 out of 165 TNBC cases (3.6%) [16], but not in other subtypes (0/214, *p* = 0.00065). Researchers screened a panel of 51 breast cancer cell lines and identified two cell lines, MFM223 and SUM52PE, that exhibited FGFR2 gene amplification and protein overexpression. Both MFM223 and SUM52PE were found to be TNBC cell lines, and the amplification of FGFR2 causes PI3K-AKT pathway activation and subsequent apoptosis inhibition [16]. We had previously generated a mouse strain carrying *Fgfr2*^pLoxPneo-Ser250Trp^ (S252W), which corresponds to the human FGFR2-S252W mutation with enhanced ligand-binding capability [17]. We demonstrated that activation of this point mutation in mice results in phenotypes mimicking human Apert syndrome (AS), which displays severe craniosynostosis, infertility, and poor health. We further generated *Fgfr2* mammary gland-specific point mutation (*Fgfr2-S252W;MMTV-Cre*) mouse model and provided the functional validation that activation of FGFR2 signaling could induce mammary tumorigenesis [18]. However, the functional role of FGFR2 in breast cancer, particularly in triple-negative subtypes, remains poorly understood.

Tumorigenesis can be accelerated by using the Sleeping Beauty (SB) DNA transposon system to induce tumors in experimental animals in a tissue-specific manner, aiming to uncover the genetic basis of various cancers [19–22]. The SB transposon system triggers cancer formation by randomly inserting into the mouse genome, thereby mutating or disrupting potential driver genes. Meanwhile, transposon insertions serve as molecular tags for high-throughput sequencing, which help to identify candidate cancer driver genes and provide insights into tumor evolution on a scale that is not yet possible through the sequencing of human tumors. In a previous study [22], we identified 169 candidate genes that trigger tumor formation by integrating the SB system into the *Cre-LoxP*-mediated *Brca1* mammary gland-specific knockout (*Brca1*^*co/co*^*;Wap-Cre;SB* and *Brca1*^*co/co*^*;MMTY-Cre;SB*) mouse model. We pinpointed *Notch1* as a top putative oncogene, whose activation compensates for *Brca1* deficiency and enhances tumorigenesis. This finding validates the SB mutagenesis system as a powerful tool for uncovering novel cancer driver genes across diverse tumor types. We also introduced the SB system into *Fgfr2* mammary gland-specific point mutation (*Fgfr2-S252W;MMTV-Cre*) mouse model to perform functional-based driver gene screening, aiming to identify genes that contribute to *Fgfr2*-mediated tumorigenesis. Therefore, we have collected a cohort of breast cancer tumors from *Brca1*^*co/co*^*;Wap-Cre;SB* mice and *Fgfr2-S252W;MMTV-Cre;SB* mice, providing a valuable resource for identifying TNBC-associated genetic drivers.

In our efforts to identify the genetic driver of TNBC, we analyzed TNBC tumors developed in the *Brca1*^*co/co*^*;Wap-Cre;SB* mice and *Fgfr2-S252W;MMTV-Cre;SB* mice. We found that the loss of function of RAD51B may serve as a driver in TNBC development. Mechanistically, *Rad51b* deletion enhances cellular ATP levels and suppresses the activation of AMP-activated protein kinase (AMPK), leading to the decrease in phosphorylation at the Thr311 region of EZH2 protein and facilitate the assembly of polycomb repressive complex 2 (PRC2). Furthermore, *Rad51b* deletion induces the recruitment of the PRC2 complex to the ERα promoter region, which catalyzes the trimethylation of histone H3 at lysine 27, resulting in decreased ERα expression during tumor progression. Thus, the specification of TNBCs is under epigenetic control and can be therapeutically targeted to sensitize them to endocrine therapy.

## Experimental section

### Animal experiments

All animal experiments were conducted under the supervision of the University of Macau Animal Ethics Committee (UMARE-015-2019). All surgical procedures were conducted in accordance with the requirements of the Animal Care and Use Committee of the Faculty of Health Sciences at the University of Macau.

### Cell lines and cell culture

Human breast cancer cell lines (MCF-7, MDA-MB-231, and T47D) were obtained from American Type Culture Collection (ATCC) and cultured in Dulbecco’s modified Eagle medium (DMEM, Gibco, Thermo Fisher Scientific) containing 10% fetal bovine serum (FBS) and 1% glutamine under 5% carbon dioxide. Human breast cancer cell line HCC1937 and mouse breast cancer cell lines (4T1 and EMT6) were cultured in Roswell Park Memorial Institute (RPMI) 1640 medium containing 10% FBS and 1% glutamine under 5% carbon dioxide. SUM149 cells were cultured in DMEM/F12 medium containing 10% FBS, 1% glutamine, 10 µg/ml insulin and 1 µg/ml hydrocortisone under 5% carbon dioxide. Mouse epithelial cell lines B477 and G600 were derived from the mammary gland of *Brca1*-WT mouse and *Brca1*-MT mouse, respectively, as previously described [23] and cultured in DMEM medium. Mouse primary mammary tumor cells HP5008 were derived from *Fgfr2* mutation mice mammary gland with a single spontaneously arising tumor (ERα+, PR−, Her2−) at the age ~12 months. The 545 cells were derived from *Brca1*^*co/co*^ *MMTV-Cre* mice mammary gland with a spontaneously arising tumor (ERα−, PR−, Her2−). HP5008 cells were cultured in DMEM medium, and 545 cells were cultured in F-medium.

### Plasmid construction

LentiCRISPRv2 (Addgene #52961), LentiCRISPRv2 blast (Addgene #98293), LentiCRISPRv2 hygro (Addgene #98291), TLCV2 (Addgene #87360) were obtained from Addgene. Plasmids were digested with the restriction enzyme Esp3I (New England Biolabs) and ligated with the annealed sgRNA paired DNA sequences. ERE-Luc (Addgene #11354), PRE-Luc (Addgene #11350) and pNeuLite (Addgene #16247) were purchased from Addgene. The p-GF-ERE-Luc plasmid was purchased from System Biosciences (Cat# TR205PA-P).

### Generation of gene knockout cells

Gene knockout cells were generated using CRISPR-Cas9 technology. Guide RNAs (gRNAs) were cloned into LentiCRISPRv2, LentiCRISPRv2 blast, or LentiCRISPRv2 hygro according to the standard protocol. Sequences were summarized in Supplementary Table S1. Lentivirus packing was conducted by co-transfecting 4 μg lentiviral vector, 3 μg psPAX2 (Addgene #12260), and 2 μg pMD2.G (Addgene #12259) into HEK293T cells using PEI. The vial supernatants were collected after 48 h of transfection and used to infect. Cell culture medium was replaced with culture medium containing suitable antibiotics (InVivoGen) 48 h later. Cells were collected when there were no visible clones in the control group. Knockout efficacy was validated through Western blot or RT-qPCR. In the luciferase reporter screening assay, cells were co-transfected with two sgRNAs per target gene.

For the generation of inducible gene knockout cells, individual sgRNA were cloned into the all-in-one pLentiCRISPRv2 (TLCV2)-lentiviral vector (Addgene #87360) using the same protocol provided with LentiCRISPRv2 [24].

### 3D tumor slice culture

Cancer cells were injected into the mammary gland of 4–6-week-old female nude mice to form a tumor. Tumors were collected and cut into tissue slices using Leica VT1200 S (Leica Biosystems, Nussloch GmbH, Germany). Buffer A (Rat Collagen I), buffer B (10× Ham’s F-12), and buffer C ((sterile reconstitution buffer, weight 2.2 g NaHCO3 to water and add NaOH and HEPES to a final concentration of 0.05 M and 200 mM. The final volume is 100 ml) were mixed at 8:1:1 (v/v/v). 50 μl mixed solution was added into each 12 mm diameter Millicell insert (Millipore, PIHP01250) and put at 37 °C for 30 min. Tumor slice was gently placed on the top of the lower gel in the insert. Then, another 50 μl mixed solution was added on top of the tumor slice to form the upper gel. The culture medium is the same as previously described [25], containing the indicated concentration of drug. Medium was changed on day 4. 7 days later, tumor slice was stained with 0.5 mg/ml MTT (Sigma) for 4 h at 37 °C (*n* = 3 slices per group). Images were taken with a Leica M165FC fluorescent stereo microscope. To check viability, slices after MTT staining were put into DMSO to dissolve the formazan and then detected for optical density at 490 nm using a PerkinElmer Victor X3 Microplate Reader.

### Organoid culture and passage

Patient-derived organoids were cultured or passaged based on previously described methods [26]. For drug treatment experiments, organoids were resuspended in cold trypsin after removing the culture medium and incubated at 37 °C for 5 min. Cells were spun down, washed with PBS and resuspended for further drug treatment experiments as previously described [26]. This experiment was assessed and approved by the ethics committees at the University of Macau.

### Chemicals and drugs

EPZ6438 (Tazemetostat) and GSK343 were purchased from Selleckchem. Actinomycin A and estradiol were purchased from Sigma-Aldrich.

### Immunohistochemistry staining assay

All tumor tissues were fixed in 10% neutral buffered formalin solution (Sigma-Aldrich) and then dehydrated, embedded in paraffin, and sectioned at a 4 µm thickness. After deparaffinization and rehydration, antigen retrieval and quenching of endogenous peroxidase, slides were blocked with animal-free blocker for 1 h at room temperature, then incubated with the indicated primary antibody overnight at 4 °C. Slides were washed three times using PBS and incubated with Biotinylated Goat anti-Mouse and Rabbit IgG (H + L) (abcam, ab64257) for 10 min at room temperature. After three PBS washes, the slides were applied with Streptavidin-HRP (abcam, ab64269) for 30min at room temperature. Slides were placed in DAB substrate (abcam, ab64238) and incubated for 3–5 min, counterstained with hematoxylin and dehydrated. A coverslip with Permount Mounting Medium (Fisher Scientific) was added to each slide, and the slides were scanned using NanoZoomer S60 digital slide scanner (Hamamatsu). The primary antibodies used in this assay were showed as follow: ERα (1:200, Abcam Cat# ab241557), PR (1:200, Abcam Cat# ab101688), Her2 (1:200, CST #2165), Rad51b (1:200, Invitrogen Cat# PA5-101336), H3K27me3 (1:200, CST #9733), H3K9me3 (1:200, CST #13969).

### Hematoxylin and Eosin staining assay

Tissues were perfused with 10% neutral buffered formalin solution and then dehydrated, embedded in paraffin and sectioned for staining. Slides were first deparaffinized and rehydrated to distilled water, then stained in Hematoxylin for 1 min and rinsed with tap water for 2 min. Slides were dipped in acid alcohol, washed with tap water for 2 min, stained with Eosin for 1 min and dehydrated. A coverslip with Permount Mounting Medium (Fisher Scientific) was added to each slide, and the slides were scanned using NanoZoomer S60 digital slide scanner (Hamamatsu).

### Luciferase assay

Cells were seeded onto a 24-well plate (20,000 cells per well) and allowed to adhere overnight. Cells were transfected with 0.4 µg ERE-Luc, PRE-Luc or pNeuLite reporter together with 0.1 µg Renilla for 48 h using Lipofectamine^TM^ 3000 following the protocol. Following the manufacturer’s protocol, the reporter signals were measured by using the Dual-Luciferase Reporter Assay System Kit (Promega# E1910) and reflected as the Luc/Ren ratios. Data are represented for three independent experiments.

### Western blotting

Proteins were extracted from cells or tumor tissues using RIPA lysis buffer supplemented with phosphatase and protease inhibitor cocktails. Protein concentrations were measured using the BCA protein assay kit. The proteins were separated by SDS-PAGE and electro-transferred to PVDF. After blocking with 3% BSA in TBS-T buffer for 1 h, membranes were incubated with the indicated primary antibody overnight at 4 °C. Membranes were washed three times before incubating with the corresponding secondary antibody (1:5000, Cell Signaling Technology, CST7076 or CST7074) for 40 min at room temperature. The immunoreactive bands were visualized using the ECL substrate (Bio-Rad) on a ChemiDoc MP system (Bio-Rad). The primary antibodies used in this assay were showed as follow: β-actin (1:1000, CST #3700), GAPDH (1:1000, CST #5174), ERα (1:1000 Abcam ab241557), PR (1:200, Santa Cruz, sc-538), Her2 (1:1000, CST #2165), H3K27me3 (1:1000, CST #9733), H3K9me3 (1:1000, CST #13969), Histone 3 (1:1000, CST #9715), Ezh2 (1:1000, CST #5246), Suz12 (1:1000, CST #3737), Aebp2 (1:1000, CST #14129), β-Tubulin (1:1000, CST #2146), phosphor-Ezh2 (Thr345) (1:1000, Affinity Cat# AF3584), phosphor-Ezh2 (Thr416) (1:1000, Affinity Cat# AF3585), phosphor-Ezh2 (Thr311) (1:1000, CST #27888S), Ampkα (1:1000 CST #2532S), phosphor-Ampkα (1:1000 CST #2535S). Full and uncropped western blots are presented in the Supplemental File.

### Cell viability analysis

Cells were seeded onto a 96-well plate (5000 cells per well) and allowed to adhere overnight. After indicated drug treatment for 48 h, Cell viability was measured by Alamar Blue assay. Briefly, the culture medium was changed to a medium containing 0.02% Alamar Blue for 2 h incubation at 37 °C. The absorbance at 590 nm under 560 nm excitation was measured by PerkinElmer Victor X3 Microplate Reader (Thermo Fisher Scientific).

### Colony formation assay

100 cells were seeded into a six-well plate and incubated for around 21 days until visible colonies formed. Colonies were fixed with 4% formaldehyde, stained with 0.5% crystal violet, and scanned using Bio-Rad ChemiDoC Imaging Systems.

### ChIP assay

Grow cells (treated or untreated) to 80% to 90% confluence on the plate and add 1% formaldehyde/culture medium for cross-linking at room temperature. After 10-15 min incubation, 1.25 M glycine was added, and cells were collected using a cell scraper. Centrifuge the cells at 1000 rpm for 3 min, discard the supernatant, wash cells by resuspending them in 10 ml of ice-cold PBS and centrifuge at 1000 rpm for 5 min. Keep the cell pellet for cell lysis and chromatin extraction by the Chromatin Extraction Kit (Abcam Cat# ab117152). The chromatin solution was further sent for One-Step ChIP Reaction, followed by the manufacturer’s protocol. The primary antibodies used in ChIP assay were showed as follow: H3K27me3 (CST #9733S), Ezh2 (CST #5246), Suz12 (CST #3737), Aebp2 (CST #14129), Non-Immune IgG supplied in ChIP Kit Magnetic-One-Step (Abcam Cat# ab156907) was served as negative control. After antibody precipitation, the solution was removed using a magnetic stand. 1X Wash Buffer was added to resuspend the beads and then removed the solution. Wash each reaction once with DNA Release Buffer using the same wash procedure as above. After washing, DNA Release Buffer-PK was added to each reaction and incubated at 65 °C for 15 min and then 95 °C for 5 min. Pelleted the beads using a magnetic stand and transferred the supernatant to the new PCR tube for further analysis.

### Quantitative real-time PCR

RNA was isolated from cultured cells and tumor tissue using TRIzol reagent (Invitrogen) and reverse-transcribed into cDNA using QuantiTect Reverse Transcription kit (Qiagen, 205313). Quantitative RT-PCR was conducted using FastStart Universal SYBR-Green Master (Rox) mix (Sigma) on QuantStudio 7Flex Real-Time PCR System (Thermo Fisher). The Primers used are listed in Supplementary Table S2.

### Cellular ATP, ADP/ATP ratios assay

Cellular ATP levels and ADP/ATP ratios were detected using ADP/ATP ratios assay kit (Sigma-Aldrich, MAK135). Following the manufacturer’s protocol, cells with or without dox treatment were collected to calculate the cell number. A total of 10,000 cells per experimental group were used for further ATP levels detection. ATP Reagent was added for 1min incubation, and read luminescence for the ATP assay (RLU_A_). After an additional 10 min incubation at room temperature, read luminescence for ATP (RLU_B_). ADP Reagent was added for 1 min incubation, and read luminescence (RLU_C_). Calculating the ADP/ATP ratios using the formula below: ADP/ATP ratios = (RLU_C_-RLU_B_)/RLU_A_.

### Immunofluorescence assay

Cells were fixed with 4% formaldehyde in PBS for 10 min, permeabilized with 0.05% Triton X-100 in PBS for 15 min, washed three times with PBS and blocked with 3% BSA in PBS-T for 30 min. Samples were incubated with primary antibody overnight at 4 °C, washed with PBS-T three times and incubated with secondary antibodies for 1 h at room temperature. After three PBS-T washing, a drop of ProLong Glass Antifade Mountant with NucBlue Stain (Invitrogen) and a coverslip was placed, and images were captured with a microscope. The primary antibodies used in this assay were shown as follows: H3K27me3 (1:1000, CST #9733S)

### Allograft studies

4–6-week-old BALB/c or nude mice were anesthetized, and a small incision was made. Single-cell suspensions were injected into mammary fat pads. When tumor reached a mean volume of ~50 mm^3^ (length X width^2^/2), the mice were randomly assigned to experimental treatment groups and began receiving drug treatment (*N* = 6 mice per group). EPZ6438 (Selleckchem Cat#S7128) and GSK343 (MedChemExpress Cat#HY-13500) were administered orally every day at 20 mg/kg and 10 mg/kg, respectively. Tamoxifen (MedChemExpress Cat#HY-13757A) was administered by intraperitoneal injection at 10 mg/kg, and the control group was treated with solvent (saline containing 5% DMSO, 40% PEG400 and 5% Tween80).

In doxycycline (dox) -regulated allograft systems for inducible gene knockout, mice were treated with dox in drinking water (2 mg/ml) every day.

### RNA-sequencing and data analysis

Cells or tissues were collected and stored in Trizol, then sequenced by Novogene (Tianjian, China). Quality check was conducted using MultiQC (v1.11) [27]. RNA reads were aligned to the mouse reference genome and counted by featureCounts (2.0.1) [28]. Genes that showed differentially expressed were identified with DESeq2 [29] with the following criteria: log2 fold change>1, *p* value < 0.05, and FDR (padj) value < 0.05. Gene Set Enrichment Analysis (GSEA) terms analysis was performed using GSEA software from Broad Institute [30]. The results were filtered by absolute NES value > 1, *p* value < 0.05, and FDR value < 0.05.

### Key resources

The detailed information of reagents or resources used in this paper is listed in Supplementary Table S3.

### Quantification and statistical analysis

Data are presented as means ± SEM. TIC frequency and statistical significance were determined by Pearson’s Chi-square test using ELDA software. Statistical evaluations were performed using GraphPad Prism software (version 10.0)

## Results

### Loss of function of RAD51B serves as oncogenic driver for TNBC formation

**Fig. 1:**
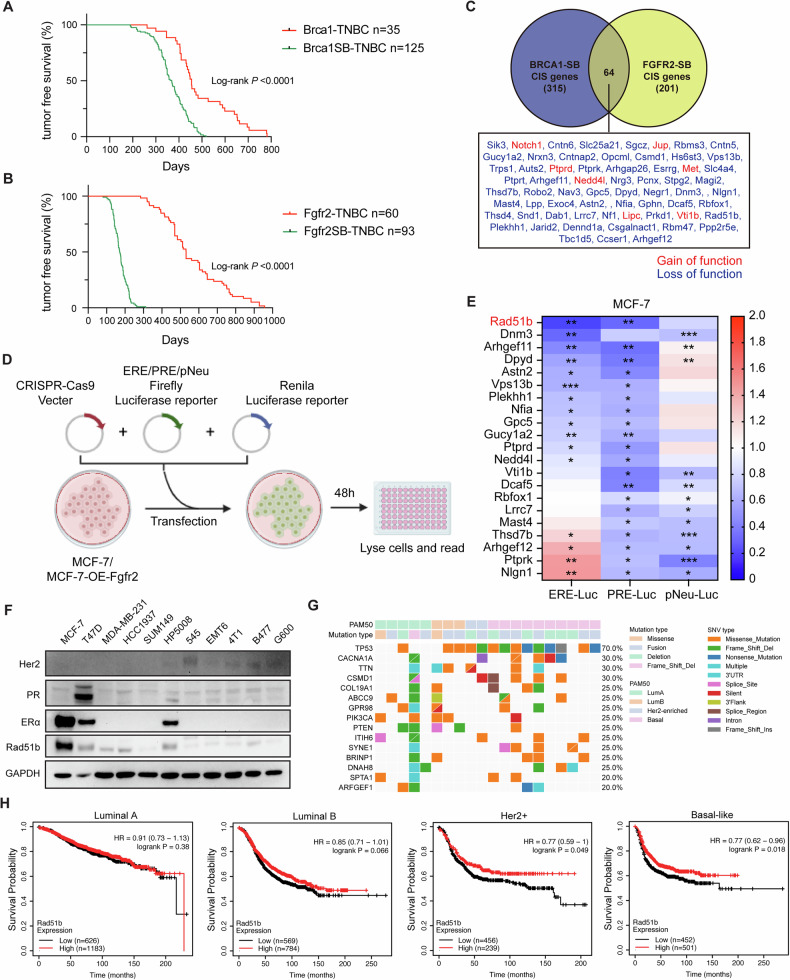
Identification of RAD51B as a candidate driver for TNBC formation. **A** Kaplan–Meier curve showing the mammary tumors-free rate for mice with TNBC tumors in *Brca1*^*Co/Co*^*; Wap-Cre* and *Brca1*^*Co/Co*^*; Wap-Cre; SB; T2Onc3* group. **B** Kaplan–Meier curve showing the mammary tumors-free rate for mice with TNBC tumors in *Fgfr2*^*S252W/+*^*; MMTV-Cre* and *Fgfr2*^*S252W/+*^*; MMTV-Cre; SB; T2Onc3* group. **C** A Venn diagram indicates overlapped candidate TNBC driver genes between *Fgfr2*^*S252W/+*^*; MMTV-Cre; SB; T2Onc3* mice and *Brca1*^*Co/Co*^*; Wap-Cre; SB; T2Onc3* mice. **D** Schematic of the reporter screening assays to identify candidate TNBC driver genes. **E** Heatmap reveals that *Rad51b* was listed as the most effective TNBC driver gene. *P < 0.05, **P < 0.01, ***P < 0.001. **F** The expression of RAD51B in human and mouse breast cancer cell lines was measured by western blotting. **G** Identification of 20 tumors from patients with RAD51B loss-of-function mutations and their molecular subtypes from the TCGA database. **H** Kaplan–Meier survival plots showing overall survival of breast cancer patients with high or low RAD51B expression.

We conducted a functional approach to identify potential drivers for TNBC formation in two mouse models, *Brca1*^*co/co*^*;Wap-Cre;SB* mice (BrWSB) and *Fgfr2-S252W;MMTV-Cre;SB* mice (FrMSB) (Fig. S1A). Using antibodies of ERα, PR or HER2 for immunohistochemistry (IHC) staining (Fig. S1B), we obtained 138 TNBCs from 308 tumors developed in BrWSB and 191 TNBCs in 932 tumors developed in the FrMSB mice, respectively. The formation of TNBCs in the SB mice was significantly accelerated compared with that of parental mice, as revealed by Kaplan–Meier tumor-free survival curves (Fig. 1A, B). The high-throughput sequencing for transposon insertion sites identified 10755 and 5404 genes in *Brca1*^*co/co*^*;Wap-Cre* and *Fgfr2-S252W;MMTV-Cre* mice, respectively, whose alterations were associated with accelerated TNBC formation. Using a cut-off point of their appearance in at least 5% of tumors in both models, we narrowed down 379 and 265 candidate genes in the BrWSB model and FrMSB, respectively (Fig. S1A). Combining these two groups together yielded 64 overlapping genes that served as our candidate TNBC driver genes (Fig. 1C). Visual analysis of insertion patterns of the 64 candidate driver genes suggested that majorities (*n* = 57, 89%) carried loss-of-function mutations in SB tumors, which is similar to what has been observed in other SB mutagenesis screens performed in solid tumors [31–33]. Some of these 64 candidate genes, such as *Jup*, *Met*, *Nf1* and *Trps1*, have also been reported as drivers in other published SB screens for TNBC [19]. This consistency supports the reliability of our method for identifying candidate driver genes in TNBC.

Since TNBC is known to lack ERα, PR and HER2 expression, we first hypothesized that TNBC driver genes might influence ERα, PR or HER2 expression during tumor progression. Therefore, we conducted the luciferase-reporter screening assay using estrogen-response-element- (ERE−) Luc, progesterone-response-element- (PRE-) Luc and pNeuLite reporters to reflect the status of ERα, PR and HER2, respectively. Briefly, we knocked out our candidate genes one by one in MCF-7 cells and performed a luciferase reporter screening assay as described (Fig. 1D). To pinpoint our candidate TNBC driver gene, we picked up the genes that significantly decreased luciferase activities in each group. Results indicate that *RAD51B* knockout exhibited the strongest inhibitory effects on luciferase activities (Figs. 1E and S1C–E). We also performed the screening experiments in FGFR2-overexpressing MCF-7 cells and found that *Rad51b* knockout was the most effective in reducing the luciferase signal (Fig. S1F–H). These data suggest that *Rad51b* knockout may affect the status of ERα, PR or HER2 in cancer cells and could serve as the universal driver for TNBC formation.

We then looked back at the details of *Rad51b*-driven tumors in SB transposon models (Fig. S2A). The insertion sites of the SB transposon spread across the entire *Rad51b* locus with either in the same or inverse orientation at a random pattern in both FrMSB and BrWSB tumors, suggesting that the *Rad51b* gene underwent a loss-of-function mutation in our SB mouse models. We then performed IHC staining to assess RAD51B protein expression levels in BrWSB and FrMSB tumors (Fig. S2B). The expression level of RAD51B was lower in TNBC tumors (Fig. S2C, D) and especially, positively correlated with ERα protein levels in both FrMSB and BrWSB tumors (Fig. S2E–H). These data indicate that SB-mediated mutagenesis of *Rad51b* facilitates tumorigenesis and triggers TNBC formation during tumor progression. Next, we evaluated RAD51B protein expression levels in different cell lines. The results showed that RAD51B expression in human or mouse TNBC cells was relatively lower than in other subtypes of breast cancer cell lines (Fig. 1F). Bioinformatic analysis of The Cancer Genome Atlas database showed no significant differences in *Rad51b* mRNA levels between molecular subtypes of breast cancer (Fig. S2I). However, 50% (10/20) of *Rad51b* loss-of-function somatic mutation patients were diagnosed with basal-like breast cancer, which is significantly increased a lot when compared to non-carriers (Fig. 1G). Basal-like tumors are often referred to as TNBC because the majority of them are typically negative for ERα, PR and HER2 [34, 35]. Kaplan–Meier plot of patients with basal-like breast cancer also showed that patients with lower levels of RAD51B have a worse probability of survival compared to patients with higher levels of RAD51B (Fig. 1H). These clinical data are consistent with our finding that *Rad51b* loss-of-function mutation accelerates TNBC formation. Therefore, we chose *Rad51b* as our candidate gene for further evaluation.

### RAD51B is required for ERα expression during tumorigenesis

**Fig. 2:**
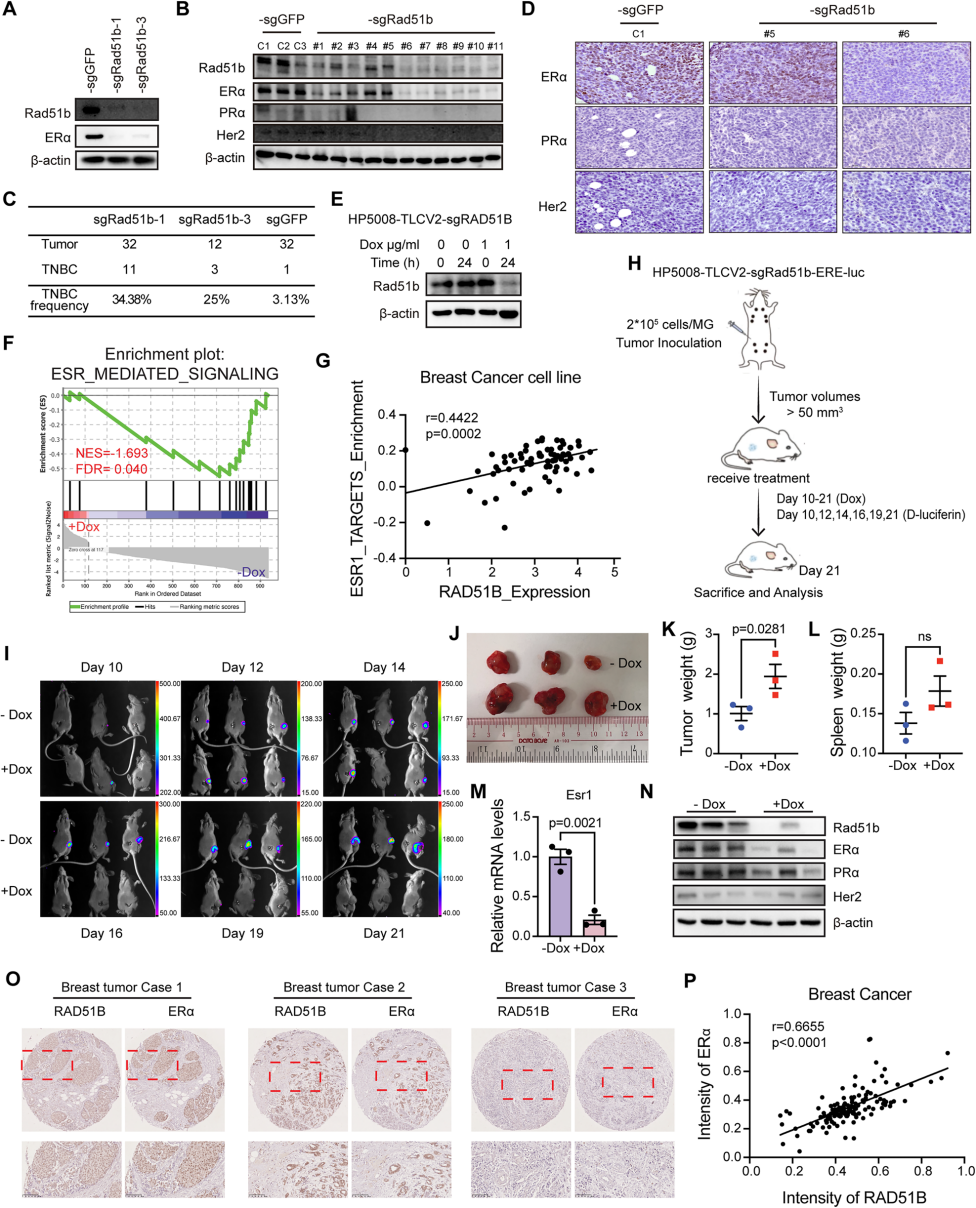
RAD51B is required for ERα expression during tumorigenesis. **A** The knocking-out efficiency of two independent sgRNA in HP5008 cells and the relative expression of ERα protein, as measured by western blotting. **B** Whole tissue lysates from different tumors in WT or *Rad51b* knockout group were subjected to western blot analysis. **C** Table summary the TNBC frequency among different groups. **D** Representative IHC staining images of ERα, PR and Her2 from each group. Scale bar, 50 μm. **E** The knocking-out efficiency of dox-induced RAD51B knockout in HP5008 cells and relative expression of RAD51B protein, as measured by western blotting. **F** GSEA analysis reveals that downregulated genes in *Rad51b* knockout cells are enriched in ESR_MEDIATED_SIGNALING gene set. **G** Scatter plot showing the positive correlation between *Rad51b* mRNA expression and ESR1_TARGETS_Enrichment score in breast cancer cell lines. Pearson’s correlation coefficient *r* and two-tailed *P* value were shown. **H** The strategy for establishing mouse models for in vivo ERα signaling detection. **I** Representative images of bioluminescence signals of tumor-bearing mice treated with or without doxycycline (dox) to induce *Rad51b* knockout (*n* = 3 mice per group). **J**–**L** Tumor images (**J**), tumor weight (**K**) and spleen weight (**L**) of each group on day 21. **M**, **N** The expression of ERα in different tumors was measured by RT-qPCR (**M**) and western blotting (**N**). Data are presented as the means ± SEM, with statistical significance among groups determined by Student’s *t* test. **O**–**P** Representative images of IHC staining (**O**) and correlation **P** between RAD51B and ERα abundance in human breast cancer tissues. Scale bar, 100 μm. Each data point represents the value from an individual patient (*n* = 136). Pearson’s correlation coefficient *r* and two-tailed *P* value were shown.

For the functional validation, we used HP5008 cells, which are mammary tumor cells derived from *Fgfr2-S252W;MMTV-Cre* mice with a single spontaneously arising mammary tumor at the age ~12 months [25], showed positive for ERα but negative for PR and HER2 expression in all tumor samples when injected into nude mice (Fig. S3). We knocked out *Rad51b* in HP5008 cells, and the knockout efficiency is shown in Fig. 2A. *Rad51b* deletion significantly enhanced the colony formation capacity and increased cell growth in HP5008 cells (Fig. S4A, B). To validate the function of RAD51B in vivo, HP5008 cells stably transduced with sgRNAs targeting *Rad51b* or non-targeting control were inoculated into the fat pads of nude mice, respectively. We found that RAD51B deletion increased the tumor growth in mice (Fig. S4C). Moreover, RAD51B deletion enhanced the capacity of breast cancer cells to form secondary tumors in immunodeficient mice (Fig. S4D–F). The expression levels of ERα were downregulated in *Rad51b* knockout cells (Fig. 2A). We then injected cells into the mammary fat pad and collected tumors from the experimental group and the control group. *Rad51b* knockout significantly decreased the protein expression levels of ERα (Fig. 2B) and increased the percentage of tumors with TNBC subtype compared to the non-target control (Fig. 2C, D). To investigate RAD51B function, we generated the doxycycline (dox)-inducible *Rad51b* knockout cells. Western blot analysis confirmed efficient depletion of RAD51B protein following 24 h treatment with 1 µg/ml dox (Fig. 2E). We thus conducted gene expression analysis of dox-induced *Rad51b* knockout cells compared with untreated cells. GSEA revealed that ESR1-mediated signaling genes were downregulated after dox-induced *Rad51b* knockout (Fig. 2F). Indeed, RT-qPCR analysis confirmed that loss of RAD51B expression decreased *Esr1* and its downstream genes expression, including *Egfr*, *Hbegf* and Areg (Fig. S4G). Additionally, we observed a significant positive correlation between *RAD51B* expression and *ESR1* downstream target enrichment in human breast cancer cell lines (Fig. 2G). This positive correlation was also observed in 1515 human cancer cell lines across 30 tissue types (Fig. S4H), suggesting a regulatory relationship between RAD51B and the estrogen-response pathway. We then established an in vivo reporter system, which allows the evaluation of ER signaling in breast tumors that are treated with or without dox to knockout *Rad51b*. Inducible cells with ERE-luciferase reporter were injected into mice with mammary fat pad implantation. After tumor formation was noticed, we treated the mice with dox in drinking water daily and injected D-luciferin to evaluate the luciferase signal on the indicated days (Fig. 2H). We observed enhanced tumor growth (Fig. 2I–L and Fig. S4I) but decreased luciferase signal after dox-induced *Rad51b* knockout at day 16 compared with control (Fig. 2I). Further RT-qPCR and western blot analysis confirmed that *Esr1* mRNA and ERα protein expression levels were downregulated (Fig. 2M, N). According to IHC staining results, tumors from the *Rad51b* knockout group were negative for ERα, while the control group was positive (Fig. S4J). Inducible 545 cells, isolated from a TNBC tumor of a *Brca1*-MSK mouse, were injected into mice with mammary fat pad implantation [10]. Mice were treated with dox in drinking water from day 9 to day 42, and tumor samples were collected on the indicated day (Fig. S4K). Similar to previous findings, tumors grow faster in the dox-treated group than in the control group (Fig. S4L). Surprisingly, we found that tumors in the control group were also ERα-positive at the early stage but gradually became negative, and *Rad51b* knockout accelerated the switch from ERα-positive to ERα-negative subtype (Fig. S4M). These data indicate that TNBC tumors can arise from positive cells through various factors, and RAD51B serves as a regulator affecting ERα status during tumorigenesis. We therefore examined the levels of RAD51B and ERα in tissue microarrays from 136 breast cancer patient samples. A positive correlation between RAD51B and ERα protein expression was observed in human breast cancer (Fig. 2O, P). Altogether, these data demonstrate that RAD51B is essential for maintaining ERα-positive cell identity and might contribute to the switch of lineage commitment in breast cancer cells.

### RAD51B deletion induces trimethylation of histone H3K27 on *Esr1* promoter region

**Fig. 3:**
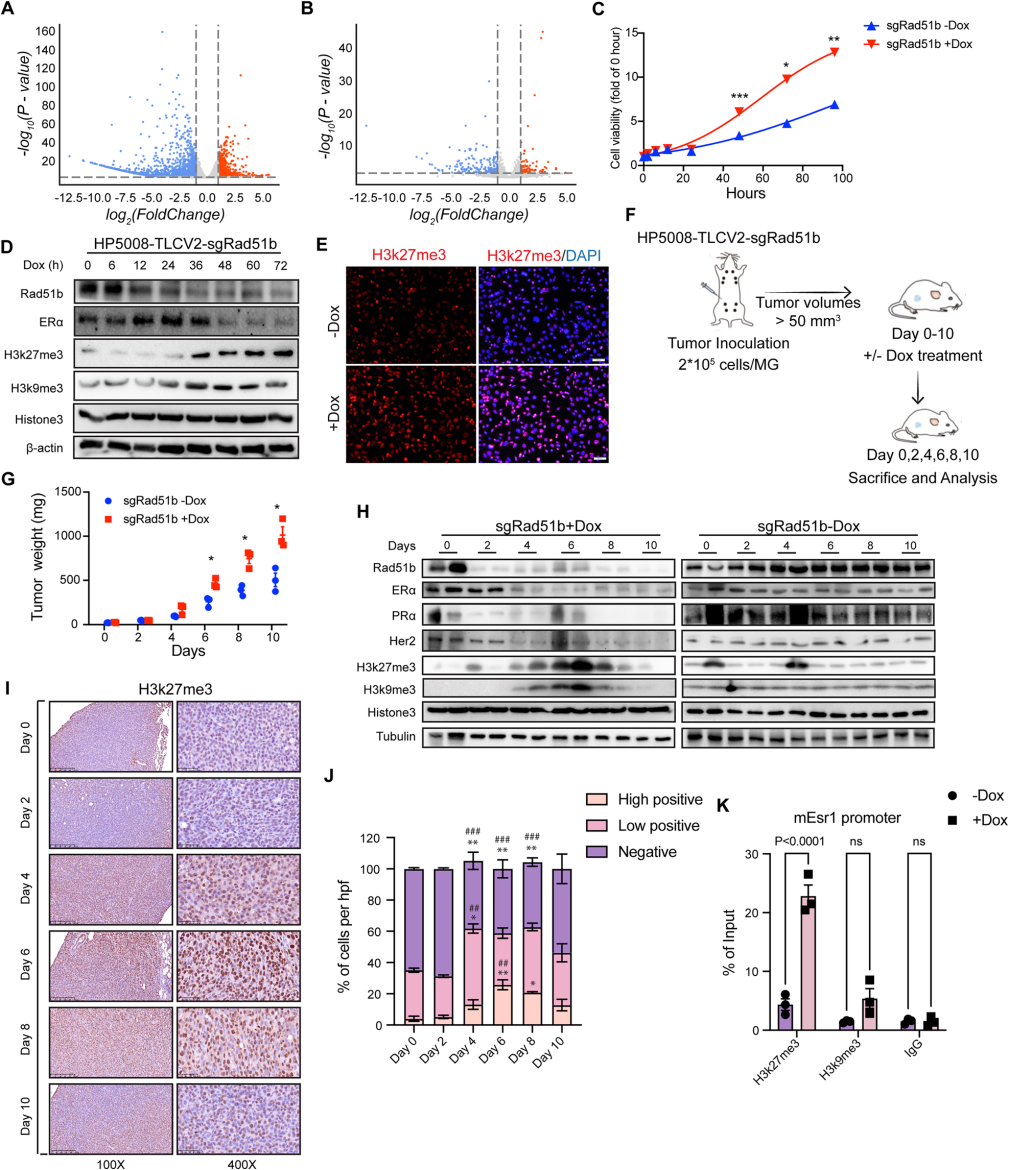
RAD51B deletion induces trimethylation of histone H3 lysine 27 on *Esr1* promoter region. **A**, **B** RNA-seq analysis revealed that the majority DEGs were downregulated following *Rad51b* knockout in vitro (**A**) and in vivo (**B**). **C** Cell proliferation of HP5008 cells treated with dox to induce *Rad51b* knockout. **D**, **E** Increased trimethylation of H3K27 following dox-induced *Rad51b* knockout was measured by western blotting (**D**) and immunofluorescence staining (**E**). **F** The strategy for establishing a dox-induced Rad51b knockout mouse model. **G** Tumor weight of tumors from each group collected at the indicated time point (*n* = 3 per group). Data are presented as the means ± SEM, with statistical significance among groups determined by a two-way ANOVA test comparing tumor volumes between each group on each day separately. **H** Representative protein expressions were measured by western blotting. **I**, **J** H3K27me3 levels of tumor samples from the indicated group were measured by immunohistochemistry staining (**I**) and quantified results (**J**). Data are presented as the means ± SEM with statistical significance among groups determined by Dunnett’s multiple-comparisons test. *P < 0.05, **P < 0.01, ***P < 0.001 vs. the signal-matched Day 0 group; ^##^*P* < 0.01, ^###^*P* < 0.001 vs. the signal-matched Day 2 group. **K** ChIP-qPCR assays using antibody anti-H3K9me3, -H3K27me3, and IgG as a negative control. Data are presented as the means ± SEM, with statistical significance among groups determined by a two-tailed Student’s *t* test.

Previous findings led us to investigate the mechanisms by which the loss of RAD51B in ERα-positive cells restrains ERα signaling. We carried out RNA-seq experiments on dox-induced *Rad51b* knockout cells and tumors, comparing them with untreated controls. Following the loss of expression of RAD51B, the majority of differentially expressed genes (DEGs) were downregulated (Fig. 3A and B). This prompted us to explore whether epigenetic modifications were involved, and which ones played a crucial role in inhibiting ERα expression upon RAD51B loss. Dox-induced *Rad51b* knockout increased cell proliferation compared to the untreated group (Fig. 3C). We then treated the cells with dox and collected samples at different time points for Western blot and immunofluorescence (IF) staining analysis. Following the onset of RAD51B downregulation at 12 h post-dox treatment, ERα expression decreased by 48 h, while the trimethylation of histone H3 lysine 27 (H3K27me3), a transcriptionally repressive epigenetic mark, was strongly enhanced by 36 h (Fig. 3D, E). In addition to H3K27me3, we also analyzed other epigenetic marks, including H3K9me3, H3K9ac and H3K4me3. Notably, dox-induced RAD51B knockout enhanced H3K9me3 modification (Fig. 3D). These results suggest that RAD51B deletion modulates gene expression patterns through enhancing H3K27me3 and H3K9me3 modifications. Next, we performed mammary fat pad injections to establish allograft tumor models (Fig. 3F). Mice received dox treatment when the tumors reached ~50 mm^3^, and tumor samples were collected every two days for western blotting and IHC staining analysis. Dox-induced *Rad51b* deletion accelerated tumor growth compared to the untreated control (Fig. 3G). Moreover, dox-induced *Rad51b* deletion decreased ERα expression (Figs. 3H and S5A) by day 4, while the H3K27me3 (Fig. 3H–J) and H3K9me3 (Fig. S5B, C) increased significantly, providing a hint that the loss of RAD51B expression may restrain ERα signaling through a repressive histone modification pathway. However, their respective roles in regulating ERα expression remained unclear. To address this, we performed chromatin immunoprecipitation (ChIP)-qPCR assays on dox-treated and -untreated cells to quantify H3K27me3 and H3K9me3 enrichment at the ERα promoter region, identifying which modification mediates ERα suppression following RAD51B depletion. The results showed a significant increase in H3K27me3 on the ERα promoter after *Rad51b* knockout, while another repressive epigenetic mark, H3K9me3, showed slightly increased (Fig. 3K). These results indicate that the loss of RAD51B expression initiates an epigenetic modification process to regulate downstream gene expression, including enhancing H3K27 trimethylation and repressing ERα expression, leading to a shift from ERα-positive to ERα-negative.

### RAD51B deletion increases recruitment of PRC2 to *Esr1* promoter region

**Fig. 4:**
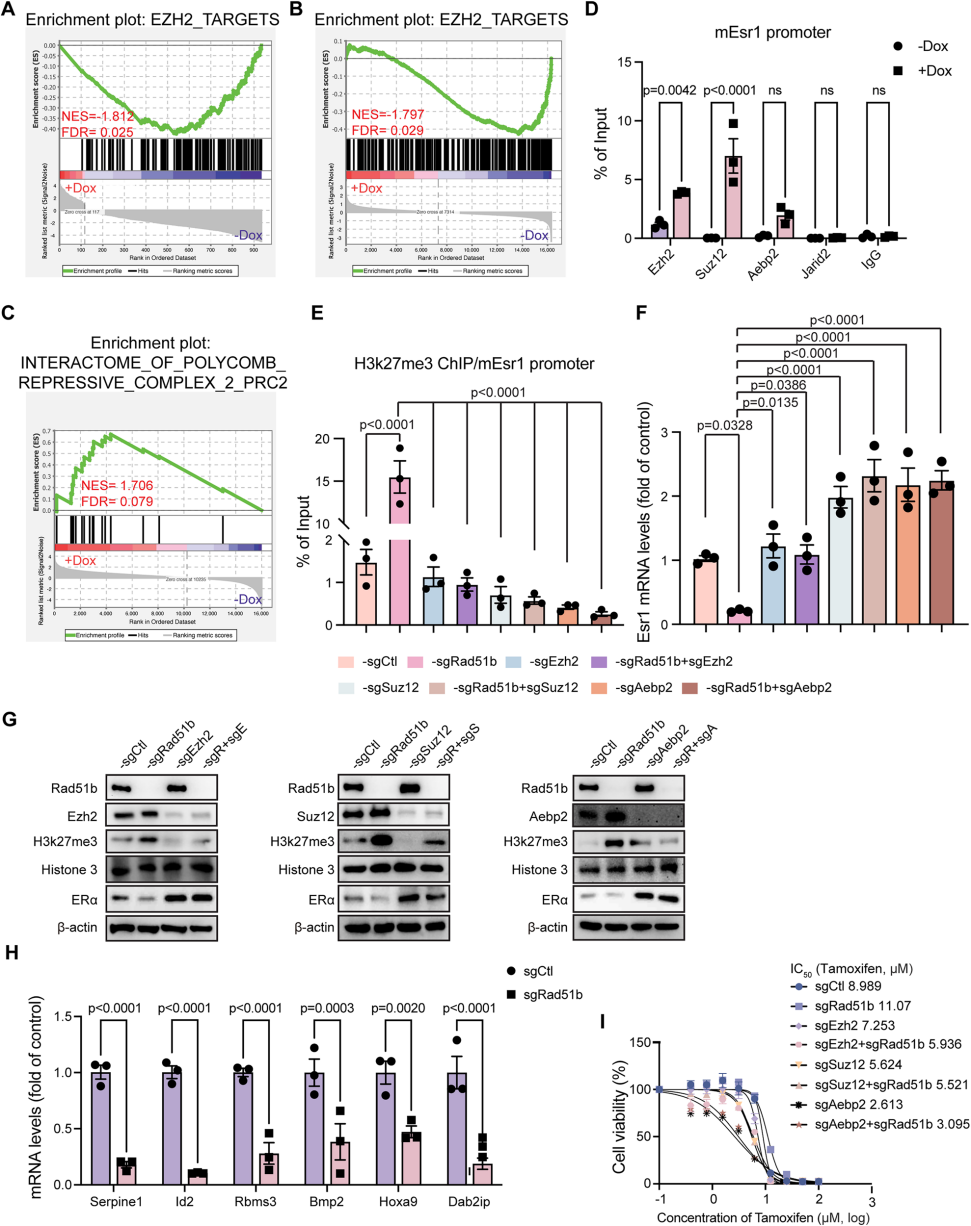
RAD51B deletion induces the recruitment of PRC2 to *Esr1* promoter region. **A**, **B** GSEA analysis reveals that downregulated genes in Rad51b knockout cells (**A**) and tumors (**B**) are enriched in the EZH2_TARGETS_UP term. **C** GSEA of significant gene sets enriched in HP5008 cells with *Rad51b* knockout. **D** ChIP-qPCR assays using antibody anti-EZH2, -SUZ12, -AEBP2, -JARID2 and IgG as a negative control to detect binding of EZH2, SUZ12, AEBP2 and JARID2 to ERα promoter regions. Data are presented as the means ± SEM with statistical significance among groups determined by a two-tailed Student’s *t* test. **E** ChIP-qPCR analysis of the enrichment of H3K27me3 at the promoter region of *ESR1*. **F** *ESR1* expression levels in each group were measured by RT-qPCR. **E**, **F** Data are presented as the means ± SEM with statistical significance among groups determined by one-way ANOVA. **G** Western blotting showing the expression of the indicated protein after knocking out *Rad51b*, *Ezh2*, *Suz12* or *Aebp2* in HP5008 cells. **H** The mRNA expression of PRC2 downstream genes in HP5008 cells with or without dox-induced *Rad51b* knockout. Data are presented as the means ± SEM with statistical significance among groups determined by a two-tailed Student’s *t* test. **I** Tamoxifen effects on cell viability with indicated cell lines for 48 h treatment. IC_50_ values for tamoxifen are indicated. Data are presented as means ± SEM of three independent experiments.

PRC2 is a chromatin-modifying enzyme that catalyzes the methylation of histone H3 at lysine 27 (H3K27me1/2/3) [36]. The PRC2 complex has four core subunits: the catalytic SET domain-containing subunit Enhancer of zeste homolog 2 (EZH2), embryonic ectoderm development (EED), suppressor of zeste 12 (SUZ12) and retinoblastoma (Rb)-associated proteins 46/48. Additionally, the PRC2 complex includes several cofactors, such as AEBP2 and JARID2, which modulate its recruitment and activity [37–39]. GSEA analysis showed that the enrichment of EZH2 target genes was downregulated in both cells and tumors with *Rad51b* knockout (Fig. 4A, B). GSEA analysis also revealed an enrichment of Interactome_of_polycomb_repressive_complex_2_(PRC2) signature in cells with *Rad51b* knockout (Fig. 4C). We examined whether the PRC2 complex was involved in regulating ERα expression in *Rad51b* knockout cells. However, the mRNA and protein levels of PRC2 complex members did not show obvious differences after dox-induced *Rad51b* knockout (Fig. S6A, B). We thus evaluated the local chromatin enrichment of the PRC2 complex on the ERα promoter region using ChIP-qPCR assay. The results showed a slight increase in AEBP2 recruitment but a significant increase in EZH2 and SUZ12 recruitment to the ERα promoter when *Rad51b* was knocked out (Fig. 4D). We then used CRISPR-Cas9 to double-knockout *Rad51b* with *Ezh2*, *Suz12* or *Aebp2* one by one. Since *Rad51b* knockout increased H3K27 trimethylation on the promoter region of ERα, further knockout of *Ezh2*, *Suz12* or *Aebp2* reversed H3K27 trimethylation on the ERα promoter region (Fig. 4E), leading to de-repression of ERα gene expression. Therefore, we performed an RT-qPCR assay to examine the mRNA levels of *Esr1* in these cell samples. Indeed, further knockout of PRC2 complex members abolished the repressive effects of *Rad51b* knockout and reversed *Esr1* gene expression (Fig. 4F). Western blot assay also showed reversed ERα protein expression in double knockout cells compared to that in *Rad51b*-deficient cells (Fig. 4G). We also evaluated the mRNA expression levels of PRC2 complex downstream genes through RT-qPCR assay. Cells with dox-induced *Rad51b* knockout showed decreased expression of PRC2 complex downstream genes (Fig. 4H), suggesting that *Rad51b* knockout triggers the activation of PRC2 complex to initiate an epigenetic modification process. Besides, we also investigated whether *Rad51b* knockout triggers the activation of PRC2 complex to affect PR or Her2 expression. ChIP-qPCR and RT-qPCR experiments showed a moderate effect on H3K27 trimethylation on the PRα promoter region and *Pgr* mRNA expression, but no significant effect of *Erbb2/Neu* expression (Fig. S6C–F). After confirming that double knockout cells effectively reversed ERα protein expression, we proceeded with tamoxifen treatment to assess its cytotoxic effects across different groups. Cells with *Rad51b* knockout exhibited reduced sensitivity to tamoxifen, whereas those with double knockout displayed reversed response (Fig. 4I).

To exclude the system interference in HP5008 cells, we conducted CRISPR-Cas9 knockouts of *Rad51b*, *Ezh2*, *Suz12* and *Aebp2* in *Brca1*-mutant 545 cells, which were isolated from a primary mammary tumor of a *Brca1*-MSK mouse. Western blot and Alamar blue assay showed similar results to those in the HP5008 system (Fig. S6G, H). Moreover, in human breast cancer MCF-7 cells, RAD51B deletion downregulated ERα expression and enhanced tamoxifen sensitivity. However, additional knockout of *Ezh2*, *Suz12* or *Aebp2* successfully reversed these effects (Fig. S6I, J), indicating that RAD51B deficiency suppresses ERα expression by enhancing PRC2 complex recruitment regardless of the cell types. Together, these data show that RAD51B deficiency enhances the recruitment of PRC2 complex to the ERα promoter region, catalyzing the trimethylation of histone H3 at lysine 27 and resulting in decreased ERα expression.

### RAD51B deletion leads to reduced phosphorylation of EZH2 at Thr311 through AMPK pathway

**Fig. 5:**
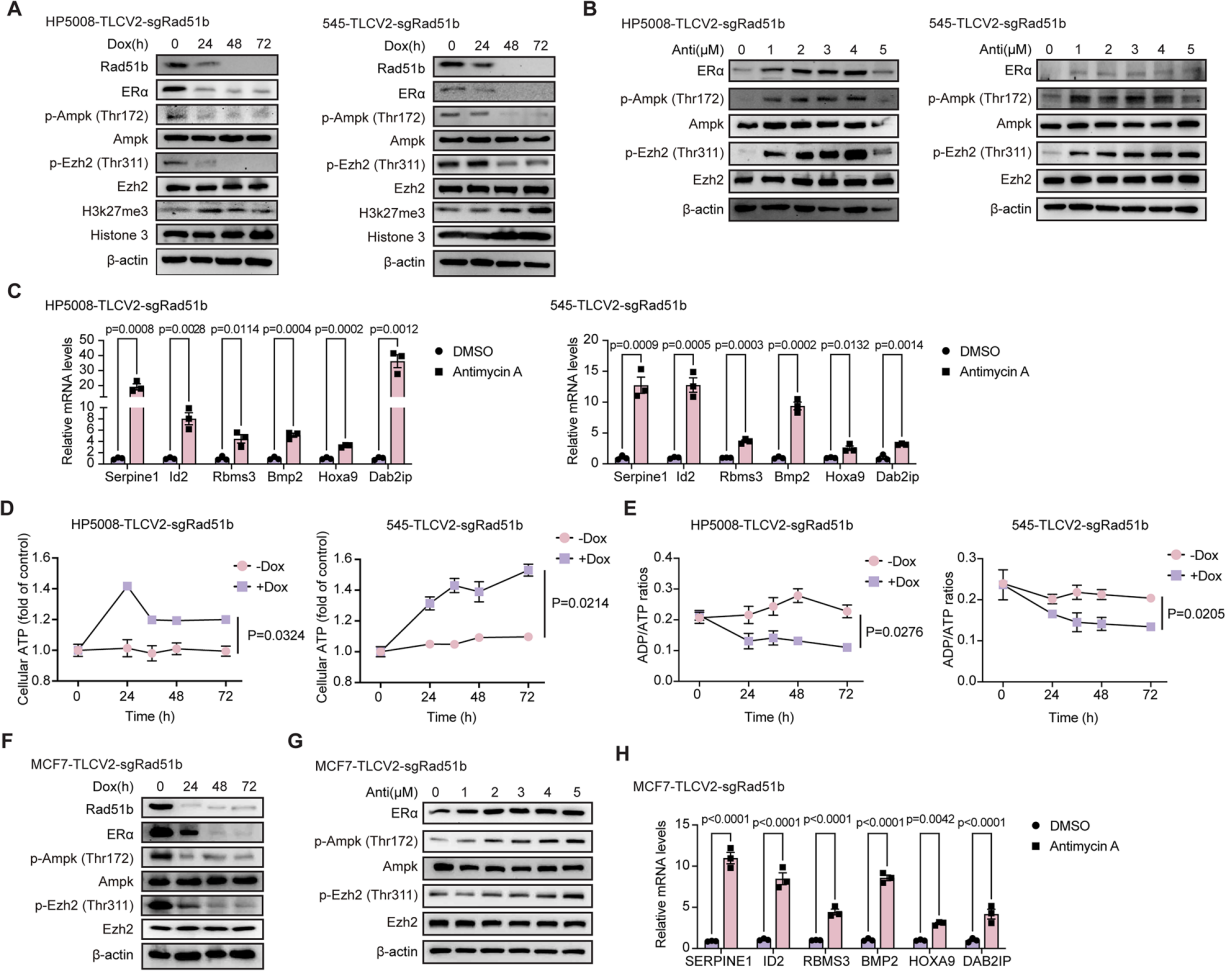
RAD51B deletion leads to reduced phosphorylation of EZH2 at Thr311 through AMPK pathway. **A** Western blot showing the expression of the indicated protein after dox-inducing *Rad51b* knockout in HP5008 and 545 cells. **B** Western blot showing the expression of the indicated protein after cells were treated with antimycin A at different concentrations. **C** The mRNA expression of PRC2 downstream genes in HP5008 and 545 cells with or without antimycin A treatment. Data are presented as the means ± SEM with statistical significance among groups determined by a two-tailed Student’s *t* test. **D** Cellular ATP levels in HP5008 and 545 cells with or without dox-induced *Rad51b* knockout. **E** Cellular ADP/ATP ratios in HP5008 and 545 cells with or without dox-induced *Rad51b* knockout. **D**, **E** Data are presented as the means ± SEM of three independent experiments, with statistical significance among groups determined by a two-tailed Student’s *t* test. **F** Western blotting showing the expression of the indicated protein after dox-inducing *Rad51b* knockout in MCF-7 cells. **G** Western blotting showing the expression of the indicated protein after MCF-7-TLCV2-sgRad51b cells treated with antimycin A in different concentrations. **H** The mRNA expression of PRC2 downstream genes in MCF-7-TLCV2-sgRad51b cells with or without antimycin A treatment. Data are presented as the means ± SEM with statistical significance among groups determined by a two-tailed Student’s *t* test.

We next sought to understand how RAD51B deletion affects the recruitment of PRC2 to *Esr1* promoter region. Recent studies have demonstrated that post-translational modifications of EZH2 are crucial for its enzymatic activity, leading to diverse biological functions [40–43]. Since the mRNA and protein levels of the main components of PRC2 did not show obvious differences after dox-induced *Rad51b* knockout (Fig. S6A, B), this suggests that RAD51B deletion might not affect the transcription and translation processes. We then investigated whether the post-translational modification events are involved. We examined EZH2 phosphorylation at three functionally important sites, including Thr311, Thr345 and Thr416, all of which have been previously reported to regulate EZH2 catalytic activity [40–44]. Surprisingly, we found that the phosphorylation of EZH2 at Thr311 is specifically reduced when cells lose RAD51B expression (Fig. 5A), while the phosphorylation of EZH2 at Thr345 or Thr416 remains unchanged (Fig. S6B). Phosphorylation of EZH2 at Thr311 by AMPK suppresses PRC2 methyltransferase activity by disrupting the interaction between EZH2 and SUZ12 [44]. Decreased phosphorylation of AMPK after *Rad51b* knockout was further confirmed by western blotting (Fig. 5A). We treated cells with antimycin A to activate AMPK kinase, which enhanced the phosphorylation of EZH2 at Thr311 and subsequently upregulated the expression of ERα protein (Fig. 5B). Cells that were treated with antimycin A showed enhanced expression of PRC2 complex downstream genes (Fig. 5C). AMPK is a kinase that senses energy levels by detecting augmentations in the ADP/ATP ratio [45]. We measured the changes in cellular ATP levels after dox-induced *Rad51b* knockout in both HP5008 and 545 cells. Indeed, under conditions of equal cell numbers, increased cellular ATP (Fig. 5D) and decreased ADP/ATP ratios (Fig. 5E) were confirmed in dox-induced *Rad51b* knockout cells. This suggests that *Rad51b* knockout affects cellular ATP levels, suppressing AMPK activation, which in turn inhibits the phosphorylation of EZH2 at the Thr311 region. The decrease in phosphorylation at the Thr311 region of the EZH2 protein enhances the activities of the PRC2 complex and represses downstream factors, including ERα. In human breast cancer MCF-7 cells, dox-induced *RAD51B* knockout reduced AMPK phosphorylation and EZH2 phosphorylation at Thr311, leading to decreased ERα expression (Fig. 5F). However, treatment of MCF-7 cells with antimycin A, which activates AMPK kinase (Fig. 5G) and enhances expression of PRC2 complex downstream genes (Fig. 5H), resulted in only a slight upregulation of ERα expression (Fig. 5G). This observation could be explained by the low recruitment of EZH2 at the *ESR1* promoter region in MCF-7 cells. Therefore, enhanced phosphorylation of EZH2 at Thr311, which inhibits EZH2 activity, had minimal effect on upregulating ERα expression.

### Inhibition of EZH2 activity reverses ERα expression and sensitizes tumors to endocrine therapy

**Fig. 6:**
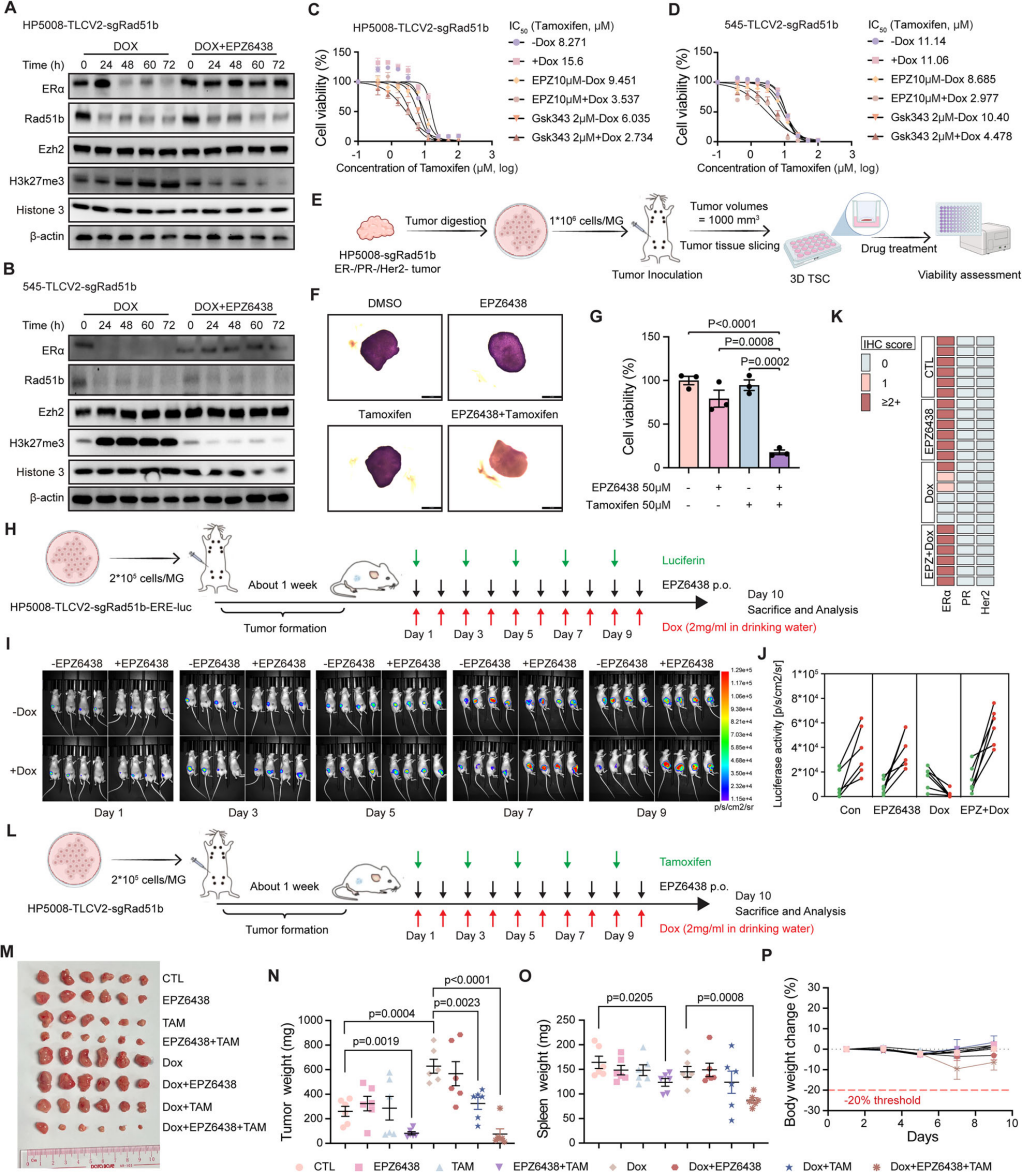
Inhibition of EZH2 activity reverses ERα expression and sensitizes tumors to endocrine therapy. **A**, **B** Western blotting showing the expression of the indicated protein after different treatment in HP5008 cells (**A**) and 545 cells (**B**). **C**, **D** Effect of EZH2 inhibition on tamoxifen sensitivity in HP5008 (**C**) and 545 (**D**) cells with or without dox-induced *Rad51b* knockout. IC_50_ values for tamoxifen are indicated. Data are presented as means ± SEM of three independent experiments. **E** The strategy for establishing tumor slice culture system to investigate the effects of EZH2 inhibitor together with tamoxifen in *Rad51b*-knockout TNBC tumor. **F** Visual antitumor response in tumor slice culture system after 1 week treatment revealed by MTT analysis and representative quantified results (**G**). Data are presented as the means ± SEM, with statistical significance among groups determined by a one-way ANOVA test. **H** The strategy for establishing mouse models for in vivo ERα signaling detection. **I** Representative images and quantitation analysis (**J**) of bioluminescence signals from Day 1–9 of tumor-bearing mice treated with or without dox to induce *Rad51b* knockout and EZH2 inhibitors treatment. **K** Summary of ERα, PR and Her2 abundance in tumors from each group measured by IHC analysis. **L** The strategy for establishing mouse models for in vivo investigation of the effects of combination therapy. Tumor images (**M**), tumor weight (**N**), spleen weight (**O**), and body weight change (**P**) of mice in each group. Data are presented as the means ± SEM with statistical significance among groups determined by a one-way ANOVA test.

EPZ6438 (Tazemetostat) is a potent and selective SAM-competitive EZH2 inhibitor (EZH2i), approved by the FDA to treat patients with metastatic or locally advanced epithelioid sarcoma not eligible for complete resection [46]. GSK343 is another selective SAM-competitive EZH2 inhibitor that prevents the trimethylation of H3K27 [47]. We first tested the inhibitory effects of these two drugs on HP5008 and 545 cells with or without dox-induced *Rad51b* knockout (Fig. S7A, B). We then selected low doses that would not affect the cell viability to investigate the influences of these EZH2 inhibitors on ERα expression in *Rad51b*-deficient background. Since cells that were exposed to dox induced *Rad51b* knockout showed increased trimethylation of histone H3 lysine 27 and decreased ERα expression. Treatment with low doses of EPZ6438 or GSK343 reversed these effects (Figs. 6A, B and S7C), demonstrating that pharmacological inhibition of EZH2 effectively de-represses ERα expression in *Rad51b*-deficient cells. We then asked whether the inhibition of EZH2-induced ERα protein is functional and renders cells ERα-dependent. In vitro assays revealed synergistic effects of the combination treatment of EZH2i with tamoxifen in *Rad51b*-deficient cells (Fig. 6C, D). We also established a 3D tumor slice culture (3D-TSC) system, which can mimic the complex microenvironment and heterogeneity of tumors, to measure the therapeutic efficacy of our drugs treatment [48] (Fig. 6E). EZH2i or tamoxifen monotherapy showed limited killing effects compared with the vehicle control, while the combination of EZH2i with tamoxifen showed strong synergistic killing effects on tumor slices (Fig. 6F, G). These results suggest that combination therapy of EZH2i with tamoxifen may also be effective in vivo. To further prove this, HP5008-TLCV2-sgRad51b cells were transfected with ERE-luciferase reporter and injected into the mice with mammary fat pad implantation (Fig. 6H). After tumor formation, mice were treated with EPZ6438 and doxycycline on the indicated days, and luciferase signals were detected every 2 days to reflect ERα status during tumor growth. As we previously showed that *Rad51b* knockout decreased luciferase signaling and ERα expression during tumor progression, combination treatment with low-dose EZH2i (20 mg/kg) efficiently blocked the effects caused by RAD51B deficiency (Fig. 6l–K and S7D), indicating that inhibiting the EZH2 pathway can effectively maintain ERα-mediated signals even when tumors lose RAD51B expression. For combination therapy, mice were administered tamoxifen, EPZ6438 and doxycycline according to Fig. 6L. Tumors in the ox-induced *Rad51b* knockout group grew much faster than wild-type tumors. Tamoxifen monotherapy resulted in marked inhibitory effects on tumor growth compared with vehicle control, and the addition of EZH2i nearly completely inhibited tumor growth, as determined by tumor weight measurement (Fig. 6M–O). To monitor the toxic effects of the combination therapy, we measured body weight throughout the treatment period and performed H&E staining of major organs collected at the end of the therapy. The majority of mice maintained a normal body weight throughout the treatment (Fig. 6P). The H&E staining assay also indicated no obvious hydropic damage or necrotic lesions, validating satisfactory biocompatibility of the therapies (Fig. S7E). These results indicate that selectively blocking EZH2 catalytic activities using an inhibitor can effectively de-repress ERα and sensitize *Rad51b*-deficient cells or tumors to endocrine therapy.

### Targeting EZH2 confers the anti-estrogen sensitivity of ERα-negative breast cancer

As the above results suggest, pharmacological inhibition of the EZH2 pathway effectively re-expresses ERα protein in *Rad51b*-deficient cells or tumors, sensitizing them to endocrine therapy. We wondered if EZH2i treatment re-expresses ERα protein in TNBC and enhances the curative effects of endocrine therapy. We first treated various human and mouse TNBC cell lines with EZH2i and measured the expression levels of ERα protein. TNBC cells treated with EZH2i reactivated ERα expression in a dose- and time-dependent manner (Fig. S8A). Although no obvious enhancement of ERα expression was detected within 12 h of treatment, we still observed an increase in ERα downstream signaling using the ERE-dscGFP reporter assay (Fig. S8B, C). We then evaluated whether EZH2i treatment rendered TNBC cells sensitive to endocrine therapy. Indeed, a low dose of EZH2i enhanced the inhibitory effects of tamoxifen in various TNBC cells (Fig. S8D). Together, inhibition of EZH2 activities induced ERα expression and activated its downstream signaling in different TNBC cells, leading to enhanced sensitivity to endocrine therapy.

**Fig. 7:**
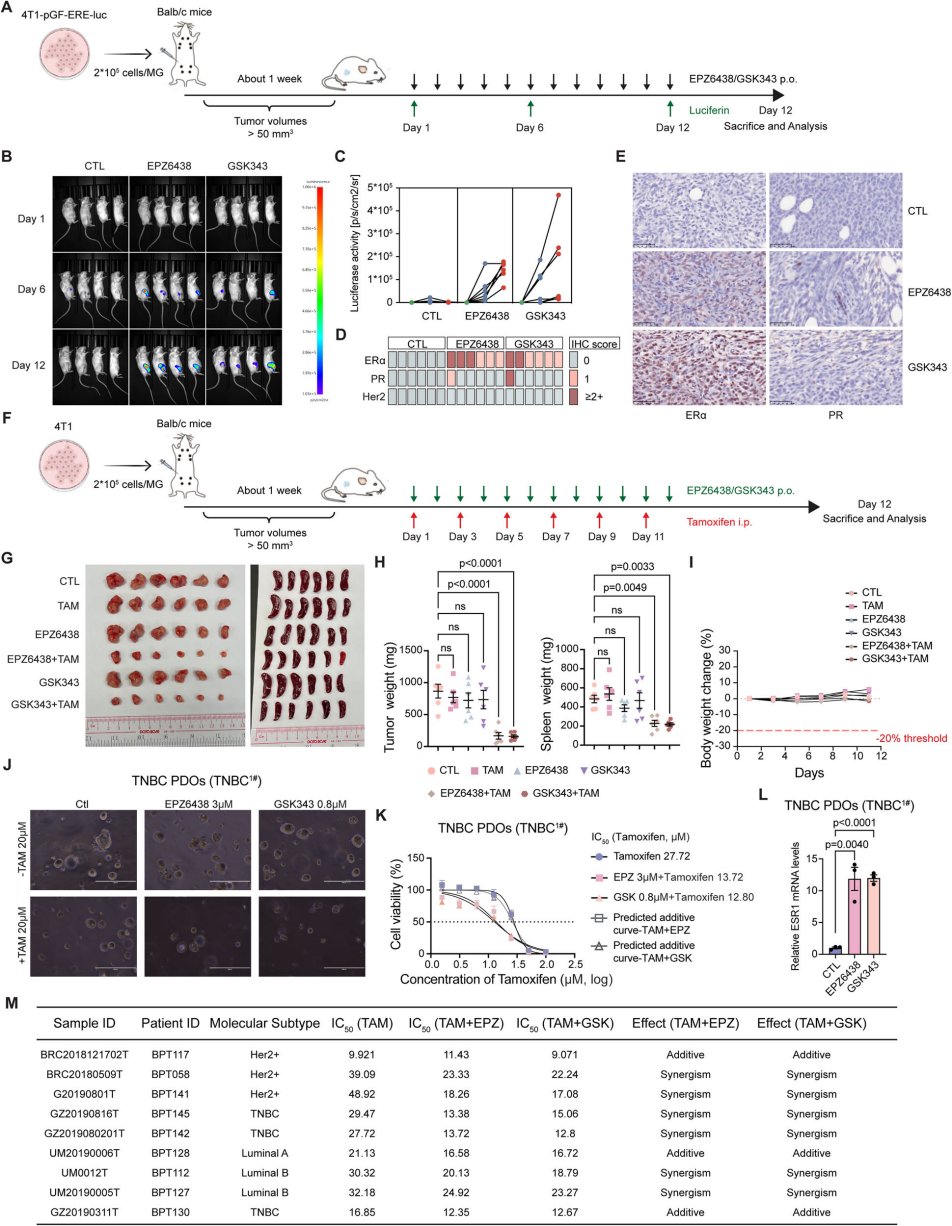
Targeting EZH2 confers anti-estrogen sensitivity on ERα-negative breast cancer. **A** The strategy for establishing mouse models for in vivo ERα signaling detection. **B** Representative images and quantitation analysis (**C**) of bioluminescence signals of tumor-bearing mice. **D** Summary of ERα, PR, and Her2 abundance in tumors from each group measured by IHC analysis. **E** Representative images of ERα and PR abundance in tumors from each group measured by IHC analysis. **F** The strategy for establishing mouse models for in vivo investigation of the effects of combination therapy. Tumor images (**G**), tumor weight, spleen weight (**H**) and body weight change (**I**) of mice in each group. Data are presented as the means ± SEM, with statistical significance among groups determined by a one-way ANOVA test. **J**, **K** Representative images (**J**) and quantification (**K**) of cell viability of patient-derived organoids of triple-negative breast cancer treated with DMSO, EPZ6438, GSK343 and tamoxifen (TAM) for 4 days. Scale bar, 200 μm. IC_50_ values for tamoxifen are indicated. Data are presented as means ± SEM of three independent experiments. **L** *ESR1* expression levels in each group were measured by RT-qPCR. Data are presented as the means ± SEM, with statistical significance among groups determined by a one-way ANOVA test. **M** Summary the information of different PDOs and IC_50_ values for TAM treatment in different experimental groups.

To determine whether the combination therapy of EZH2i and tamoxifen is effective in vivo, 4T1 cells with ERE-luciferase reporter were implanted into the mammary fat pad of BALB/c mice. Once the tumors were established, the mice were treated with either EZH2i or vehicle (Fig. 7A). To measure ERα status during tumor progression, luciferase activities were detected every five days using an in vivo imaging system. Interestingly, a very weak luciferase signal was detected during early progression but decreased later in the control group, consistent with our previous finding that early-stage triple-negative tumors also require ERα for growth. In the EZH2i-treated mice, luciferase signals gradually increased throughout the entire period of tumor progression (Fig. 7B, C). IHC staining was performed to identify the molecular subtypes of tumors collected at the end of treatment. Tumors from control mice were negative for ERα, PR and Her2 expression, while 33–50% of EZH2i-treated tumors showed positive ERα expression (Fig. 7D, E). Within the ERα-negative tumors from EZH2i-treated groups, we also noticed some cells expressed ERα protein, which was not observed in the control tumors. Additionally, we observed enhanced PR protein expression in some EZH2i-treated tumors. We then conducted combination treatment using low doses of EZH2i together with tamoxifen, as shown in Fig. 7F. Our results indicated that single-drug treatment had a limited inhibitory effect, while drug combinations were more efficient in inhibiting tumor growth (Fig. 7G, H). During the entire period of treatment, there were no significant changes in the body weight of mice in all groups (Fig. 7I). Histopathological examination also showed that the major organs of mice in all groups were regularly arranged and well defined (Fig. S9A). These results indicate that EZH2-targeted therapy reactivates ERα expression and that ERα protein serves as a functional target, rendering TNBC tumors sensitive to endocrine therapy. We also repeated these in vivo examinations using the EMT6 cell line, another murine mammary carcinoma cell line that is triple-negative for ERα, PR and Her2 expression. The results showed a similar phenomenon to that observed in the 4T1 model (Fig. S9B–K), demonstrating that the induction of ERα caused by inhibition of EZH2 indeed invoked sensitivity to the action of tamoxifen.

In our previous study, we explored the application of human breast cancer patient-derived organoids (PDOs) as a promising platform for cancer precision medicine and suggested that the PDOs platform guided personal treatment is effective for patients with terminal breast cancer [26]. To minimize potential chronic adverse effects, we first evaluated the drug’s efficacy on 9 PDOs with different subtypes and then examined the therapeutic efficacy of low-dose EZH2i for combination treatment. Compared with tamoxifen monotherapy, the addition of a low dose of EZH2i increased the suppressive effects on six PDOs with different subtypes, including TNBC, Her2+ and Luminal B. This suggests that EZH2i combined with tamoxifen is a therapeutically actionable approach for breast cancer patients (Figs. 7J–M and S10). Increased transcription levels of *Esr1* in each PDOs after EZH2i treatment were further confirmed through RT-qPCR experiments (Figs. 7L and S10). Therefore, our results provide compelling support for the development of clinical trials exploring the combined effects of EZH2 inhibitors and tamoxifen in TNBC and potentially other ERα-negative breast cancers.

## Discussions

Experimental evidence suggested that different subtypes of breast cancer might arise from various classes of stem/progenitor cells, such as luminal progenitor cells and mammary basal progenitor cells [49]. However, some studies suggest the luminal progenitor cells may be a common source of both luminal and basal-like tumors. Indeed, the phenotypic switch from basal-like breast cancer to luminal type can be achieved by re-expressing ERα, FOXA1 or GATA3 [50–53]. Based on these observations, we hypothesized that the initiation and progression of TNBCs might be driven by genomic mutations and/or epigenetic modifications. The identification of these alterations responsible for TNBC formation could facilitate the therapeutic treatment of this deadly disease.

In this study, through a functional study using the SB DNA transposon system, we identified 64 candidate genes, whose alteration significantly accelerated TNBC formation in two mouse models, either carrying *Brca1* mammary-specific knockout [10] or *Fgfr2* mammary-specific activation [18]. Further screening through estrogen-response-element- (ERE−) Luc, progesterone-response-element- (PRE−) Luc and pNeuLite reporters to reflect the status of ERα, PR and HER2, we demonstrated that *RAD51B* knockout exhibited the strongest inhibitory effects on luciferase activities in both wild-type MCF-7 cells and FGFR2-overexpressing MCF-7 cells. Our in vitro validation showed that *Rad51b* deletion affects the expression of ERα in mouse *Fgfr2*-mutant HP5008 cells, *Brca1*-mutant 545 cells, and the human breast cancer MCF-7 cells. After establishing the in vivo system in both HP5008 and 545 cells to monitor ER signaling during tumor progression, we clearly showed that the loss of RAD51B expression triggers a switch from ERα-positive to ERα-negative tumors. Additionally, a positive correlation between RAD51B and ERα protein expression was observed in 136 breast cancer patient samples. Altogether, these data highlight the essential role of RAD51B in preserving ERα-positive cell identity and may contribute to lineage commitment switches in breast cancer. This finding raises important questions regarding the broader relevance of this phenomenon in breast cancer biology. While our in vivo data highlight the plasticity of *Fgfr2*-mutant HP5008 and *Brca1*-mutant 545 cells, further investigations using additional ERα+ cell lines are warranted to determine if this phenotypic shift is a common feature of ERα+ breast cancers. Moreover, our observations have yet to be validated in clinical samples. Future studies are recommended to assess whether the lineage commitment switches from ERα+ to ERα- occurs in human breast cancer samples, as this could have significant implications for disease progression and therapeutic resistance. Despite these limitations, our findings provide a foundation for understanding the dynamic nature of breast cancer phenotypes and underscore the need for further exploration into the molecular drivers of such plasticity.

RAD51B belongs to a RAD51 paralog family member that functions in DNA repair by homologous recombination (HR). RAD51B is known to function in maintaining genomic stability, and its loss in cells leads to a HR DNA repair deficiency (HRD) [54, 55]. RAD51B inactivation promotes tumorigenesis in breast and ovarian cancer [56]. The role of RAD51B-mediated genomic stability in regulating ERα expression and subsequent TNBC progression warrants further investigation. However, our study reveals a previously unrecognized function of RAD51B in modulating epigenetic modification processes, thereby expanding a new dimension of its biological functions. We showed that the loss of RAD51B expression leads to significant epigenetic modifications, particularly the trimethylation of histone H3 at lysine 27 (H3K27me3), mediated by the PRC2. This epigenetic alteration results in the repression of downstream targets, including ERα protein, which in turn drives the progression of TNBC. Studies have reported that inhibitors for epigenetic modifications, such as DNA methyltransferase inhibitors, histone deacetylase inhibitors (HDACi) and EZH2 inhibitors (EZH2i), could reactivate ERα expression in basal-like breast cancer [57–59]. Recently, it was shown that EZH2i treatment opens chromatin at enhancer sequences that regulate GATA3, and together with AKT inhibitor, drives basal-like cells into a luminal-like state [52]. However, the upstream regulator remained unclear. Our findings demonstrated that depletion of RAD51B by CRISPR/Cas9 enhanced the recruitment of PRC2 to *Esr1* promoter and resulted in the modification of H3K27me3 in this region, leading to the repression of ERα protein and the switch from ERα-positive breast cancer to an ERα-negative type. Mechanistically, the loss of RAD51B upregulated cellular ATP levels, thereby suppressing the AMPK pathway and reducing EZH2 phosphorylation at Thr311. The decrease in phosphorylation at the Thr311 region of the EZH2 protein enhances the assembly of PRC2 complex, leading to the repression of downstream factors, including ERα. However, further investigation is necessary to understand how RAD51B deletion affects cellular ATP levels. Recently, a paper suggested that catalytic glutamate mutants of RAD51B increased the fraction of ATP present within the complex [60], which may be one reason for RAD51B dysfunction affecting cellular ATP levels.

Tumor heterogeneity and the long-standing lack of effective therapies beyond chemotherapy have contributed to TNBC having the least favorable outcomes among breast cancer subtypes [61]. Instead of screening for new drugs targeting genetic or epigenetic alterations in basal-like breast tumors, strategies that induce a switch from hormone receptor-negative to hormone receptor-positive breast tumors could provide established and effective treatment options for a large patient group in need of improved therapy. In this study, we demonstrated that RAD51B is essential for ERα expression during tumorigenesis. The depletion of RAD51B reduced ERα expression by enhancing the recruitment of PRC2 to the *Esr1* promoter. Importantly, through animal models and patient-derived organoid models, we demonstrated that targeting RAD51B downstream signaling with EZH2i re-induces functional ERα protein expression in ERα-negative breast cancer, thereby rendering tumors targetable by tamoxifen. These data support that converting ERα-negative breast cancer into ERα-positive by targeting the RAD51B-EZH2 axis is a therapeutically actionable approach for TNBC patients and those with ERα-negative breast cancer.

In conclusion, our findings underscore the importance of RAD51B in regulating ERα expression through epigenetic mechanisms and highlight the potential of EZH2 inhibitors to restore ERα protein expression and enhance the efficacy of endocrine therapy in TNBC. We propose a strong rationale for clinical evaluation of EZH2 inhibitors in combination with endocrine therapy for the treatment of TNBC and potentially other ERα-negative breast cancers.

## Supplementary information

Supplementary Figures

Uncropped western blot

Table S1. Sequence for sgRNA

Table S2. Primers for RT-qPCR

Table S3. Key resource table

## Data Availability

The RNA-seq data generated in this study are available from the lead contacts upon reasonable request. The code used for all processing and analysis is available upon request.

## Code availability

The RNA-seq data generated in this study are available from the lead contacts upon reasonable request. The code used for all processing and analysis is available upon request.
