# Supplementary material for: RAD51B-EZH2 axis as a potential therapeutic target for TNBC through cell fate conversion
Source: Cell Death Dis. 2025 Nov 30;17(1):64. doi: 10.1038/s41419-025-08259-8 (PMC12827460; doi:10.1038/s41419-025-08259-8)
Supplement: Supplementary file 1 — Supplementary Figures [file 41419_2025_8259_MOESM1_ESM.docx]

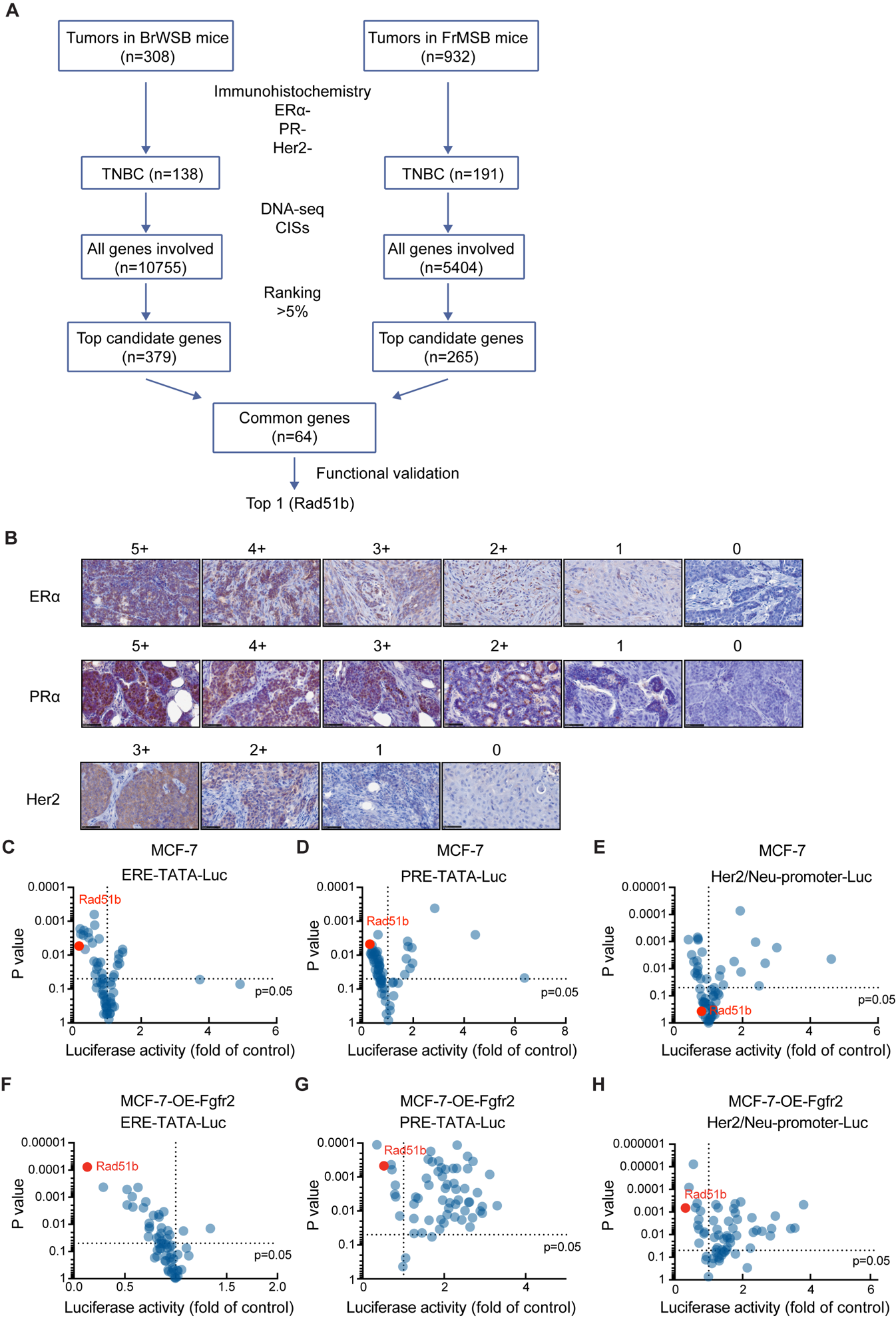


**Supplementary Figure 1. SB-driven candidate gene identification and reporter screening assay.** (A) The strategy for identifying candidate genes for inducing TNBC (B) IHC staining with antibodies against ERα, PR, Her2 and Ki67. (C) Dot plot depicting luciferase signal from ERE reporter screening of 64 overlapped candidate driver genes in MCF-7 cells. (D) Dot plot depicting luciferase signal from PRE reporter screening of 64 overlapped candidate driver genes in MCF-7 cells. (E) Dot plot depicting luciferase signal from Her2/Neu promoter reporter screening of 64 overlapped candidate driver genes in MCF-7 cells. (F) Dot plot depicting luciferase signal from ERE reporter screening of 64 overlapped candidate driver genes in MCF-7-OE-Fgfr2 cells. (G) Dot plot depicting luciferase signal from PRE reporter screening of 64 overlapped candidate driver genes in MCF-7-OE-Fgfr2 cells. (H) Dot plot depicting luciferase signal from Her2/Neu promoter reporter screening of 64 overlapped candidate driver genes in MCF-7-OE-Fgfr2 cells.


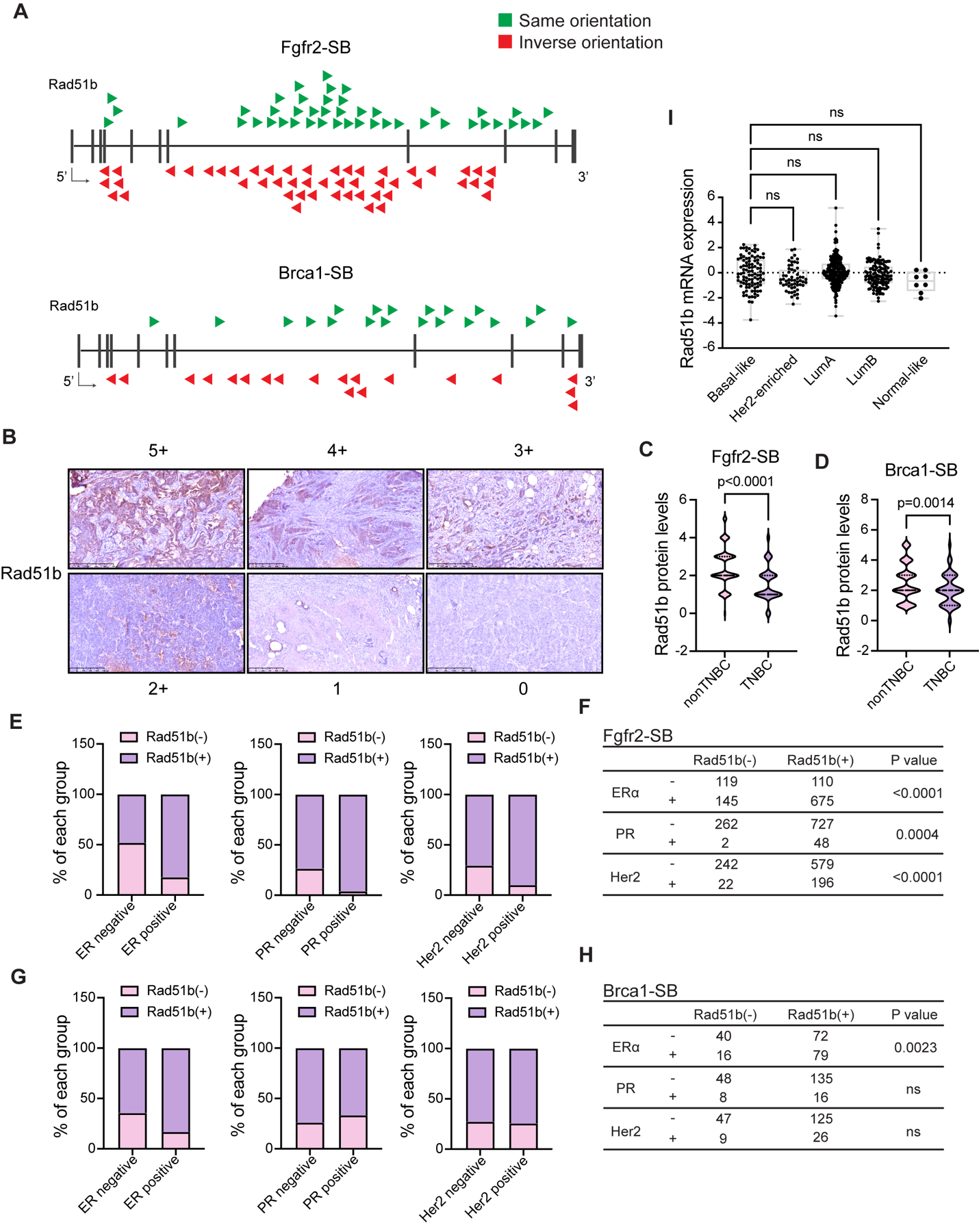


**Supplementary Figure 2. Information of *Rad51b*-driven tumors in SB transposon models.** (A) Structure of *Rad51b* and transposon insertion pattern within the *Rad51b* gene. Green arrows indicate that the promoter in the transposon is in the same orientation as the host gene, and red arrows indicate the inverse orientation. (B) IHC staining with antibodies against RAD51B. (C) RAD51B abundance in TNBC and non-TNBC tumors from *Fgfr2*-SB group measured by IHC analysis. (D) RAD51B abundance in TNBC and non-TNBC tumors from *Brca1*-SB group measured by IHC analysis. Statistical significance among groups determined by two-tailed Student’s *t* test. (E-F) RAD51B expression is positively correlated with ERα, PR and Her2 in tumors from *Fgfr2*-SB group (*P*<0.0001, *P*=0,0004 and *p*<0.0001 respectively). (G-H) RAD51B expression is positively correlated with ERα in tumors from *Brca1*-SB group (*P*=0.0023). Statistical significance among groups determined by Chi-squared test. (I) *Rad51b* mRNA expression levels on breast cancer patients from TCGA database. Data are presented as the means ± SEM with statistical significance among groups determined by one-way ANOVA test.

**
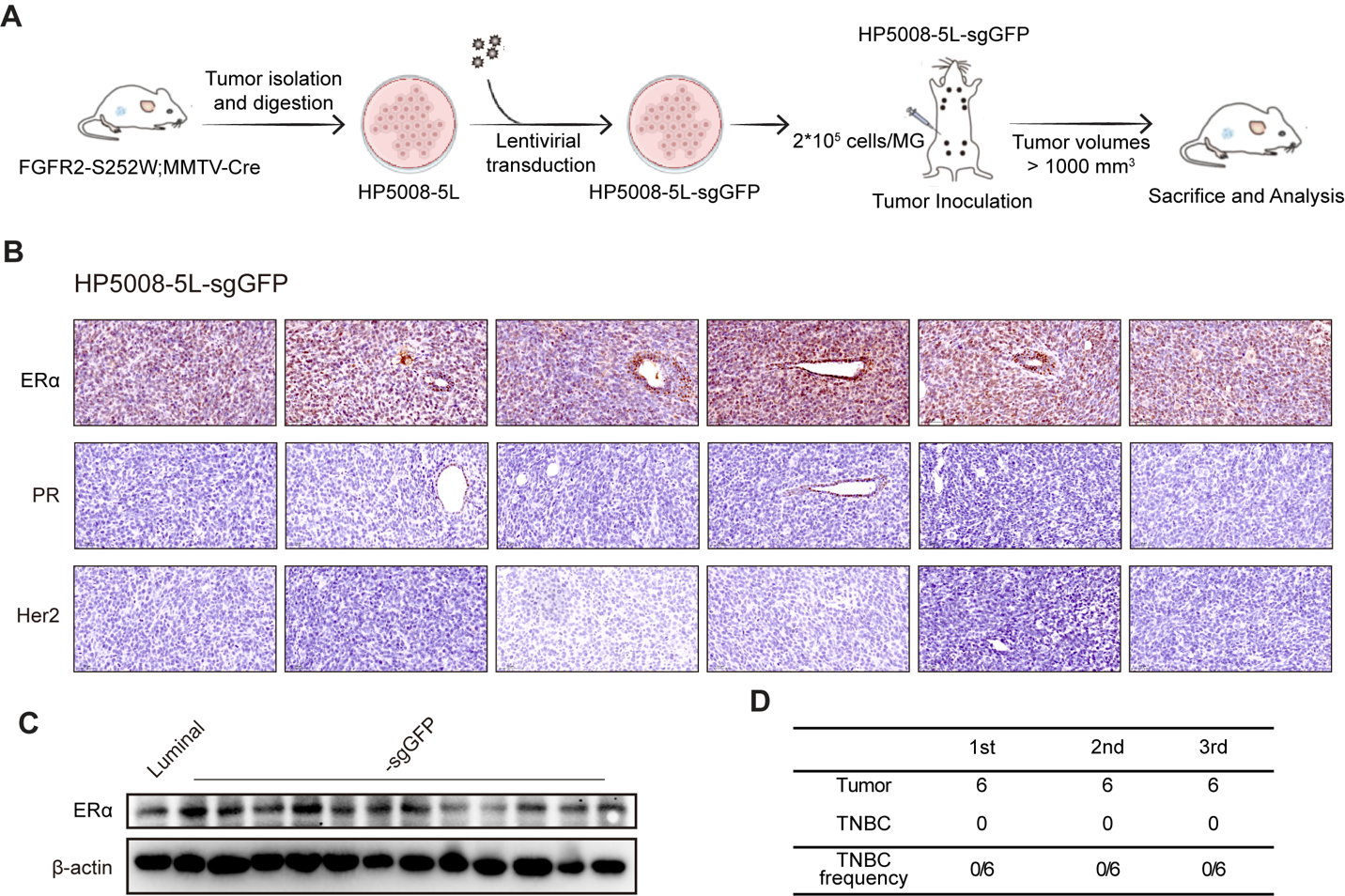
**

**Supplementary Figure 3. Molecular subtypes of HP5008 cells.** (A-B) Schematic diagram showing how HP5008 cell line was developed (A) and representative IHC staining images (B) showing its molecular subtype confirmed by IHC analysis after three rounds of injection. Scale bar, 50 μm. (C) Whole tissue lysates from tumors were immunoblotted with ERα antibody. Lysates from luminal tumor were added as positive control. (D) Table summary the TNBC frequency of tumors collected from three rounds of injection.


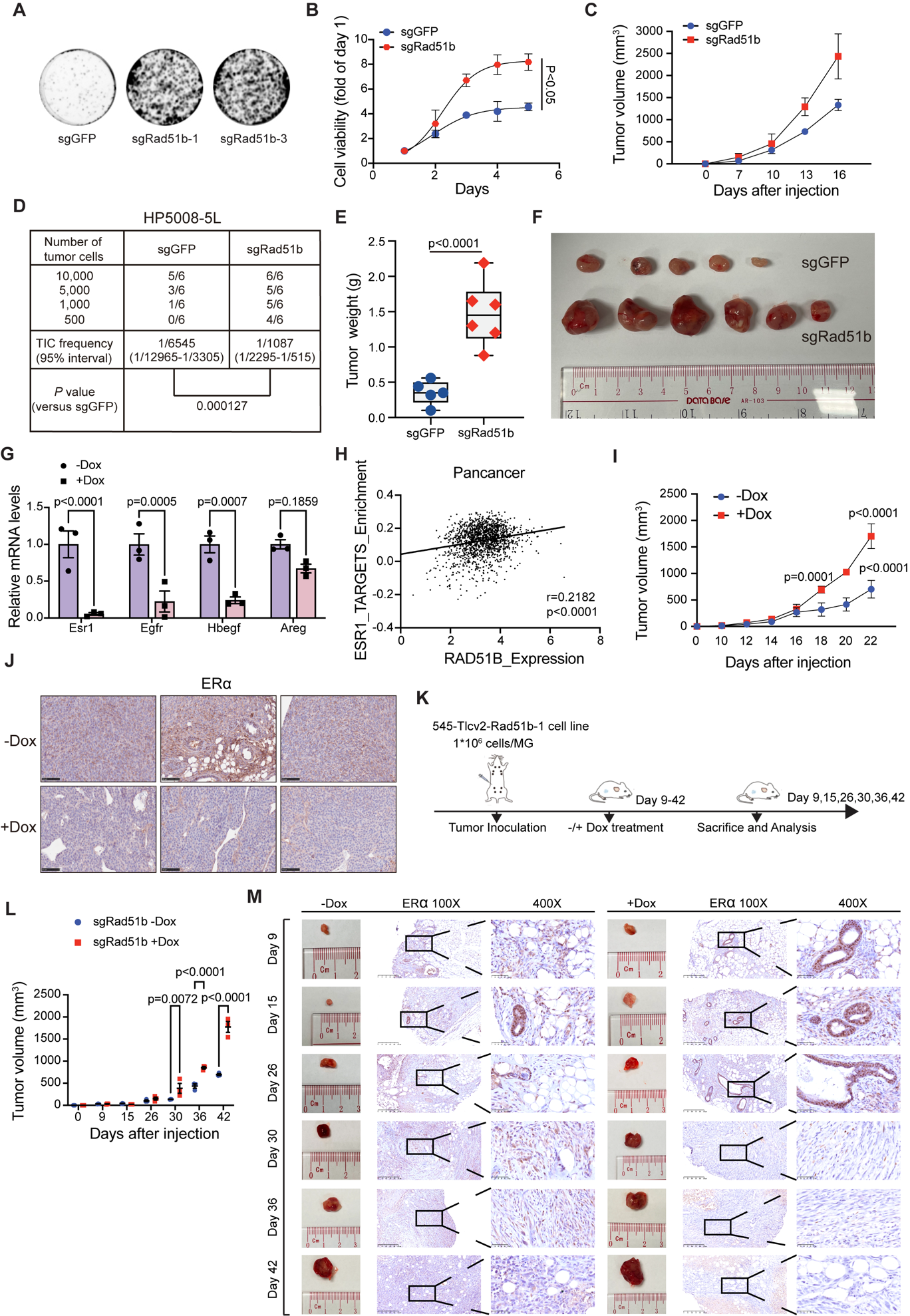


**Supplementary Figure 4. RAD51B deletion promotes tumor formation and suppresses ERα expression.** (A-B) Representative images of the colony formation (A) and cell proliferation (B) of HP5008 cells with *Rad51b* sgRNA and non-target control sgRNA. (C) Effects of *Rad51b* depletion on the growth of tumors formed from HP5008 cells with *Rad51b* sgRNA and non-target control sgRNA (n=6 per group). Data are presented as the means ± SEM with statistical significance among groups determined by one-way ANOVA test. (D) Tumor incidence in nude mice implanted with HP5008 cells stably expressing the indicated sgRNA (n=number of mammary fat pad injections as indicated in the table). TIC frequency and statistical significance were determined by Pearson’s Chi-square test using ELDA software. (E-F) Tumor weight (E) and tumor images (F) of tumors from indicated group (1000 cells per mammary fat pad). (G) The mRNA expression of *Esr1* and its downstream genes in HP5008 cells stably expressing the indicated sgRNA. Data are presented as the means ± SEM with statistical significance among groups determined by two-tailed Student’s *t* test. (H) Scatter plot showing the positive correlation between *Rad51b* mRNA expression and ESR1_TARGETS_Enrichment score in pan-cancer cell lines. Pearson’s correlation coefficient r and two-tailed *P* value were shown. (I) Tumor growth curves of tumor bearing mice treated with or without dox to induce *Rad51b* knockout (n=3 per group). (J) IHC staining of tumors from indicated groups with antibodies against ERα. (K) The strategy for establishing mouse models for *in vivo* Dox induced *Rad51b* knockout and tumor samples collection. (L) Tumor volume of tumor bearing mice treated with or without dox to induce *Rad51b* knockout (n=3 per group). Data are presented as the means ± SEM with statistical significance among groups determined by two-way ANOVA test comparing tumor volumes between each group on each day separately (I and L). (M) Representative tumor images and IHC staining with antibodies against ERα.


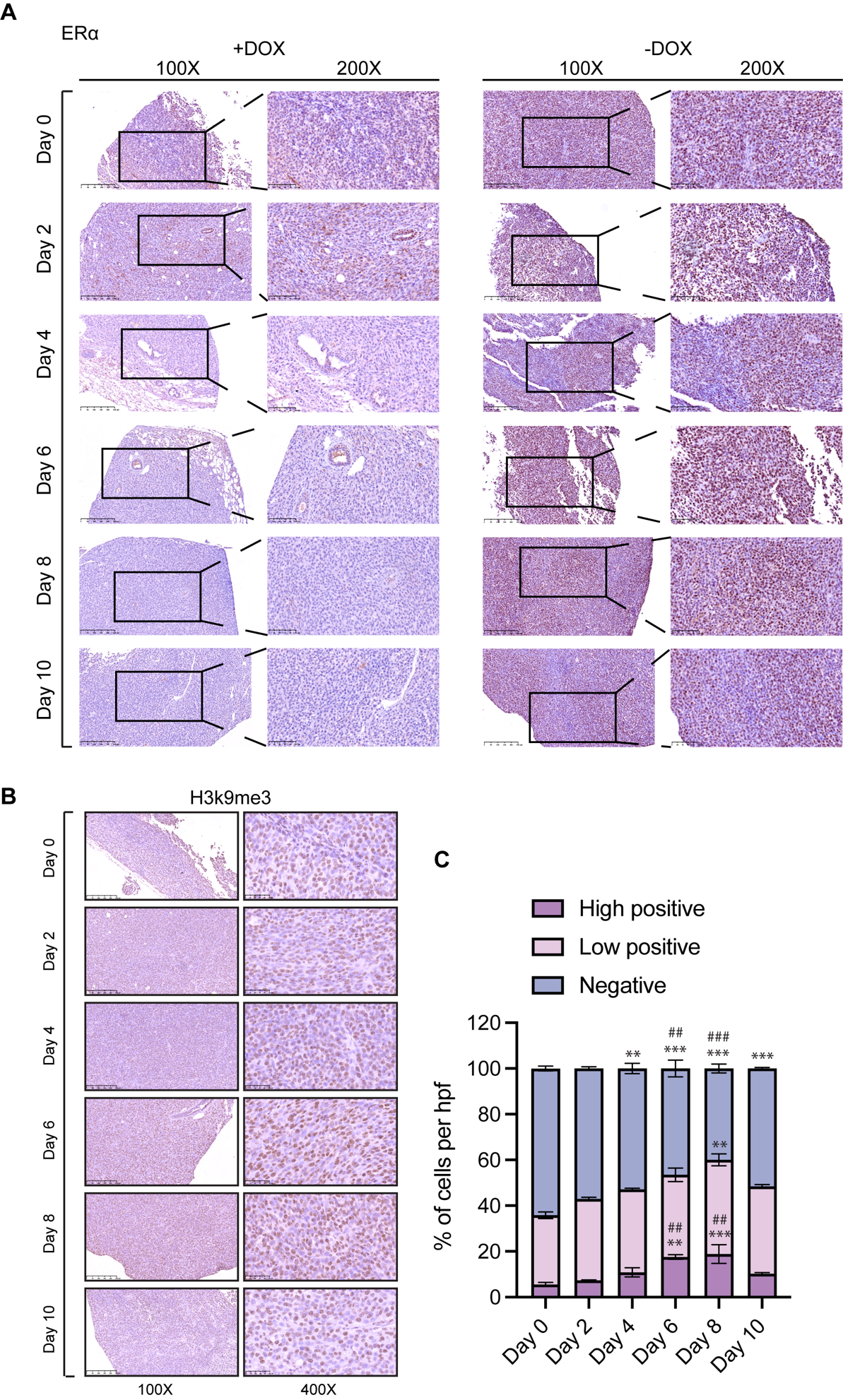


**Supplementary Figure 5. Effect of dox-induced Rad51b knockout on the expression of ERα and H3K9me3.**

(A) IHC staining with antibodies against ERα. (B-C) H3K9me3 levels of tumor samples from indicated group were measured by immunohistochemistry staining (B) and quantified results (C). Data are presented as the means ± SEM with statistical significance among groups determined by Dunnett’s multiple-comparisons test. * P<0.05, ** P<0.01, ***P<0.001 vs. the signal-matched Day 0 group; ## P<0.01, ### P<0.001 vs. the signal-matched Day 2 group.


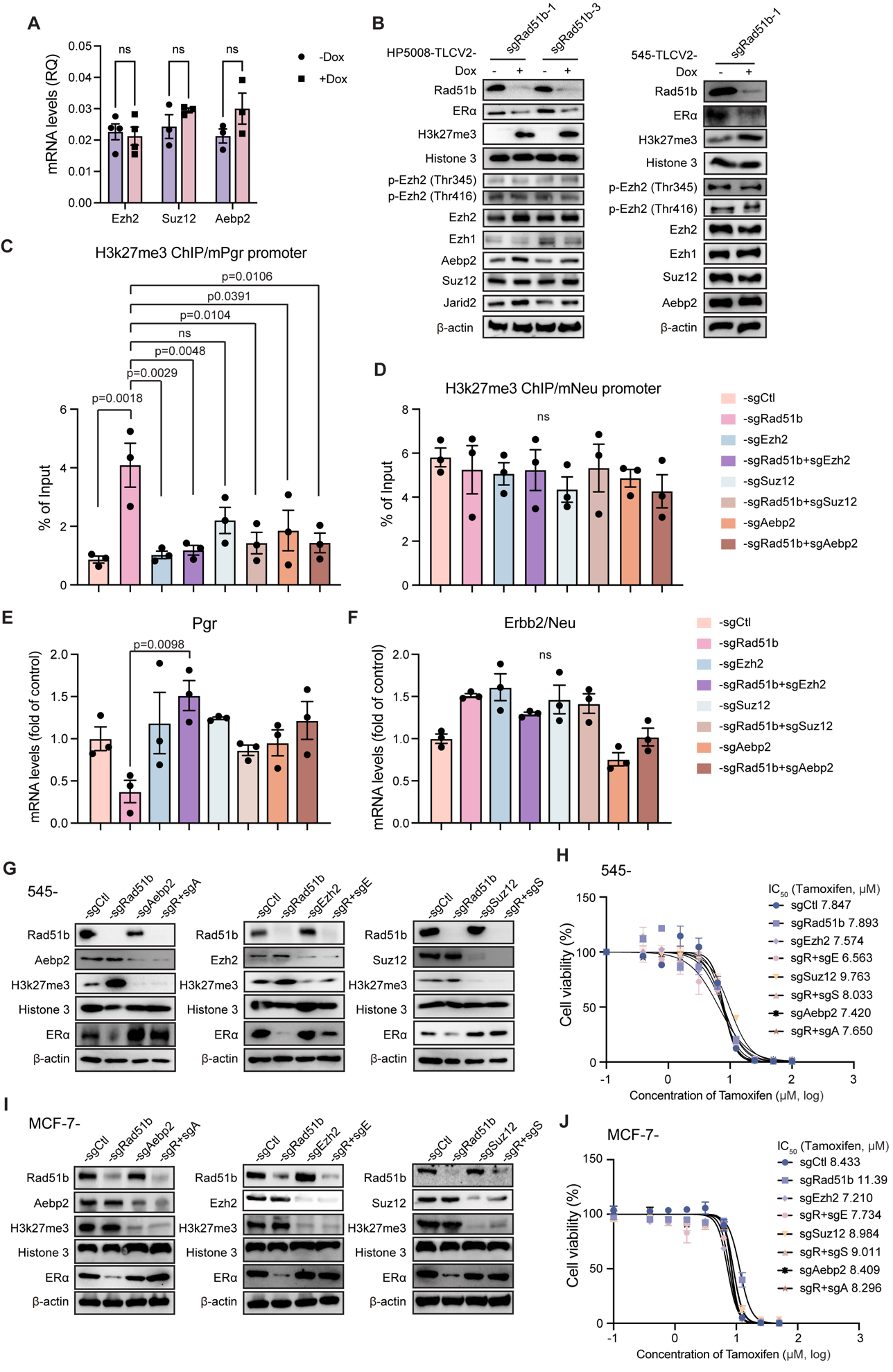


**Supplementary Figure 6. Effect of PRC2 on gene expression regulated by RAD51B deletion.** (A) *Ezh2, Suz12* and *Aebp2* expression levels in HP5008 cells with or with dox-induced *Rad51b* knockout were measured by RT-qPCR. Data are presented as the means ± SEM with statistical significance among groups determined by one-way ANOVA test. (B) Western blotting showing the expression of indicated protein after dox-induced *Rad51b* knockout in HP5008 cells and 545 cells. (C-D) ChIP-qPCR analysis of the enrichment of H3K27me3 at the promoter region of *Pgr* (C) and *Neu* (D). (E-F) *Pgr* (E) and *Neu* (F) expression levels in each group were measured by RT-qPCR. In (C) to (F), Data are presented as the means ± SEM with statistical significance among groups determined by one-way ANOVA. (G) Western blotting showing the expression of indicated protein after knocking-out *Rad51b*, *Ezh2*, *Suz12* or *Aebp2* in 545 cells (H) Tamoxifen effects on cell viability with indicated cell lines for 48h treatment. IC_50_ values for tamoxifen are indicated. Data are presented as means ± SEM of three independent experiments. (I) Western blotting showing the expression of indicated protein after knocking-out *Rad51b*, *Ezh2*, *Suz12* or *Aebp2* in MCF-7 cells (J) Tamoxifen effects on cell viability with indicated cell lines for 48h treatment. IC_50_ values for tamoxifen are indicated. Data are presented as means ± SEM of three independent experiments.


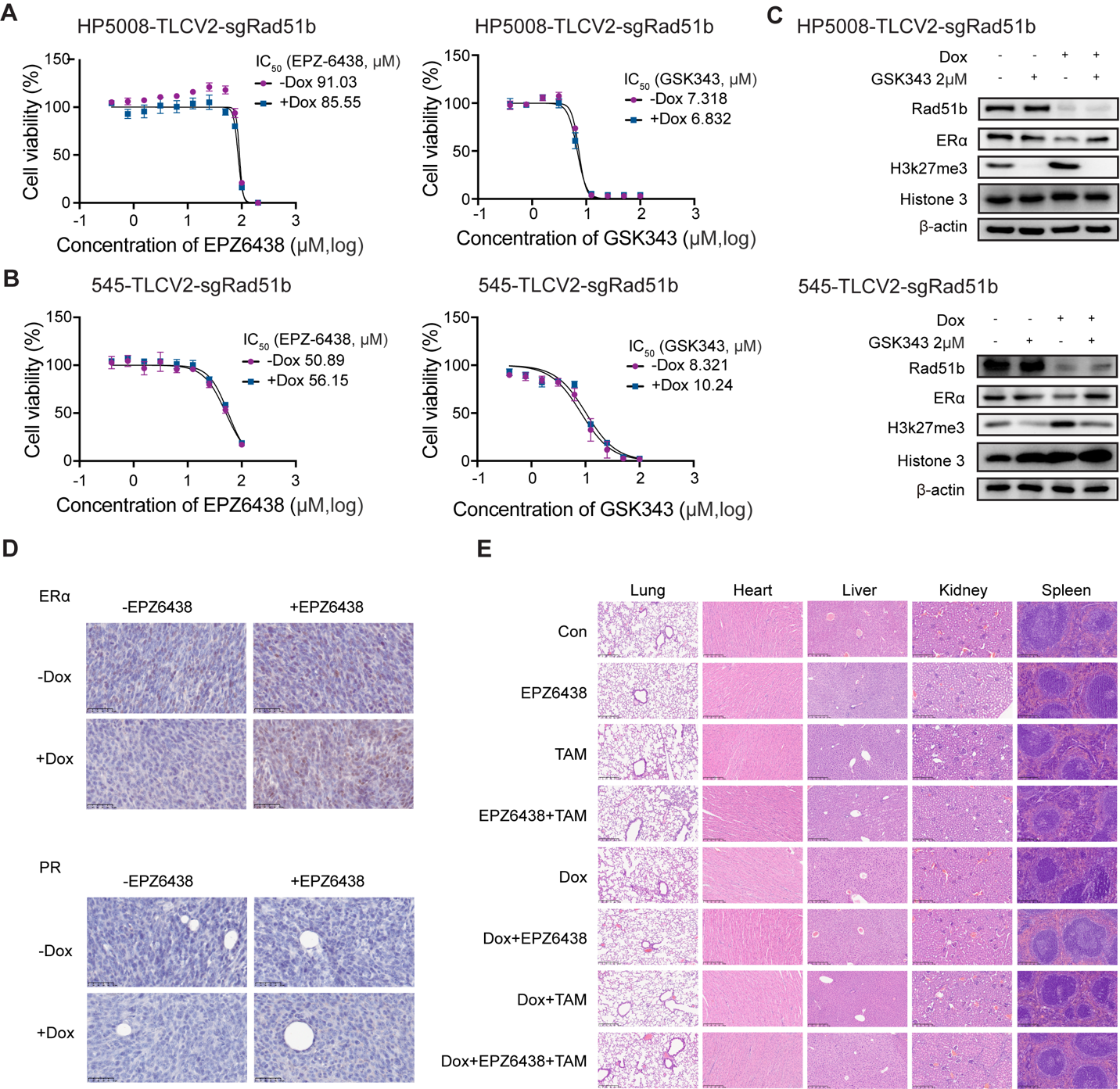


**Supplementary Figure 7. Effect of EZH2 inhibitor on cell viability and ERα expression.** (A) EZH2 inhibitor (EZH2i) effects on cell viability in HP5008 cells with or without dox induced *Rad51b* knockout. (B) EZH2i effects on cell viability in 545 cells with or without dox induced *Rad51b* knockout. IC_50_ values for 48h treatment are indicated. Data are presented as means ± SEM of three independent experiments. (C) Western blotting showing the expression of indicated protein after dox-induced *Rad51b* knockout and EZH2i treatment in HP5008 cells and 545 cells. (D) IHC staining of tumors from indicated groups with antibodies against ERα and PR. Scale bar, 50 μm (E) H&E staining of major organs. Scale bar, 250 μm.


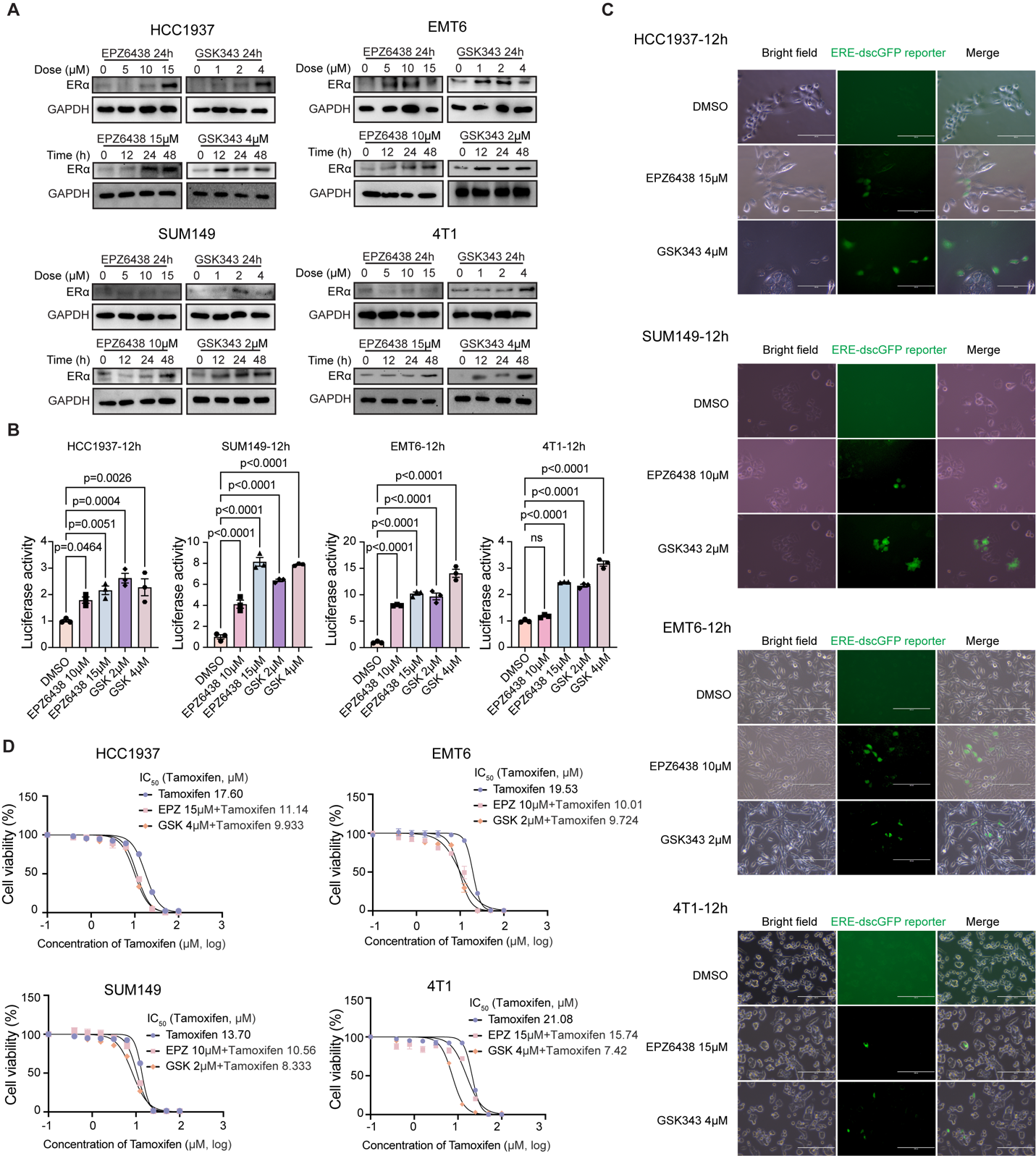


**Supplementary Figure 8. Targeting EZH2 confers anti-estrogen sensitivity on TNBC cell lines.** (A) Western blotting showing the expression changes of ERα in different human and mouse TNBC cell lines with indicated EZH2 inhibitor treatment. (B) The luciferase reporter activities of ERE in different human and mouse TNBC cell lines. Data are presented as the means ± SEM with statistical significance among groups determined by one-way ANOVA test. (C) Representative fluorescence microscopy images of cells treated with EZH2 inhibitors for 12 h, the medium containing 15 nM estradiol. The ERE-dscGFP signal is depicted in green. Scale bar, 200 μm. (D) Effect of EZH2 inhibition on tamoxifen sensitivity in different human and mouse TNBC cell lines. IC50 values for tamoxifen are indicated. Data are presented as means ± SEM of three independent experiments.


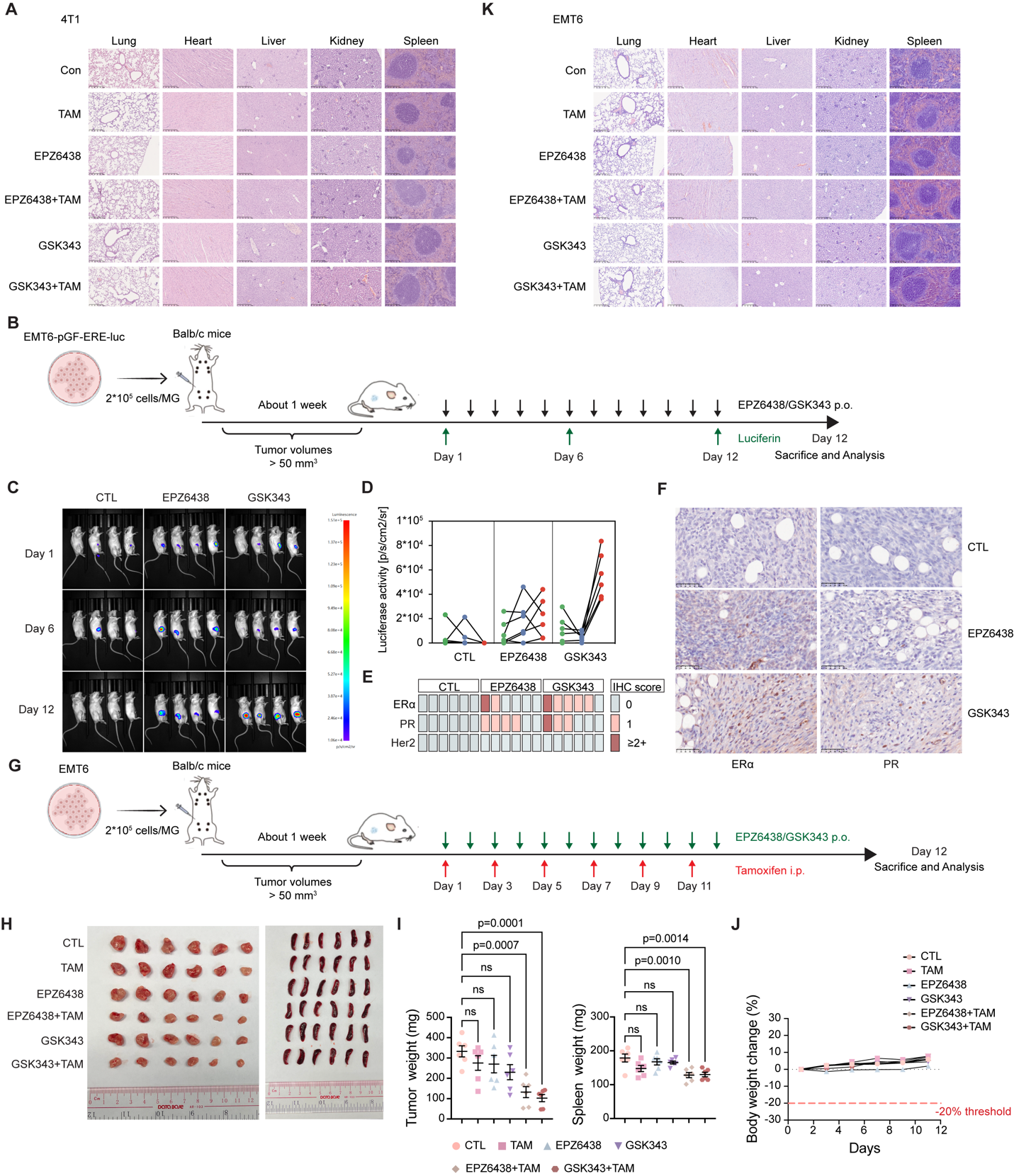


**Supplementary Figure 9. *In vivo* drug treatment in EMT6 allograft model.** (A) H&E staining of major organs. Scale bar, 250 μm. (B) The strategy for establishing mouse models for *in vivo* ERα signaling detection. (C) Representative images and quantitation analysis (D) of bioluminescence signals of tumor-bearing mice. (E) Summary of ERα, PR and Her2 abundance in tumors from each group measured by IHC analysis. (F) Representative images of ERα and PR abundance in tumors from each group measured by IHC analysis. (G) The strategy for establishing mouse models for *in vivo* investigation of the effects of combination therapy. Tumor images (H), tumor weight, spleen weight (I) and body weight change (J) of mice in each group. Data are presented as the means ± SEM with statistical significance among groups determined by one-way ANOVA test. (K) H&E staining of major organs. Scale bar, 250 μm.


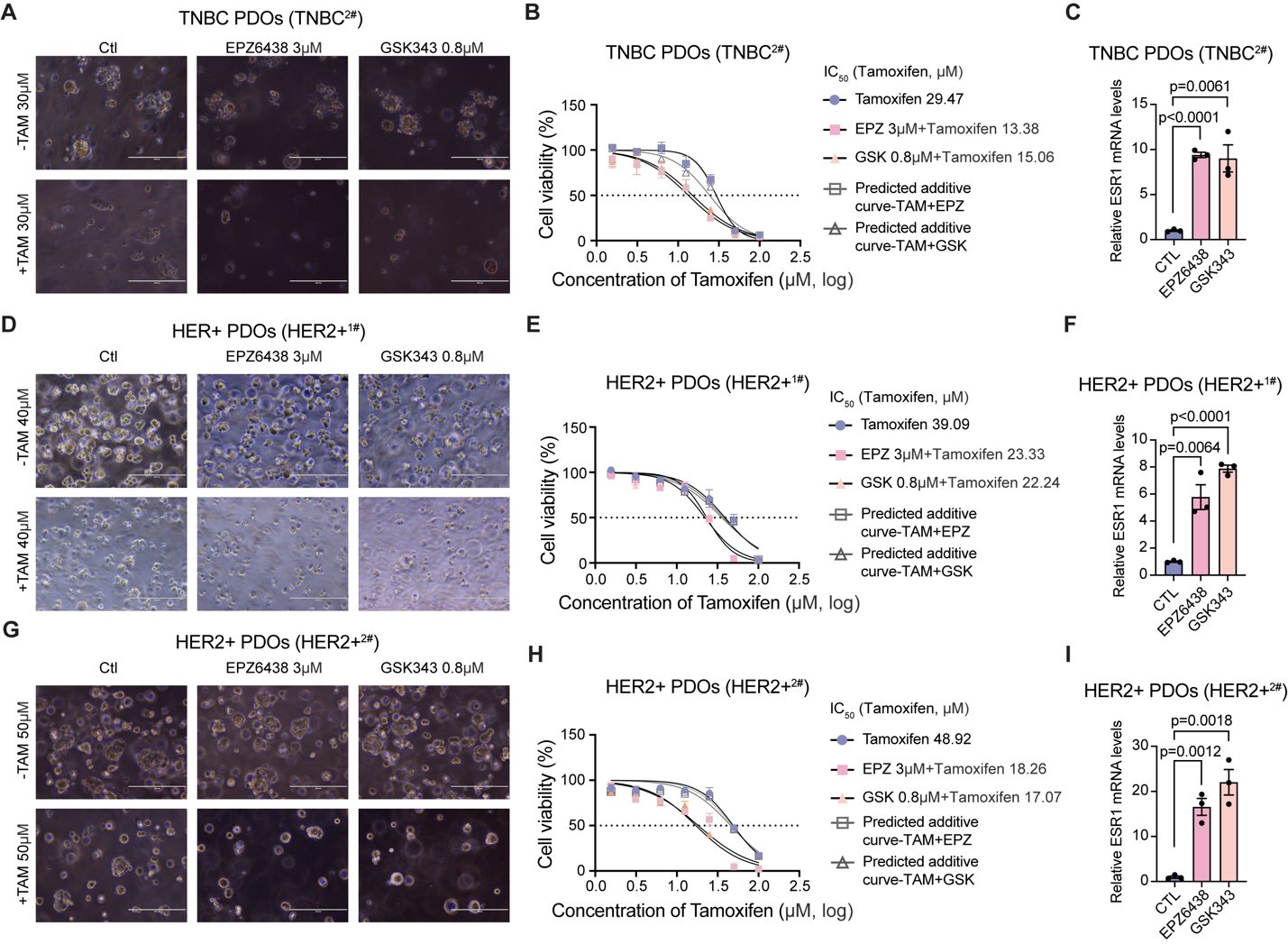


**Supplementary Figure 10. Combined drug treatment of patient-derived organoids (PDOs).** (A, D and G) Representative images and quantification (B, E and H) of cell viability of PDOs treated with DMSO, EPZ6438, GSK343 and tamoxifen (TAM) for 4 days. Scale bar, 200 μm. IC_50_ values for TAM are indicated. Data are presented as means ± SEM of three independent experiments. (C, F, and I) *ESR1* expression levels in each group were measured by RT-qPCR. Data are presented as the means ± SEM with statistical significance among groups determined by one-way ANOVA test.
