# Supplementary material for: RAD51B-EZH2 axis as a potential therapeutic target for TNBC through cell fate conversion
Source: Cell Death Dis. 2025 Nov 30;17(1):64. doi: 10.1038/s41419-025-08259-8 (PMC12827460; doi:10.1038/s41419-025-08259-8)
Supplement: Supplementary file 2 — Uncropped western blot [file 41419_2025_8259_MOESM2_ESM.docx]

Figure 1F


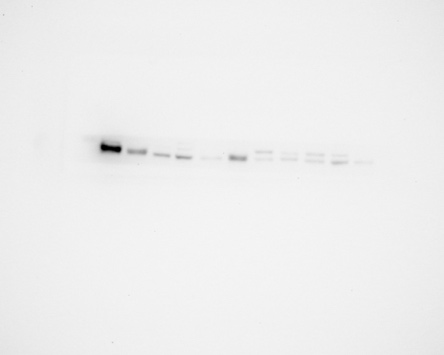

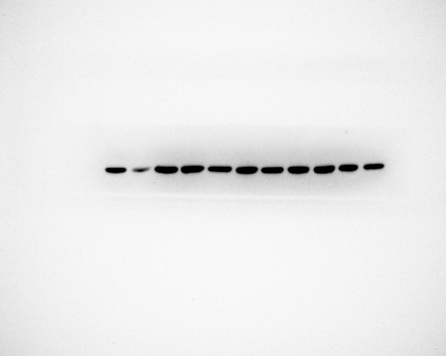

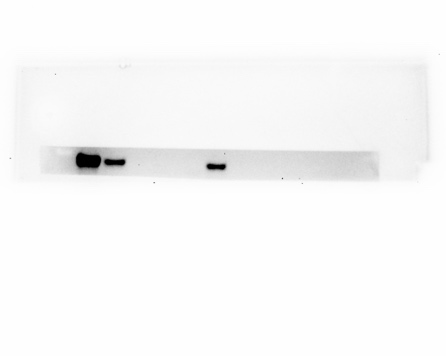

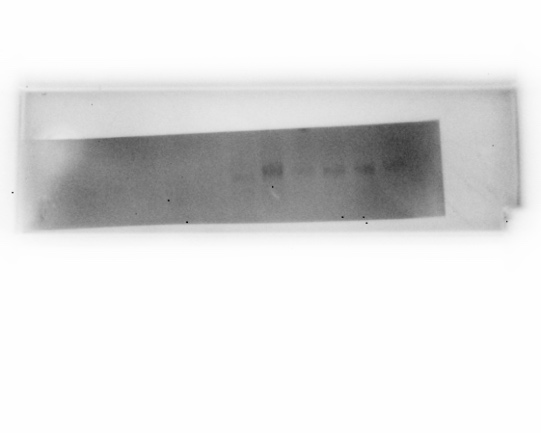

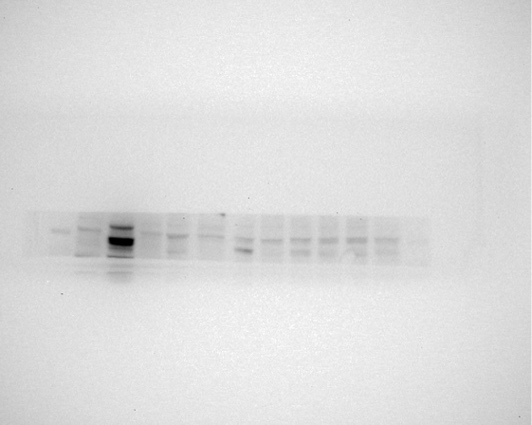


PR

Her2

RAD51B

GAPDH

ER

Marker

Figure 2A


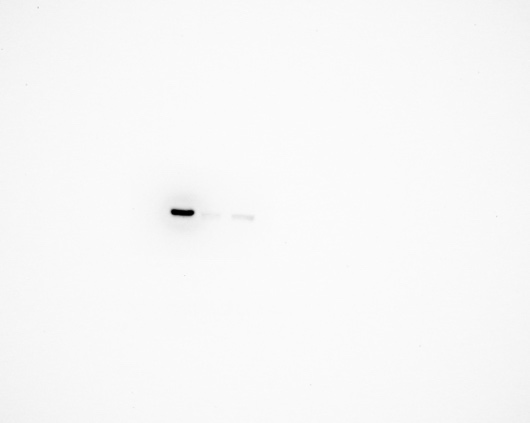

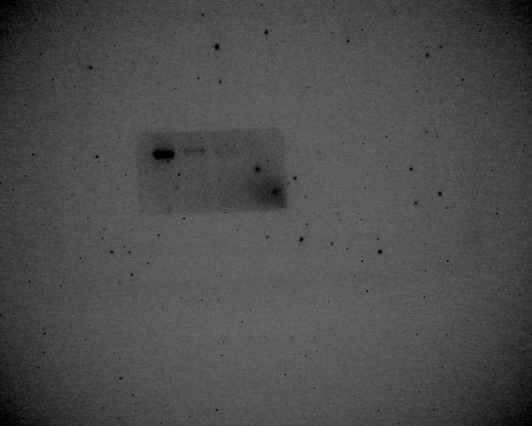

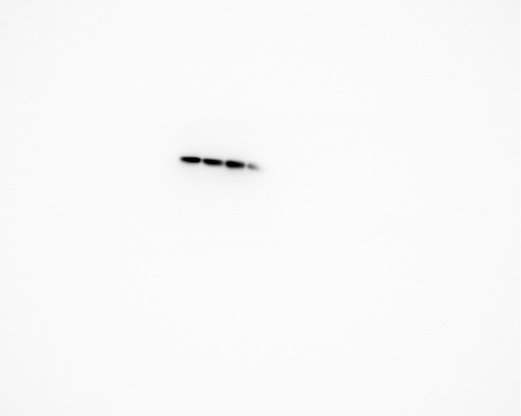


Beta-actin

RAD51B

ER

Figure 2B


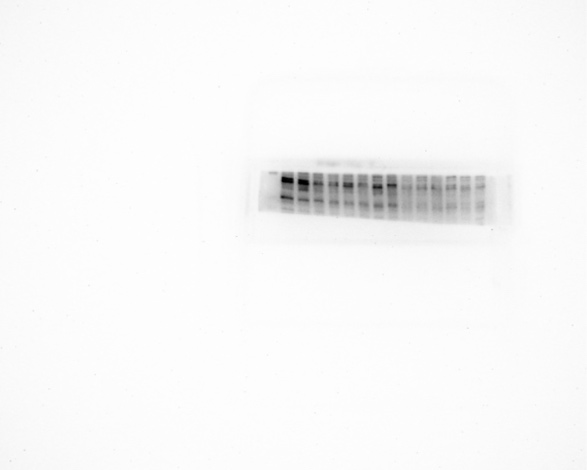

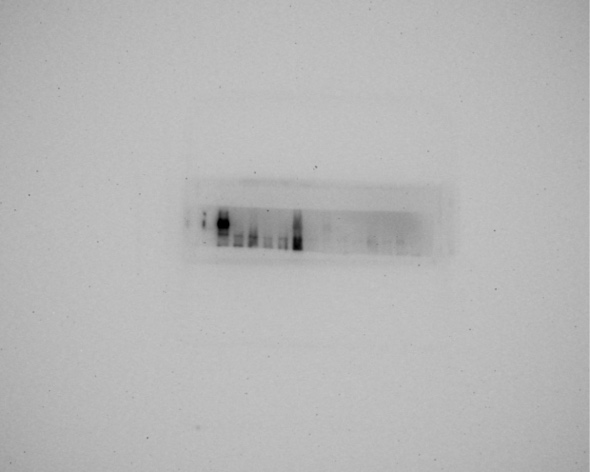


PR

Marker

RAD51B


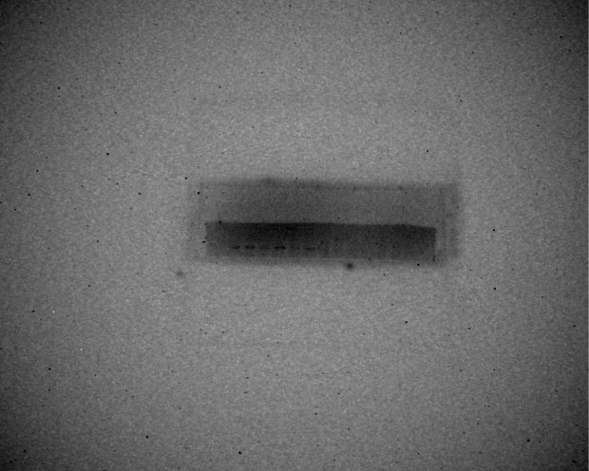

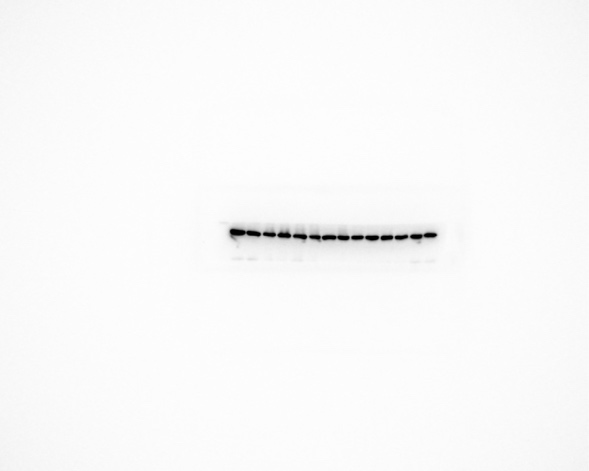


Beta-actin

Her2


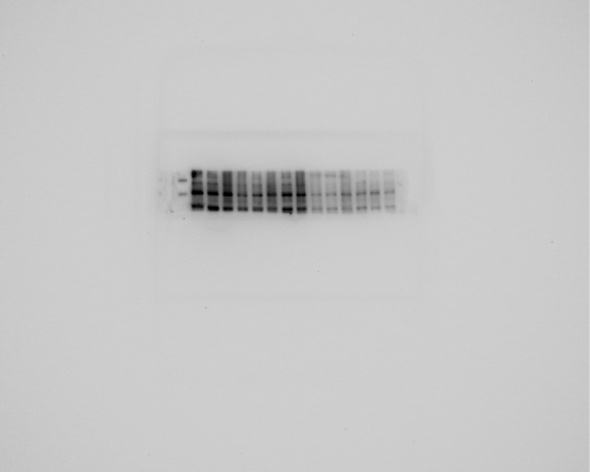


ER

Marker

Figure 2E


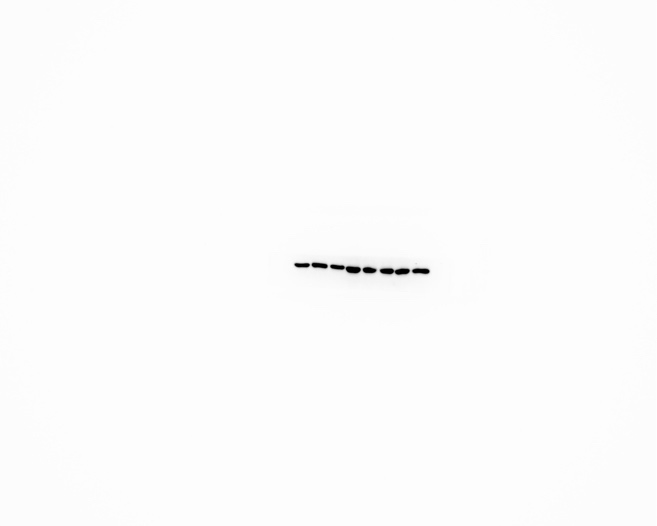

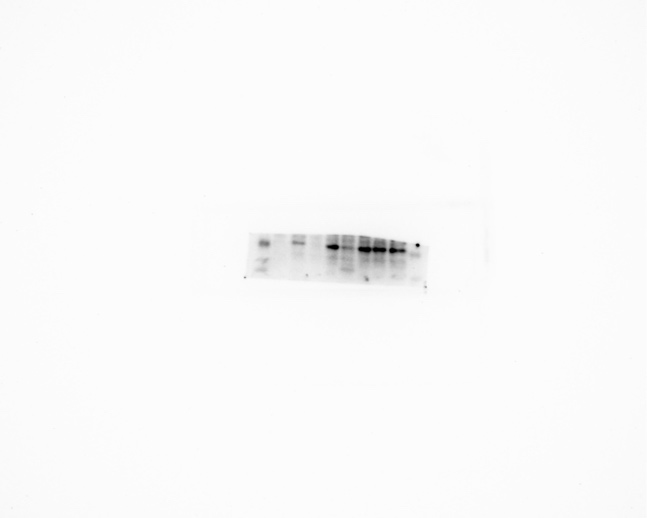


0 0 1 1 2 2 3 3

0 24 0 24 0 24 0 24

Time (h)

RAD51B

DOX ug/ml

Beta-actin

Figure 2N


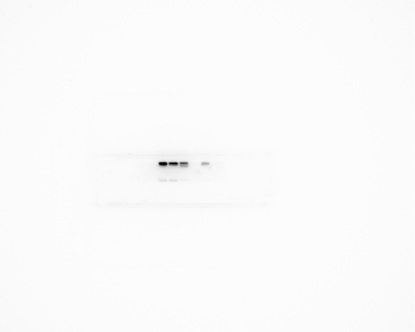

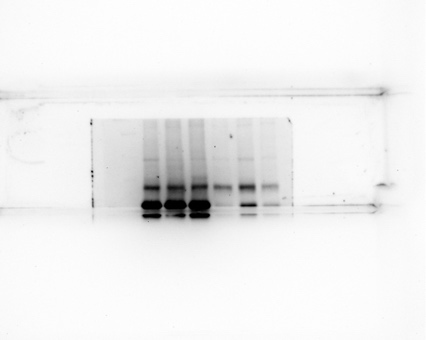

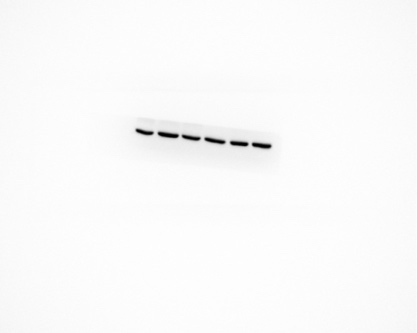


Beta-actin

PR

RAD51B


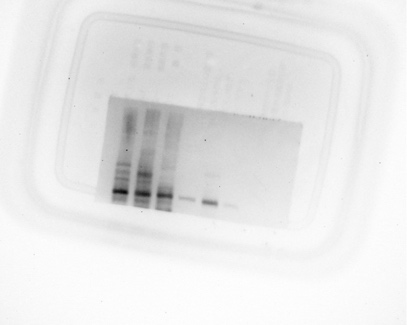

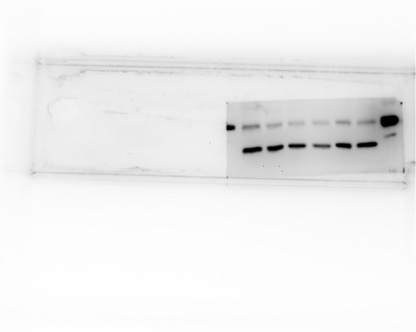


Her2

ER

Figure 3D

Beta-actin


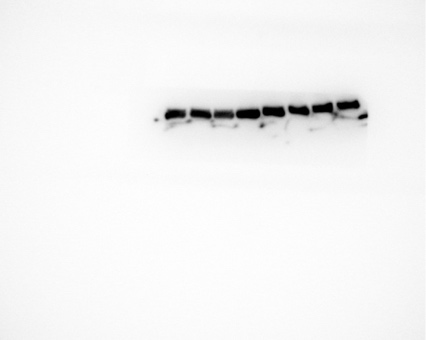

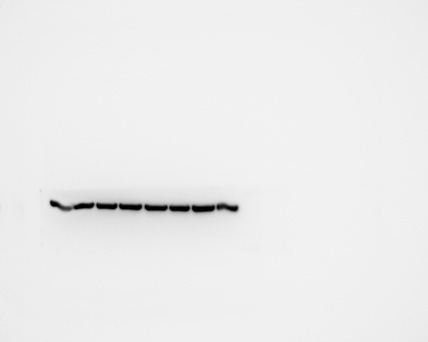

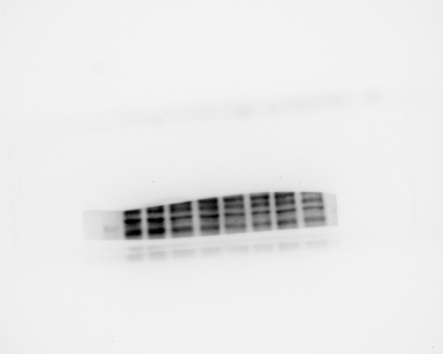


Marker

Ç√

RAD51B

Histone 3


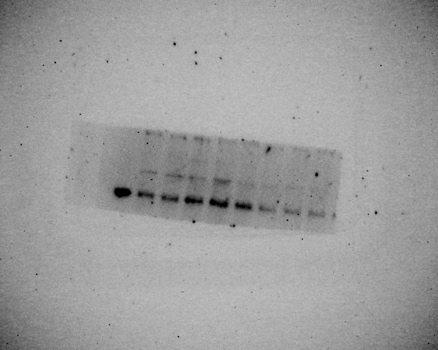

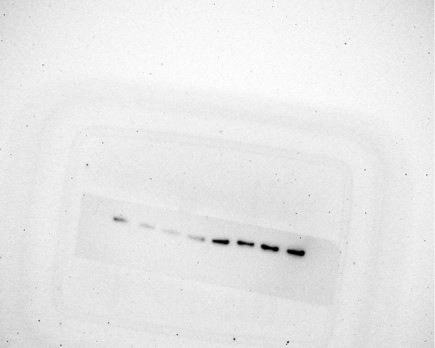

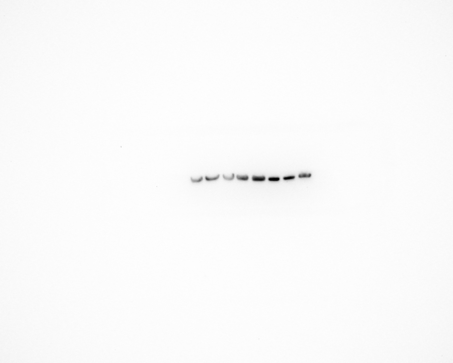


H3K9me3

H3K27me3

Ç√

Marker

ER

Figure 3H (Left)


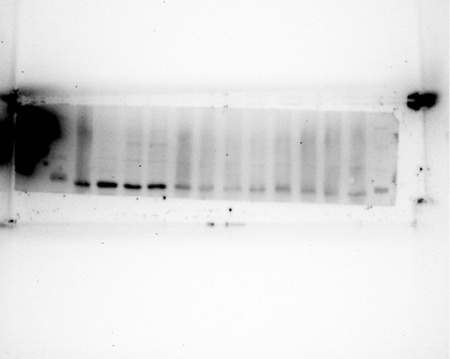

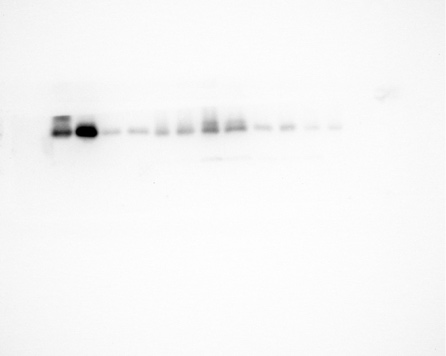

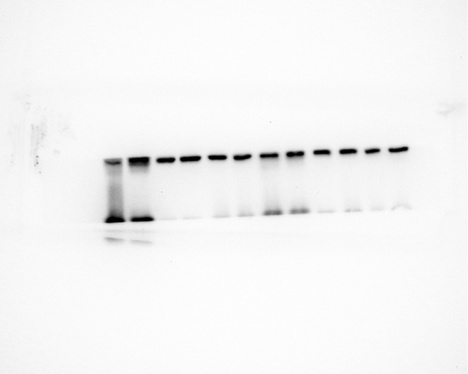


Tubulin

RAD51B

Marker

Marker

ER


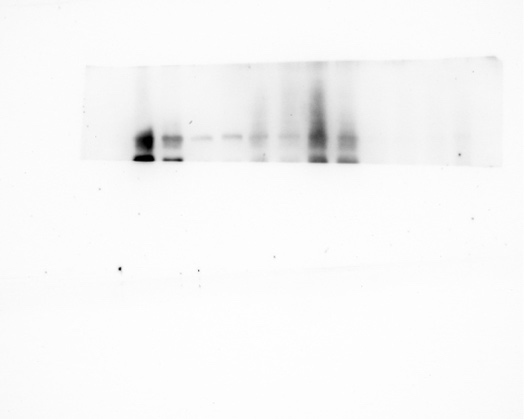

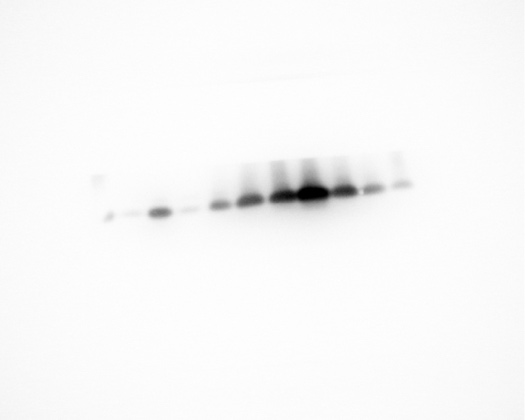

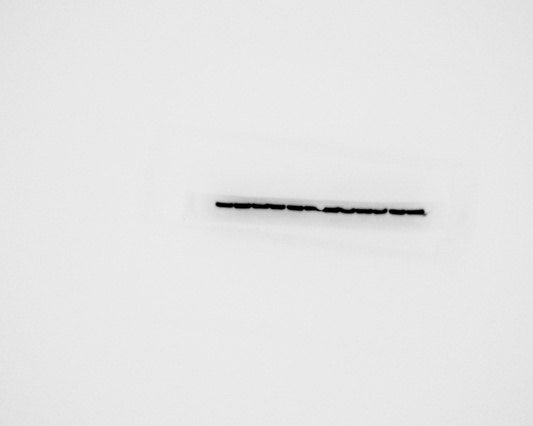

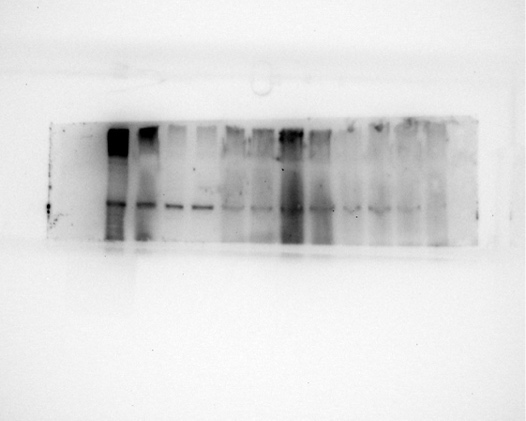


Her2

Histone3

H3k27me3

PR


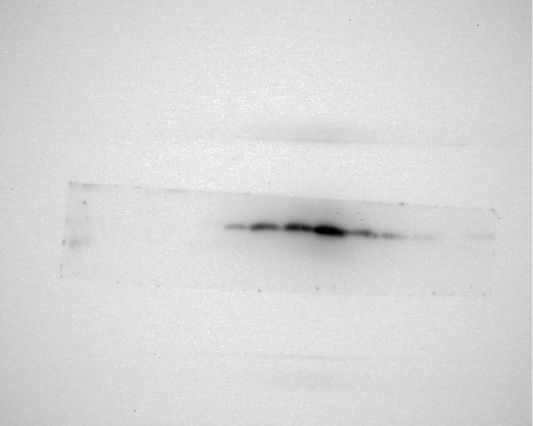


H3k9me3

Figure 3H (Right)


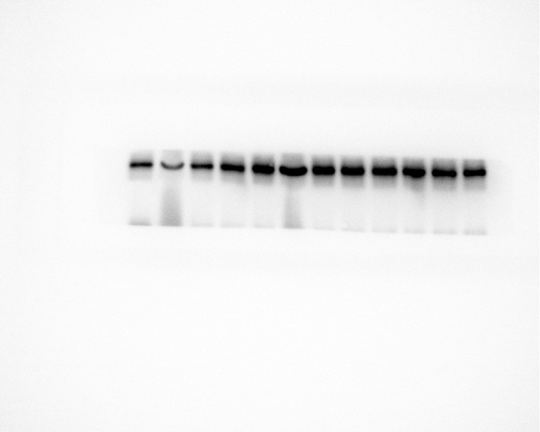

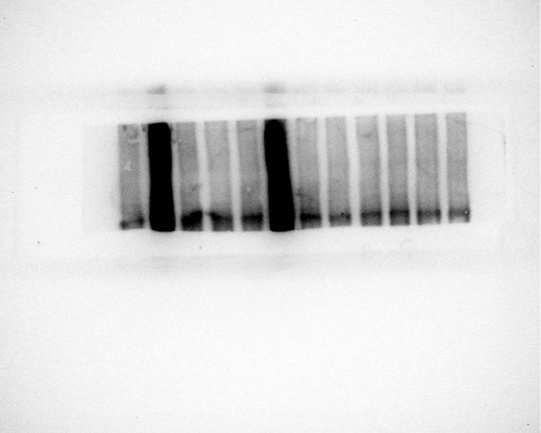

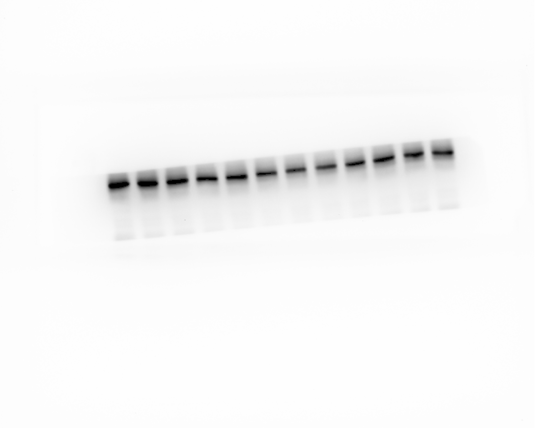

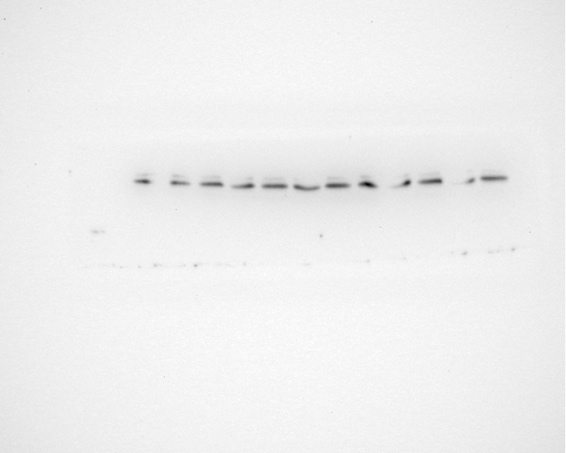

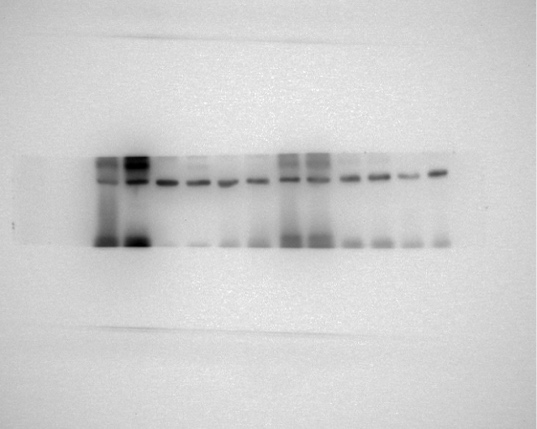

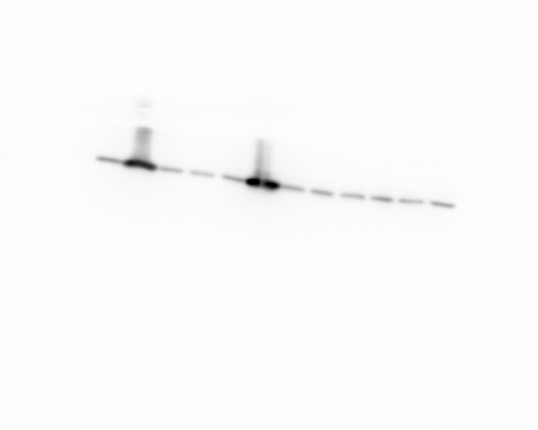


H3k27me3

PR

ER

Her2

Tubulin

RAD51B


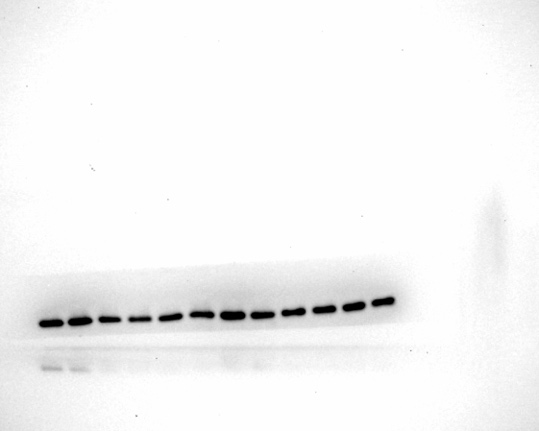

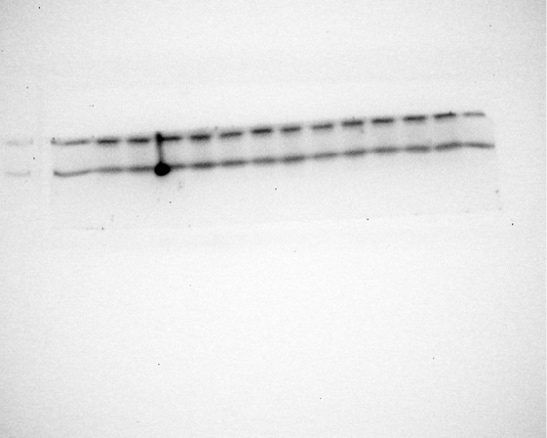


Ç√

Marker

Marker

H3k9me3

Histone3

Figure 4G (left)


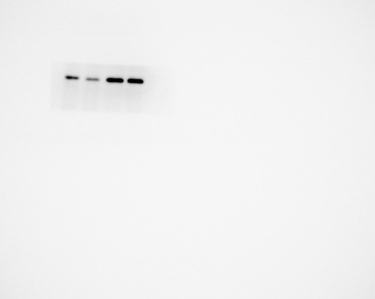

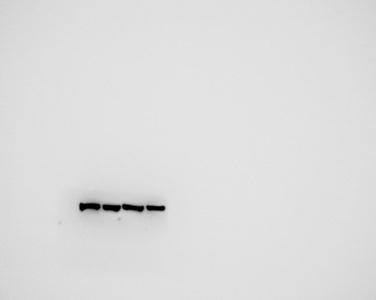

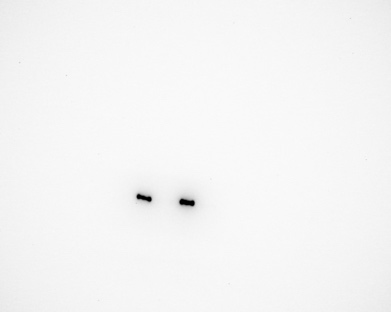


RAD51B

Beta-actin

ER


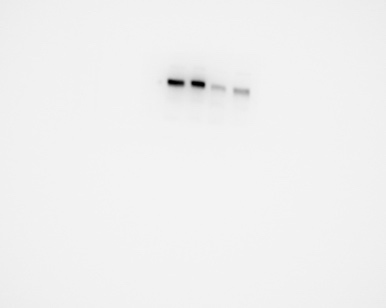

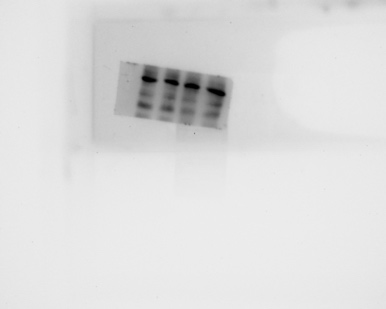

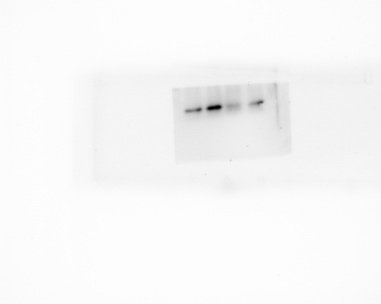


H3k27me3

Histone3

EZH2

Figure 4G (Middle)


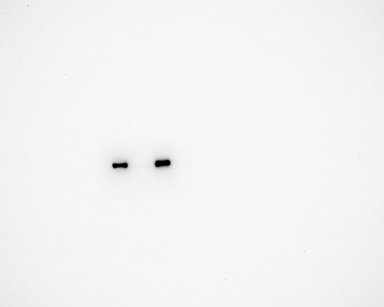

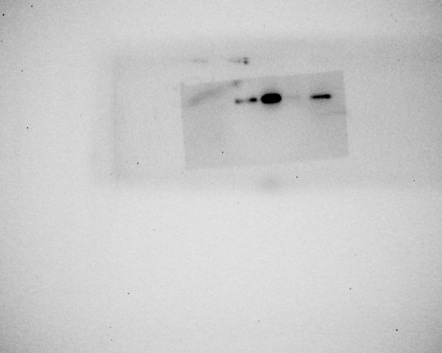

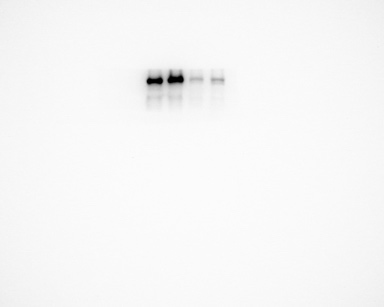


SUZ12

H3k27me3

RAD51B


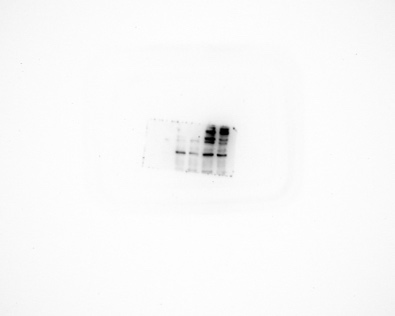

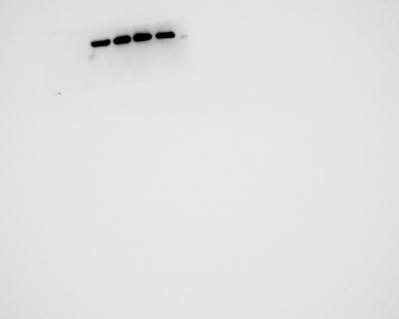

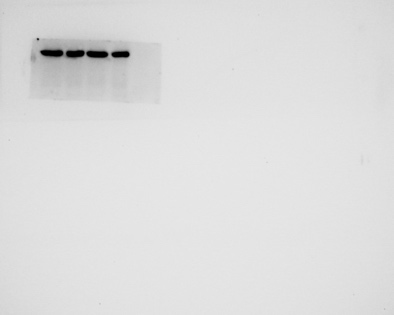


Beta-actin

Histone3

ER

Figure 4G (Right)


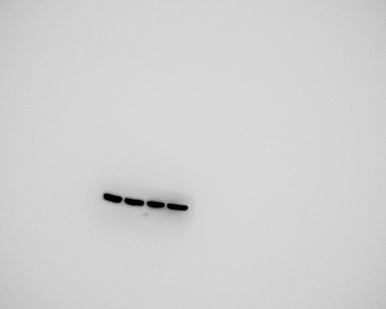

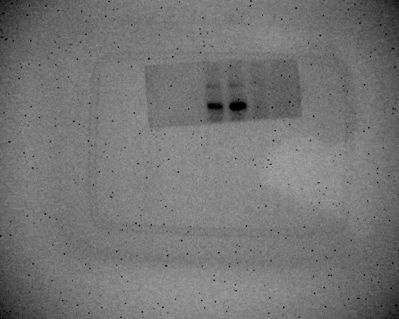

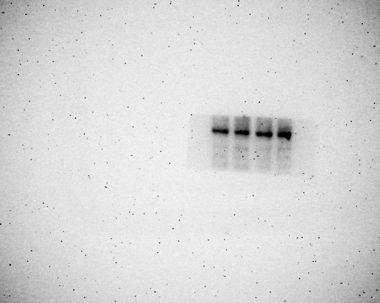


Histone3

AEBP2

Beta-actin


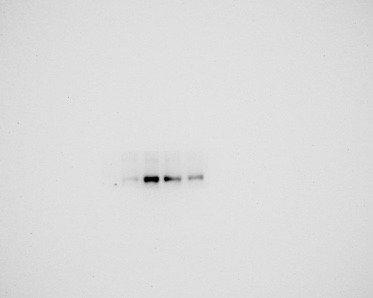

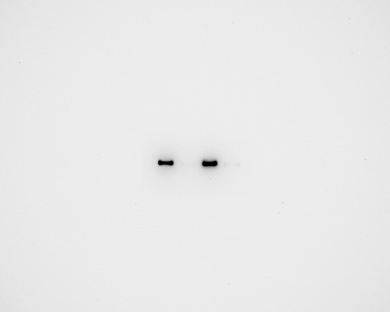

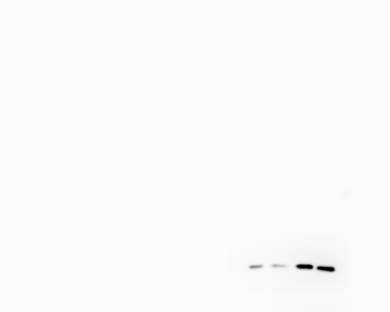


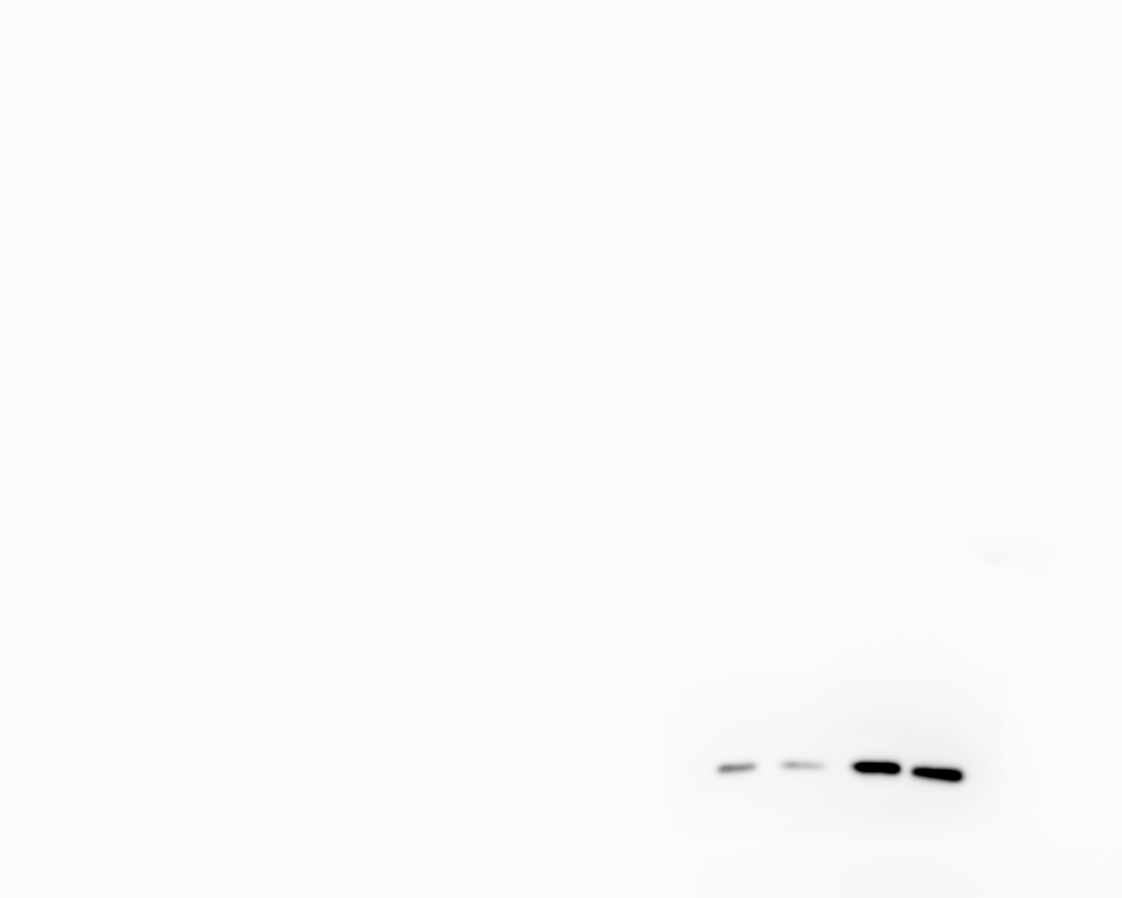
ER

RAD51B

H3k27me3

Figure 5A (Left)


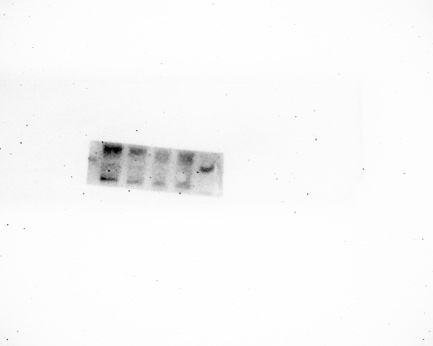

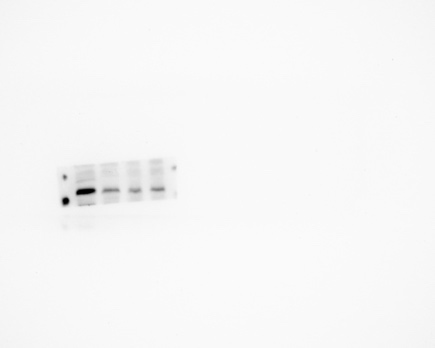

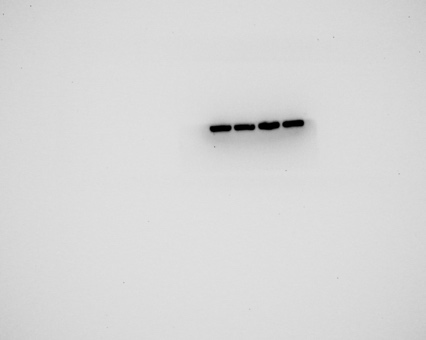


Ampk

ER

Marker

Ç√

p-Ampk


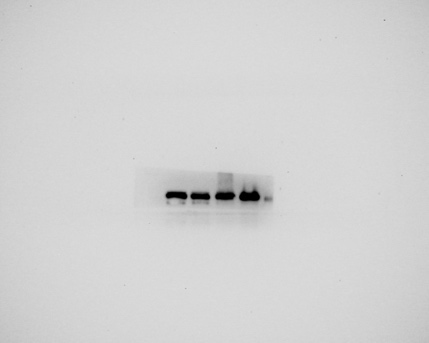

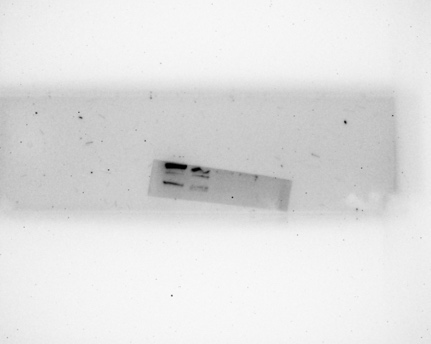

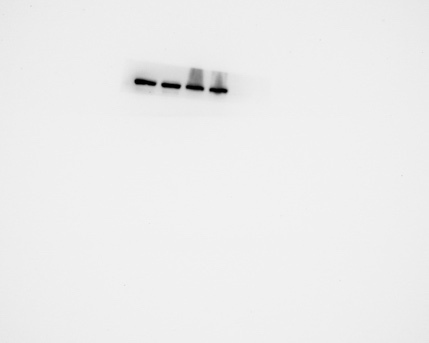


EZH2

Ç√

p-EZH2

Histone3


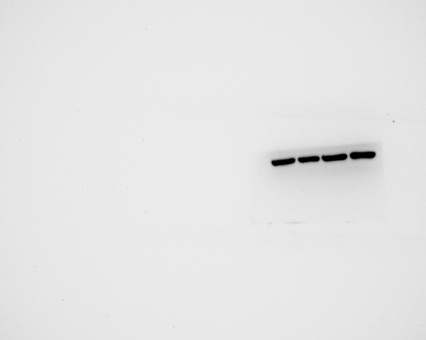

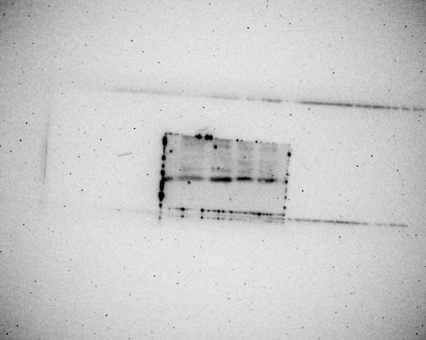

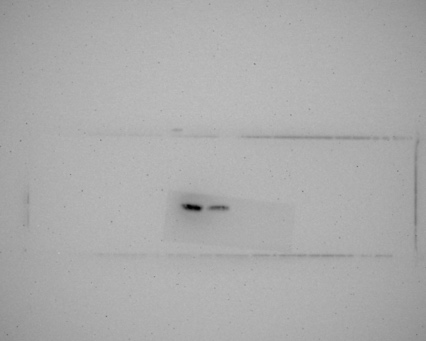


RAD51B

H3k27me3

Beta-actin

Figure 5A (Right)

p-Ampk


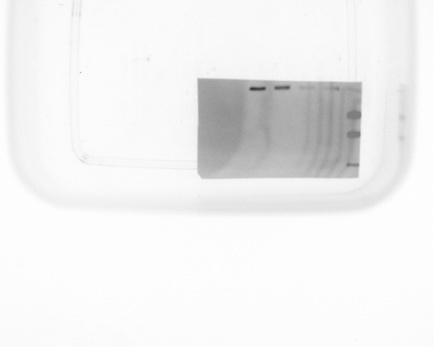

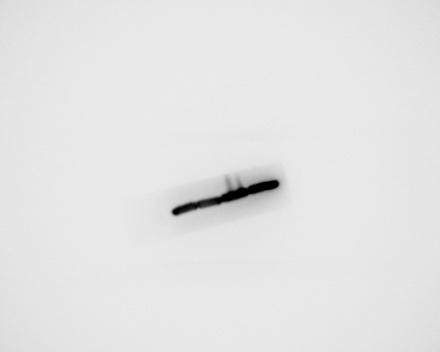

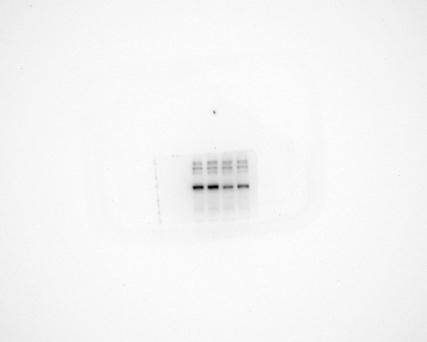


p-EZH2

Histone3


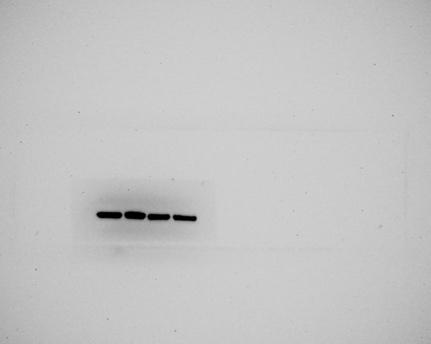

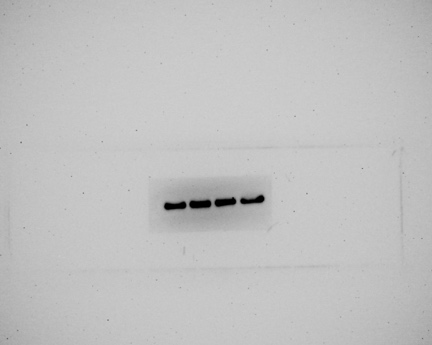

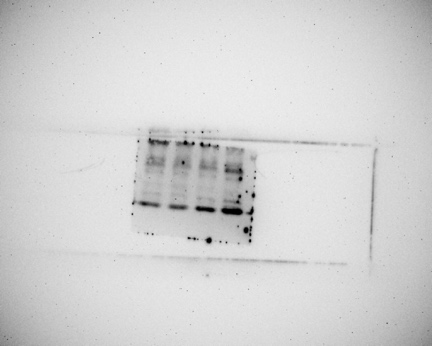


H3k27me3

Ampk

Beta-actin


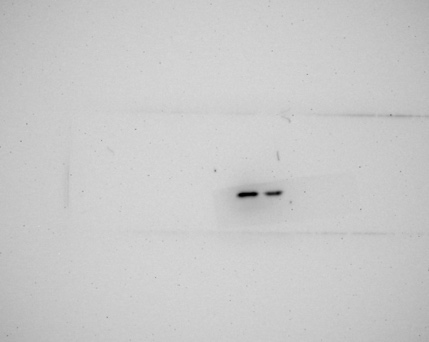

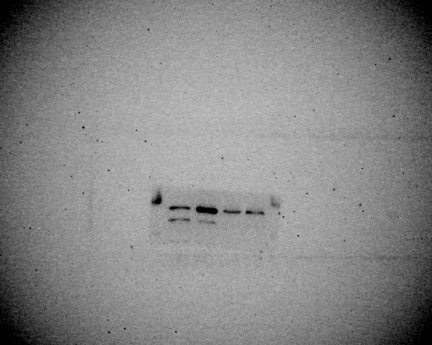

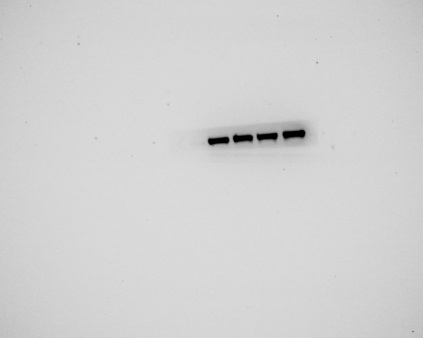


EZH2

ER

Ç√

RAD51B

Figure 5B (Left)


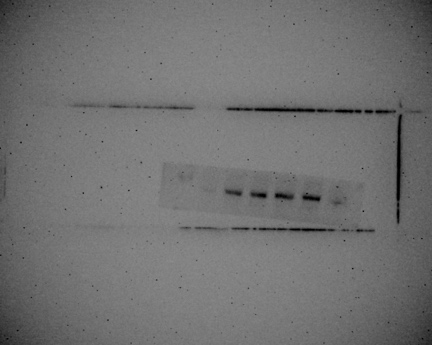

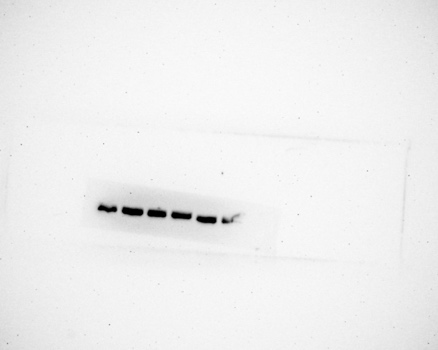

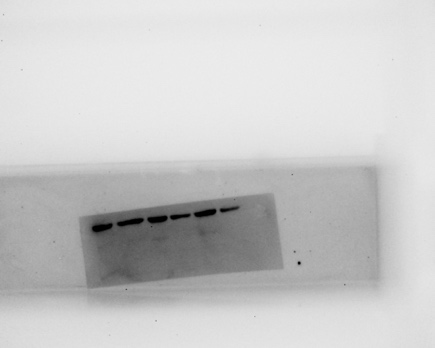


Beta-actin

Ampk

p-Ampk


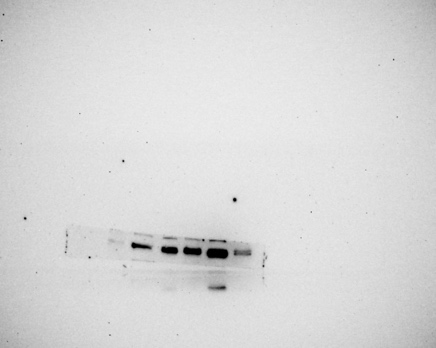

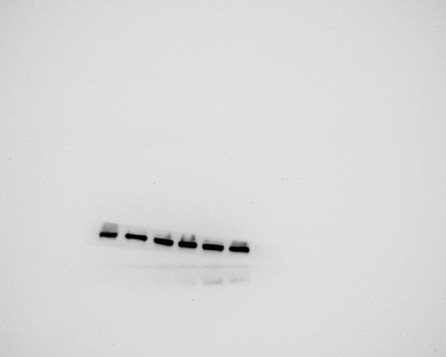

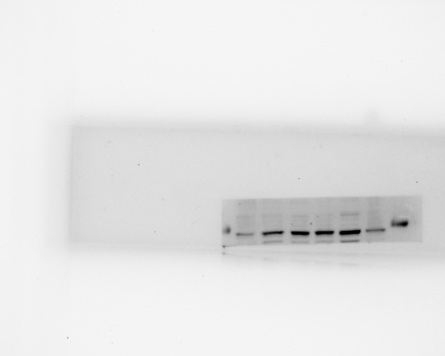


Marker

ER

EZH2

p-EZH2

Figure 5B (Right)


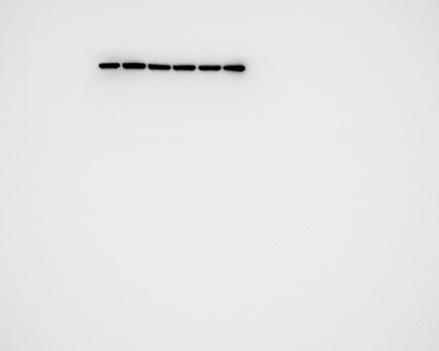

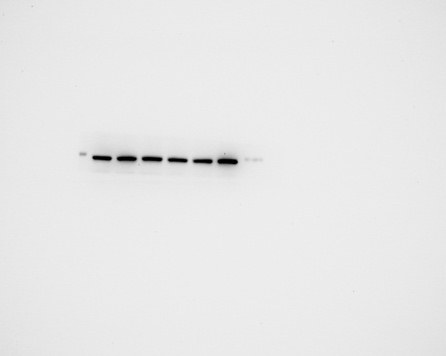

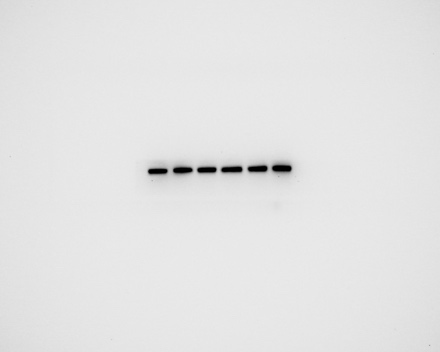


Marker

EZH2

Ampk

Beta-actin


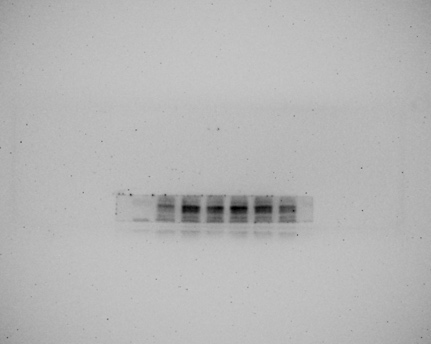

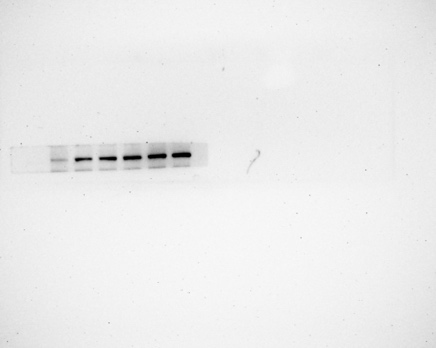

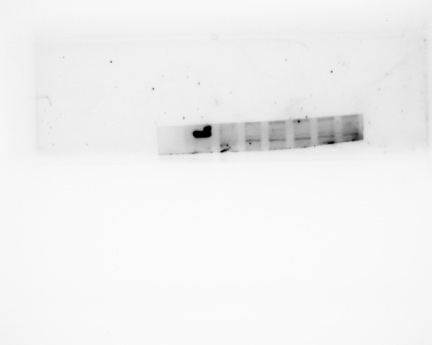


Marker

Marker

ER

p-EZH2

p-Ampk

Figure 5F


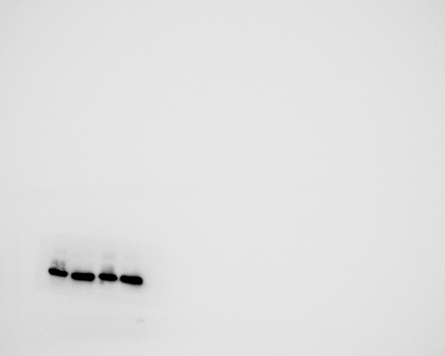

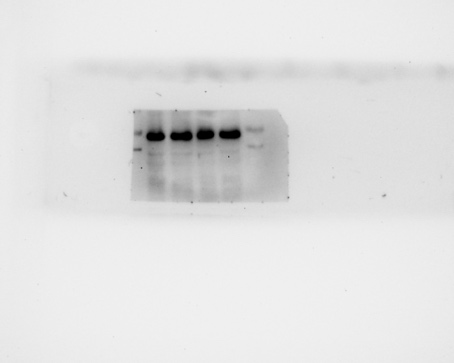

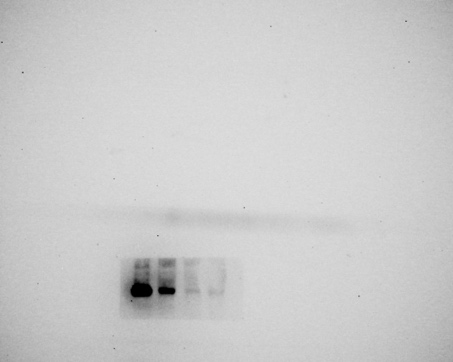


ER

Ampk

Beta-actin


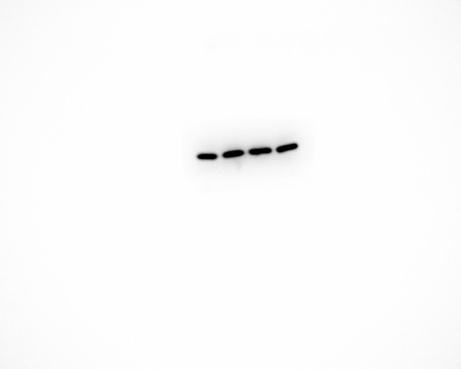

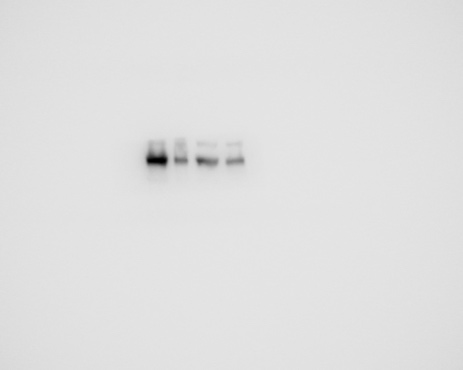

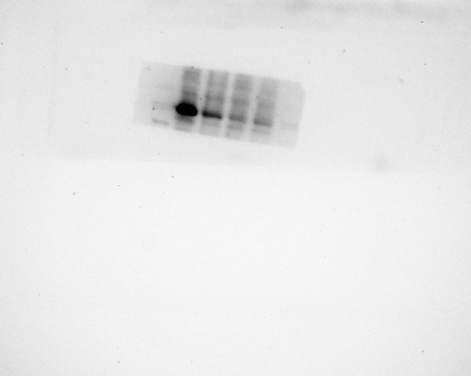

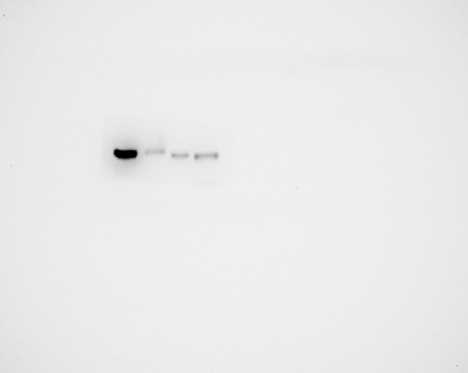


RAD51B

p-EZH2

p-Ampk

EZH2

Figure 5G


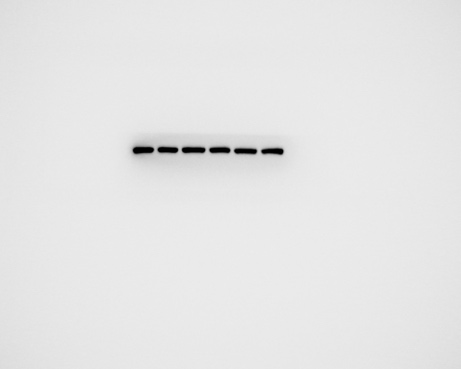

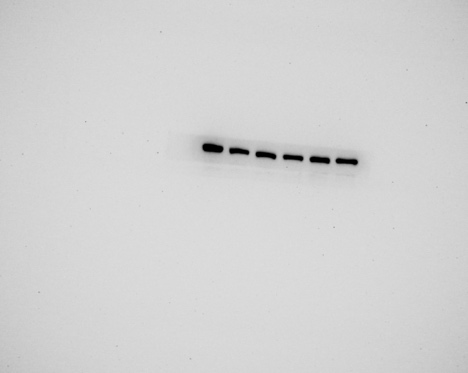


ER

Ampk

Beta-actin

p-EZH2

p-Ampk

EZH2

Figure 6A

Beta-actin

Ç√

Marker

ER

RAD51B

EZH2

H3k27me3

Histone 3

Figure 6B

Beta-actin

Histone 3

H3k27me3

EZH2

Ç√

Marker

RAD51B

ER

Supplementary Figure 3C

ER

Beta-actin

Supplementary Figure 6B (Left)

EZH2

Jarid2

SUZ12

RAD51B

Histone 3

Ç√

P-EZH2 (416)

Ç√

P-EZH2 (345)

AEBP2

Ç√

EZH1

Beta-actin

ER

H3k27me3

Supplementary Figure 6B (Right)

Histone3

EZH2

H3k27me3

ER

Ç√

P-EZH2 (345)

P-EZH2 (416)

Beta-actin

AEBP2

RAD51B

EZH1

Supplementary Figure 6G (Left)

Beta-actin

Marker

ER

AEBP2

Histone3

H3k27me3

RAD51B

Supplementary Figure 6G (Middle)

RAD51B

H3k27me3

ER

Histone3

Beta-actin

EZH2

Supplementary Figure 6G (Right)

ER

RAD51B

Histone3

H3k27me3

SUZ12

Beta-actin

Supplementary Figure 6I (Left)

ER

RAD51B

Beta-actin

Aebp2

Histone3

H3k27me3

Supplementary Figure 6I (Middle)

ER

EZH2ZH2

RAD51B

Beta-actin

Histone3

H3k27me3

Supplementary Figure 6I (Right)

SUZ12

RAD51B

ER

Beta-actin

Histone3

H3k27me3

Supplementary Figure 7C (UP)

Histone3

ER

RAD51B

H3k27me3

Beta-actin

Supplementary Figure 7C (DOWN)

Marker

RAD51B

Beta-actin

ER

H3k27me3

Histone3

Supplementary Figure 8A

HCC1937 cell line

Dose-dependent EPZ6438

GAPDH

Marker

Marker

ER

Dose-dependent GSK343

GAPDH

ER

Marker

Marker

Time-dependent EPZ6438

Marker

ER

GAPDH

Time-dependent GSK343

GAPDH

ER

EMT6 cell line

Dose-dependent EPZ6438

GAPDH

ER

Dose-dependent GSK343

GAPDH

ER

Marker

Time-dependent EPZ6438

ER

GAPDH

Time-dependent GSK343

GAPDH

ER

SUM149 cell line

Dose-dependent EPZ6438

GAPDH

ER

Dose-dependent GSK343

GAPDH

ER

Time-dependent EPZ6438

GAPDH

ER

Time-dependent GSK343

GAPDH

ER

4T1 cell line

Dose-dependent EPZ6438

Marker

Marker

ER

GAPDH

Dose-dependent GSK343

Marker

Marker

ER

GAPDH

Time-dependent EPZ6438

ER

GAPDH

Time-dependent GSK343

ER

GAPDH
