# Supplementary material for: RAD51B-EZH2 axis as a potential therapeutic target for TNBC through cell fate conversion
Source: Cell Death Dis. 2025 Nov 30;17(1):64. doi: 10.1038/s41419-025-08259-8 (PMC12827460; doi:10.1038/s41419-025-08259-8)
Supplement: Supplementary file 3 — Table S1. Sequence for sgRNA [file 41419_2025_8259_MOESM3_ESM.docx]

**Supplementary Table 1. Sequence for sgRNA**

| sgRNAs | sequence |
| --- | --- |
| eGFP | caccgGGGCGAGGAGCTGTTCACCG |
|  | aaacCGGTGAACAGCTCCTCGCCCc |
| Mouse Rad51b #1 | caccgCGTTTGCATCTGCGGGGCAC |
|  | aaacGTGCCCCGCAGATGCAAACGc |
| Mouse Rad51b #3 | caccgACCGTGCAATGCTTCATCCA |
|  | aaacTGGATGAAGCATTGCACGGTc |
| Mouse Ezh2 | caccgGCACCGAGGCGACTGCATTC |
|  | aaacGAATGCAGTCGCCTCGGTGCc |
| Mouse Suz12 | caccgGCCGGTGAAGAAGCCGAAAA |
|  | aaacTTTTCGGCTTCTTCACCGGCc |
| Mouse Aebp2 | caccgCCTGGAGATGTCGTCCGACG |
|  | aaacCGTCGGACGACATCTCCAGGc |
| Human Ezh2 | caccgACACGCTTCCGCCAACAAAC |
|  | aaacGTTTGTTGGCGGAAGCGTGTc |
| Human Suz12 | caccgCGGCTTCGGGGGTTCGGCGG |
|  | aaacCCGCCGAACCCCCGAAGCCGc |
| Human Aebp2 | caccgTGTGCATCGAAGAAATCATG |
|  | aaacCATGATTTCTTCGATGCACAc |
| Human Rad51b#1 | caccgGTGACTGGTCTGAGTTATCG |
|  | aaacCGATAACTCAGACCAGTCACc |
| Human Rad51b#2 | caccgACATTACCCACCAACATGGG |
|  | aaacCCCATGTTGGTGGGTAATGTc |
| Human Sik3#1 | caccgAGTTCAGGTGCAGCATAGGG |
|  | aaacCCCTATGCTGCACCTGAACTc |
| Human Sik3#2 | caccgGCACCAGTCAATATCCAGGT |
|  | aaacACCTGGATATTGACTGGTGCc |
| Human Notch1#1 | caccgGGCACCTGCCACAACGAGGT |
|  | aaacACCTCGTTGTGGCAGGTGCCc |
| Human Notch1#2 | caccgCGGCAGGATGTCAACGAGTG |
|  | aaacCACTCGTTGACATCCTGCCGc |
| Human Cntn6#1 | caccgAGCACAGTATCTGTCCGAGA |
|  | aaacTCTCGGACAGATACTGTGCTc |
| Human Cntn6#2 | caccgTTACTATGAGTTATCACTAC |
|  | aaacGTAGTGATAACTCATAGTAAc |
| Human Slc25a21#1 | caccgTGAGCAGTACAAGAAATTGC |
|  | aaacGCAATTTCTTGTACTGCTCAc |
| Human Slc25a21#2 | caccgCCTGATGCACCCCCTAGATG |
|  | aaacCATCTAGGGGGTGCATCAGGc |
| Human Sgcz#1 | caccgTGTTTCCCAGGATAGTCCGC |
|  | aaacGCGGACTATCCTGGGAAACAc |
| Human Sgcz#2 | caccgTTAACCGGACAGCTGACCAT |
|  | aaacATGGTCAGCTGTCCGGTTAAc |
| Human Jup#1 | caccgCATGGCCTCCCGCACCCGTT |
|  | aaacAACGGGTGCGGGAGGCCATGc |
| Human Jup#2 | caccgAGTTACGCATGATCTGCACG |
|  | aaacCGTGCAGATCATGCGTAACTc |
| Human Rbms3#1 | caccgTCTCAAGGCAAATGGCGTGC |
|  | aaacGCACGCCATTTGCCTTGAGAc |
| Human Rbms3#2 | caccgTTGCTGTGCAAATTCGCTGA |
|  | aaacTCAGCGAATTTGCACAGCAAc |
| Human Cntn5#1 | caccgTATCGCTACAGTTTGATAGA |
|  | aaacTCTATCAAACTGTAGCGATAc |
| Human Cntn5#2 | caccgTTCCTAAGATGGCTTCGAAA |
|  | aaacTTTCGAAGCCATCTTAGGAAc |
| Human Gucy1a2#1 | caccgCGGGTCAACCTGGACTCGCT |
|  | aaacAGCGAGTCCAGGTTGACCCGc |
| Human Gucy1a2#2 | caccgCCGCCGGCAGCATGTCTCGA |
|  | aaacTCGAGACATGCTGCCGGCGGc |
| Human Nrxn3#1 | caccgGGCCGACTCACCCCTTTCGC |
|  | aaacGCGAAAGGGGTGAGTCGGCCc |
| Human Nrxn3#2 | caccgATCCGCGACCTATTCATTGA |
|  | aaacTCAATGAATAGGTCGCGGATc |
| Human Opcml#1 | caccgGCACCTTACCCTTGACTGAC |
|  | aaacGTCAGTCAAGGGTAAGGTGCc |
| Human Opcml#2 | caccgGGTGTTCCCAAGCTTGTTCG |
|  | aaacCGAACAAGCTTGGGAACACCc |
| Human Csmd1#1 | caccgGCATAGTTCGGATACCCGTG |
|  | aaacCACGGGTATCCGAACTATGCc |
| Human Csmd1#2 | caccgCTCGCCCGTGATGATGATCC |
|  | aaacGGATCATCATCACGGGCGAGc |
| Human Hs6st3#1 | caccgTTCTAACGTGGAGATCAACG |
|  | aaacCGTTGATCTCCACGTTAGAAc |
| Human Hs6st3#2 | caccgCACCACTTTCGGCCGGCACC |
|  | aaacGGTGCCGGCCGAAAGTGGTGc |
| Human Vps13b#1 | caccgCAGCAAGCTCGAGTTAAAGT |
|  | aaacACTTTAACTCGAGCTTGCTGc |
| Human Vps13b#2 | caccgAGTGAAAGCTGTAGATCCGA |
|  | aaacTCGGATCTACAGCTTTCACTc |
| Human Trps1#1 | caccgGGATATGTATGCAACGCGTG |
|  | aaacCACGCGTTGCATACATATCCc |
| Human Trps1#2 | caccgATCCTACCCGTAACAGGGAC |
|  | aaacGTCCCTGTTACGGGTAGGATc |
| Human Auts2#1 | caccgTTTCTCGATCTGAGCTGTAG |
|  | aaacCTACAGCTCAGATCGAGAAAc |
| Human Auts2#2 | caccgGAAGTAGCCTGTTCCCAAGC |
|  | aaacGCTTGGGAACAGGCTACTTCc |
| Human Ptprd#1 | caccgTCGAACTCAACATTCGTGCC |
|  | aaacGGCACGAATGTTGAGTTCGAc |
| Human Ptprd#2 | caccgAAGCGCAGAAGCATCCGTTA |
|  | aaacTAACGGATGCTTCTGCGCTTc |
| Human Ptprk#1 | caccgAGGGGCCTGTGATTACCACC |
|  | aaacGGTGGTAATCACAGGCCCCTc |
| Human Ptprk#2 | caccgATGCTGTGCATAACAAGTTA |
|  | aaacTAACTTGTTATGCACAGCATc |
| Human Arhgap26#1 | caccgTCCCTTGGAGAAGTTTCGAA |
|  | aaacTTCGAAACTTCTCCAAGGGAc |
| Human Arhgap26#2 | caccgCCAGATTGAGAATGCCAGCG |
|  | aaacCGCTGGCATTCTCAATCTGGc |
| Human Esrrg#1 | caccgGTGCGTCTTGACAGAGTACG |
|  | aaacCGTACTCTGTCAAGACGCACc |
| Human Esrrg#2 | caccgGCGCAGACGTAAATCCTGCC |
|  | aaacGGCAGGATTTACGTCTGCGCc |
| Human Met#1 | caccgATGTGGCTGTCAGCATAAGT |
|  | aaacACTTATGCTGACAGCCACATc |
| Human Met#2 | caccgGACCTCACCATAGCTAATCT |
|  | aaacAGATTAGCTATGGTGAGGTCc |
| Human Slc4a4#1 | caccgGTTGACCATCAGATTGAGAC |
|  | aaacGTCTCAATCTGATGGTCAACc |
| Human Slc4a4#2 | caccgAGCCTCCCGATCAAGCATGA |
|  | aaacTCATGCTTGATCGGGAGGCTc |
| Human Ptprt#1 | caccgACTACTTCTCCAGCCGTGAC |
|  | aaacGTCACGGCTGGAGAAGTAGTc |
| Human Ptprt#2 | caccgGGCCTTGAACGTCTACGTGA |
|  | aaacTCACGTAGACGTTCAAGGCCc |
| Human Arhgef11#1 | caccgCCGGTGTGAAAGAGGGCGAC |
|  | aaacGTCGCCCTCTTTCACACCGGc |
| Human Arhgef11#2 | caccgCATCCGAAGGCTGGCGATGG |
|  | aaacCCATCGCCAGCCTTCGGATGc |
| Human Nedd4l#1 | caccgGAAACTTTCATTGTACGTAG |
|  | aaacCTACGTACAATGAAAGTTTCc |
| Human Nedd4l#2 | caccgCTCGCCAAAAAGGACATCTT |
|  | aaacAAGATGTCCTTTTTGGCGAGc |
| Human Nrg3#1 | caccgAGTCCTTGATCAGCCGCTTG |
|  | aaacCAAGCGGCTGATCAAGGACTc |
| Human Nrg3#2 | caccgCTTCAAACCCTGCCGAGACA |
|  | aaacTGTCTCGGCAGGGTTTGAAGc |
| Human Pcnx#1 | caccgTGCTGCTGTTCCAAGACGCG |
|  | aaacCGCGTCTTGGAACAGCAGCAc |
| Human Pcnx#2 | caccgGGATCTGGTTCCTCGCGTCT |
|  | aaacAGACGCGAGGAACCAGATCCc |
| Human Stpg2#1 | caccgACTATTATTTCATATAGTCG |
|  | aaacCGACTATATGAAATAATAGTc |
| Human Stpg2#2 | caccgTCTGAAACATTATAGTGTCC |
|  | aaacGGACACTATAATGTTTCAGAc |
| Human Magi2#1 | caccgGTAACAGACCAGATACTTCC |
|  | aaacGGAAGTATCTGGTCTGTTACc |
| Human Magi2#2 | caccgTGACAACCTCTACCTCCGCA |
|  | aaacTGCGGAGGTAGAGGTTGTCAc |
| Human Thsd7b#1 | caccgTTGACTTCACCGCGAGCGTA |
|  | aaacTACGCTCGCGGTGAAGTCAAc |
| Human Thsd7b#2 | caccgGTGCTTGTTCCTTACGCTCG |
|  | aaacCGAGCGTAAGGAACAAGCACc |
| Human Robo2#1 | caccgGGACTTTCCCCCGCGGATTG |
|  | aaacCAATCCGCGGGGGAAAGTCCc |
| Human Robo2#2 | caccgTGTAGGATCGCGTCTTCGCC |
|  | aaacGGCGAAGACGCGATCCTACAc |
| Human Gpc5#1 | caccgTTACCTGCCCGCGGCGAATC |
|  | aaacGATTCGCCGCGGGCAGGTAAc |
| Human Gpc5#2 | caccgCCGAGAGTTCTTCCAACGAC |
|  | aaacGTCGTTGGAAGAACTCTCGGc |
| Human Dpyd#1 | caccgGACATCAAGCACACGACTCT |
|  | aaacAGAGTCGTGTGCTTGATGTCc |
| Human Dpyd#2 | caccgGTACCCCAATCGAGCCAAAA |
|  | aaacTTTTGGCTCGATTGGGGTACc |
| Human Negr1#1 | caccgGTCTGAACAGAACACGTGTA |
|  | aaacTACACGTGTTCTGTTCAGACc |
| Human Negr1#2 | caccgGAAGTGCTCCTGTGTCACGT |
|  | aaacACGTGACACAGGAGCACTTCc |
| Human Dnm3#1 | caccgGCAGAAACAGATCGCGTGAC |
|  | aaacGTCACGCGATCTGTTTCTGCc |
| Human Dnm3#2 | caccgGTTACAATGCCCGACCCTCG |
|  | aaacCGAGGGTCGGGCATTGTAACc |
| Human Nlgn1#1 | caccgAATATAGGCAGTCTTCGCTC |
|  | aaacGAGCGAAGACTGCCTATATTc |
| Human Nlgn1#2 | caccgATCACAGTCAACTATCGACT |
|  | aaacAGTCGATAGTTGACTGTGATc |
| Human Mast4#1 | caccgGGACGCGGCGACCAGCGCAG |
|  | aaacCTGCGCTGGTCGCCGCGTCCc |
| Human Mast4#2 | caccgGACTCGGCCGAGGAAGCACC |
|  | aaacGGTGCTTCCTCGGCCGAGTCc |
| Human Lpp#1 | caccgTTCCAACAGGGGAATCCCGG |
|  | aaacCCGGGATTCCCCTGTTGGAAc |
| Human Lpp#2 | caccgTCACCAAAGTATTCGTCAGC |
|  | aaacGCTGACGAATACTTTGGTGAc |
| Human Exoc4#1 | caccgAACTTGTCATCAGTGCATCC |
|  | aaacGGATGCACTGATGACAAGTTc |
| Human Exoc4#2 | caccgACGTCACGAAGGATGTCTTG |
|  | aaacCAAGACATCCTTCGTGACGTc |
| Human Astn2#1 | caccgCCGGATGGGCACGCCAATGA |
|  | aaacTCATTGGCGTGCCCATCCGGc |
| Human Astn2#2 | caccgGCTCGTTACGCACAAAGAGC |
|  | aaacGCTCTTTGTGCGTAACGAGCc |
| Human Nfia#1 | caccgTATCCGACCCGAATATCGAG |
|  | aaacCTCGATATTCGGGTCGGATAc |
| Human Nfia#2 | caccgCGTGGGCACGTAGAGAACCG |
|  | aaacCGGTTCTCTACGTGCCCACGc |
| Human Gphn#1 | caccgAATCTTGCAGAAGACCGCAG |
|  | aaacCTGCGGTCTTCTGCAAGATTc |
| Human Gphn#2 | caccgTAGTGAGTGATAGTTGCTTC |
|  | aaacGAAGCAACTATCACTCACTAc |
| Human Dcaf5#1 | caccgGCTCATGAAGATGCAGTATA |
|  | aaacTATACTGCATCTTCATGAGCc |
| Human Dcaf5#2 | caccgCTTAAGGAGGAGATGACCGC |
|  | aaacGCGGTCATCTCCTCCTTAAGc |
| Human Rbfox1#1 | caccgTCTGAGCGCTCGTGTCCGCC |
|  | aaacGGCGGACACGAGCGCTCAGAc |
| Human Rbfox1#2 | caccgCACCCCGCGCCAGAGTACAC |
|  | aaacGTGTACTCTGGCGCGGGGTGc |
| Human Thsd4#1 | caccgCTGGCGGCTGGCGTCCGTAC |
|  | aaacGTACGGACGCCAGCCGCCAGc |
| Human Thsd4#2 | caccgGGTGTCGGCGGTGCGCACGT |
|  | aaacACGTGCGCACCGCCGACACCc |
| Human Snd1#1 | caccgCCCCTACGTAGGTCCTCTCA |
|  | aaacTGAGAGGACCTACGTAGGGGc |
| Human Snd1#2 | caccgGGAACGGTTCACATACTATC |
|  | aaacGATAGTATGTGAACCGTTCCc |
| Human Dab1#1 | caccgTTGATGAAGTTTCCGCAGCT |
|  | aaacAGCTGCGGAAACTTCATCAAc |
| Human Dab1#2 | caccgTTTCAACAGGGCGTTGTTGC |
|  | aaacGCAACAACGCCCTGTTGAAAc |
| Human Lrrc7#1 | caccgATTCAAGAAAGGCGTCATTC |
|  | aaacGAATGACGCCTTTCTTGAATc |
| Human Lrrc7#2 | caccgAGATTTGAAAGGTCGTTATC |
|  | aaacGATAACGACCTTTCAAATCTc |
| Human Nf1#1 | caccgTCTTTAGTCGCATTTCTACC |
|  | aaacGGTAGAAATGCGACTAAAGAc |
| Human Nf1#2 | caccgACACTGGAAAAATGTCTTGC |
|  | aaacGCAAGACATTTTTCCAGTGTc |
| Human Lipc#1 | caccgAATCTTGTGCGTTCCACCGA |
|  | aaacTCGGTGGAACGCACAAGATTc |
| Human Lipc#2 | caccgTTTGCCGGCAGTTCCATCGG |
|  | aaacCCGATGGAACTGCCGGCAAAc |
| Human Prkd1#1 | caccgCTTCAATAAGATCGCCTTCC |
|  | aaacGGAAGGCGATCTTATTGAAGc |
| Human Prkd1#2 | caccgCGACCAATAAACGACTCTGA |
|  | aaacTCAGAGTCGTTTATTGGTCGc |
| Human Vti1b#1 | caccgAGGGCACTGAAAGCCTGAAC |
|  | aaacGTTCAGGCTTTCAGTGCCCTc |
| Human Vti1b#2 | caccgTGCTGTAGAGAATGAGCATA |
|  | aaacTATGCTCATTCTCTACAGCAc |
| Human Plekhh1#1 | caccgGCGTTTCTGCCAGTCTACGC |
|  | aaacGCGTAGACTGGCAGAAACGCc |
| Human Plekhh1#2 | caccgAGGCGCCGGCCAGCGTAGAC |
|  | aaacGTCTACGCTGGCCGGCGCCTc |
| Human Jarid2#1 | caccgTTCAGCTAACCACCCCGCAG |
|  | aaacCTGCGGGGTGGTTAGCTGAAc |
| Human Jarid2#2 | caccgCTTCATGCCAGTCGACCCCC |
|  | aaacGGGGGTCGACTGGCATGAAGc |
| Human Dennd1a#1 | caccgCCCCACTTACCACGCTGAGA |
|  | aaacTCTCAGCGTGGTAAGTGGGGc |
| Human Dennd1a#2 | caccgAGTGTCTGTCCATCTCAGCG |
|  | aaacCGCTGAGATGGACAGACACTc |
| Human Rbm47#1 | caccgCGTGATCGTCTACGCCAGCG |
|  | aaacCGCTGGCGTAGACGATCACGc |
| Human Rbm47#2 | caccgGCCCAATGAGCGCGTTGTAG |
|  | aaacCTACAACGCGCTCATTGGGCc |
| Human Ppp2r5e#1 | caccgAGGGTAGGTTCATCTTCTTC |
|  | aaacGAAGAAGATGAACCTACCCTc |
| Human Ppp2r5e#2 | caccgAGAAGATGAACCTACCCTTG |
|  | aaacCAAGGGTAGGTTCATCTTCTc |
| Human Tbc1d5#1 | caccgACTAGCATATTACCAACCCG |
|  | aaacCGGGTTGGTAATATGCTAGTc |
| Human Tbc1d5#2 | caccgGATCAATAATCCTCTTTCAC |
|  | aaacGTGAAAGAGGATTATTGATCc |
| Human Ccser1#1 | caccgAGACTGCGATCCTCGTCAGA |
|  | aaacTCTGACGAGGATCGCAGTCTc |
| Human Ccser1#2 | caccgGGCGAAGCTCTTACTAATAC |
|  | aaacGTATTAGTAAGAGCTTCGCCc |
| Human Arhgef12#1 | caccgTTGACAGACTGTACGAAGAC |
|  | aaacGTCTTCGTACAGTCTGTCAAc |
| Human Arhgef12#2 | caccgTTCGTACAGTCTGTCAAAGA |
|  | aaacTCTTTGACAGACTGTACGAAc |
