# Supplementary material for: RAD51B-EZH2 axis as a potential therapeutic target for TNBC through cell fate conversion
Source: Cell Death Dis. 2025 Nov 30;17(1):64. doi: 10.1038/s41419-025-08259-8 (PMC12827460; doi:10.1038/s41419-025-08259-8)
Supplement: Supplementary file 4 — Table S2. Primers for RT-qPCR [file 41419_2025_8259_MOESM4_ESM.docx]

**Supplementary Table 2. Primers for RT-qPCR**

| qPCR primers | sequence |
| --- | --- |
| Mouse Gapdh F | CATCACTGCCACCCAGAAGACTG |
| Mouse Gapdh R | ATGCCAGTGAGCTTCCCGTTCAG |
| Mouse Esr1 F | TCTGCCAAGGAGACTCGCTACT |
| Mouse Esr1 R | GGTGCATTGGTTTGTAGCTGGAC |
| Mouse Egfr F | GGACTGTGTCTCCTGCCAGAAT |
| Mouse Egfr R | GGCAGACATTCTGGATGGCACT |
| Mouse Hbegf F | GAGTTCCGTACTCCCTCTTGCA |
| Mouse Hbegf R | CAGCCAAGACTGTAGTGTGGTC |
| Mouse Areg F | GCAGATACATCGAGAACCTGGAG |
| Mouse Areg R | CCTTGTCATCCTCGCTGTGAGT |
| Mouse Aebp2 F | GCAGACCACATTCGCTCCATAC |
| Mouse Aebp2 R | GCTGTAGCCAACTCTGACTGGT |
| Mouse Suz12 F | CTCTTCGATGGACAGGAGAAACC |
| Mouse Suz12 R | GCTCTGTAGTCAGCGTCTCCTT |
| Mouse Ezh2 F | CATACGCTCTTCTGTCGACGATG |
| Mouse Ezh2 R | ACACTGTGGTCCACAAGGCTTG |
| Mouse Serpine1 F | CCTCTTCCACAAGTCTGATGGC |
| Mouse Serpine1 R | GCAGTTCCACAACGTCATACTCG |
| Mouse Id2 F | TCACCAGAGACCTGGACAGAAC |
| Mouse Id2 R | TGCTATCATTCGACATAAGCTCAG |
| Mouse Rbms3 F | CTTCACCGTACAGTCTCGCAAC |
| Mouse Rbms3 R | ACCCACGATGTTAGTTGGCTGC |
| Mouse Bmp2 F | AACACCGTGCGCAGCTTCCATC |
| Mouse Bmp2 R | CGGAAGATCTGGAGTTCTGCAG |
| Mouse Hoxa9 F | GCCTTCTCCGAAAACAATGCCG |
| Mouse Hoxa9 R | TTCCGAGTGGAGCGAGCATGTA |
| Mouse Dab2ip F | CGAGACCCTTTCCAACACAGCA |
| Mouse Dab2ip R | GTGTGGACATCCCTCAGGATAC |
| Mouse Pgr F | CTACTCGCTGTGCCTTACCATG |
| Mouse Pgr R | CTGGCTTTGACTCCTCAGTCCT |
| Mouse Erbb2 F | GACCTCAGTGTCTTCCAGAACC |
| Mouse Erbb2 R | TGCGGTGAATGAGAGCCAATCC |
| Human ESR1 F | CTCTCCCACATCAGGCACA |
| Human ESR1 R | CTTTGGTCCGTCTCCTCCA |
| mEsr1 promoter F | ACTTGCGCTGCGCCTTCTCT |
| mEsr1 promoter R | ACTGCTGTCCCTCAGCAGAC |
| Human SERPINE1 F | CTCATCAGCCACTGGAAAGGCA |
| Human SERPINE1 R | GACTCGTGAAGTCAGCCTGAAAC |
| Human ID2 F | TTGTCAGCCTGCATCACCAGAG |
| Human ID2 R | AGCCACACAGTGCTTTGCTGTC |
| Human RBMS3 F | GTGTTGGCTTTGCCAGAATGGAG |
| Human RBMS3 R | CGCTTCTTTTGTCCTCCATCAGC |
| Human BMP2 F | TGTATCGCAGGCACTCAGGTCA |
| Human BMP2 R | CCACTCGTTTCTGGTAGTTCTTC |
| Human HOXA9 F | AGAATGAGAGCGGCGGAGACAA |
| Human HOXA9 R | CTCTTTCTCCAGTTCCAGGGTC |
| Human DAB2IP F | TCATCGCCAAGGTCACCCAGAA |
| Human DAB2IP R | CGCTGCATGTTGGTCCACTCAT |
