# Supplementary material for: RAD51B-EZH2 axis as a potential therapeutic target for TNBC through cell fate conversion
Source: Cell Death Dis. 2025 Nov 30;17(1):64. doi: 10.1038/s41419-025-08259-8 (PMC12827460; doi:10.1038/s41419-025-08259-8)
Supplement: Supplementary file 5 — Table S3. Key resource table [file 41419_2025_8259_MOESM5_ESM.docx]

## Supplementary Table 3. Key resources table

| REAGENT or RESOURCE | SOURCE | IDENTIFIER |
| --- | --- | --- |
| Antibodies | | |
| ERα | Abcam | Cat#ab241557 |
| PR | Abcam | Cat#ab101688; RRID: AB_10715248 |
| Her2 | Cell Signaling Technology | Cat#2165; RRID: AB_10692490 |
| RAD51B | Invitrogen | Cat#PA5-101336; RRID: AB_2850773 |
| H3K27me3 | Cell Signaling Technology | Cat#9733; RRID: AB_2616029 |
| β-actin | Cell Signaling Technology | Cat#4967; RRID: AB_330288 |
| γ-H2AX | Cell Signaling Technology | Cat#9718; RRID: AB_2118009 |
| AMPKα | Cell Signaling Technology | Cat#2532; RRID: AB_330331 |
| p-AMPKα (Thr 172) | Cell Signaling Technology | Cat#2535; RRID: AB_331250 |
| GAPDH | Cell Signaling Technology | Cat#5174; RRID: AB_10622025 |
| Histone 3 | Cell Signaling Technology | Cat#9715; RRID: AB_331563 |
| EZH2 | Cell Signaling Technology | Cat#5246; RRID: AB_10694683 |
| SUZ12 | Cell Signaling Technology | Cat#3737; RRID: AB_2196850 |
| AEBP2 | Cell Signaling Technology | Cat#14129; RRID: AB_2798398 |
| β-Tubulin | Cell Signaling Technology | Cat#2146; RRID: AB_2210545 |
| phosphor-Ezh2 (Thr345) | Affinity | Cat#AF3584; RRID: AB_2846898 |
| phosphor-Ezh2 (Thr416) | Affinity | Cat#AF3585; RRID: AB_2846899 |
| phosphor-Ezh2 (Thr311) | Cell Signaling Technology | Cat#27888; RRID: AB_2798950 |
| PR (for western blot) | Santa Cruz | Cat#sc-538; RRID: AB_632263 |
| Bacterial and virus strains | | |
| One Shot^TM^ Stbl3^TM^ Chemically Competent *E. coli* | Thermo Fisher Scientific | Cat#C737303 |
| Chemicals, peptides, and recombinant proteins | | |
| D-Luciferin Potassium Salt | Beyotime | Cat#ST196 |
| DAPI | Thermo Fisher Scientific | Cat#D1306 |
| Hoechst 33258 | Thermo Fisher Scientific | Cat#H3569 |
| Puromycin | InvivoGen | Cat#ant-pr-1 |
| Blasticidin | InvivoGen | Cat#ant-bl-05 |
| EPZ6438 | Selleckchem | Cat#S7128 |
| GSK343 | Selleckchem | Cat#S7164 |
| Antimycin A | Sigma-Aldrich | Cat#A8674 |
| estradiol | Sigma-Aldrich | Cat# E8875 |
| Rat Collagen I | R&D Systems | Cat#3440-100-01 |
| Hygromycin B | InvivoGen | Cat#ant-hg-5 |
| Lipofectamine^TM^ 3000 | Thermo Fisher Scientific | Cat#L3000075 |
| Critical commercial assays | | |
| DAB substrate kit | Abcam | Cat#ab64238 |
| BCA Protein Assay Kit | Thermo Fisher Scientific | Cat#A55864 |
| Reverse Transcriptase kit | Qiagen | Cat#205311 |
| FastStart SYBR Green Master Kit | Roche | Cat#12239264001 |
| Dual-Luciferase Reporter Assay System Kit | Promega | Cat#E1910 |
| ADP/ATP ratios assay kit | Sigma-Aldrich | Cat#MAK135 |
| ChIP Kit Magnetic-One-Step | Abcam | Cat#ab156907 |
| Chromatin Extraction Kit | Abcam | Cat#ab117152 |
| Deposited data | | |
| RNA-Seq | This paper | N/A |
| Experimental models: Cell lines | | |
| Human: MDA-MB-231 | ATCC | Cat#HTB-26 |
| Human: T47D | ATCC | Cat#HTB-133 |
| Human: MCF-7 | ATCC | Cat#HTB-22 |
| Human: HCC1937 | ATCC | Cat#CRL-2336 |
| Human: 293FT | Invitrogen | Cat#R70007 |
| Mouse: 4T1 | ATCC | Cat#CRL-2539 |
| Mouse: EMT6 | ATCC | Cat#CRL-2755 |
| Mouse: HP5008 | Zhou et al. ^22^ | N/A |
| Mouse: 545 | Xu et al. ^10^ | N/A |
| Mouse: B477 | Xu et al. ^48^ | N/A |
| Mouse: G600 | Xu et al. ^48^ | N/A |
| Experimental models: Organisms/strains | | |
| Mouse: BALB/c nude | Charles River | Cat#086 |
| Mouse: BALB/c | The Jackson Laboratory | Cat#000651 |
| Oligonucleotides | | |
| See Table S1 and Table S2 |  |  |
| Recombinant DNA | | |
| lentiCRISPRv2 Puro | Addgene | Cat#52961 |
| LentiCRISPRv2 blast | Addgene | Cat#98293 |
| LentiCRISPRv2 hygro | Addgene | Cat#98291 |
| TLCV2 | Addgene | Cat#87360 |
| ERE-Luc | Addgene | Cat#11354 |
| PRE-Luc | Addgene | Cat#11350 |
| pNeuLite | Addgene | Cat#16247 |
| p-GF-ERE-Luc | System Biosciences | Cat#TR205PA-P |
| Software and algorithms | | |
| GraphPad Prism | GraphPad | https://www.graphpad.com/ |
| ImageJ | National Institutes of Health | https://imagej.nih.gov/ij/ |
